# Supplementary material for: Protein entanglement misfolding influences whether proteins undergo proteasomal degradation or persist in near-native misfolded states
Source: Nat Commun. 2026 Aug 25;17:10135. doi: 10.1038/s41467-026-76875-9 (PMC13601551; doi:10.1038/s41467-026-76875-9)
Supplement: Supplementary file 1 [file 41467_2026_76875_MOESM1_ESM.pdf]

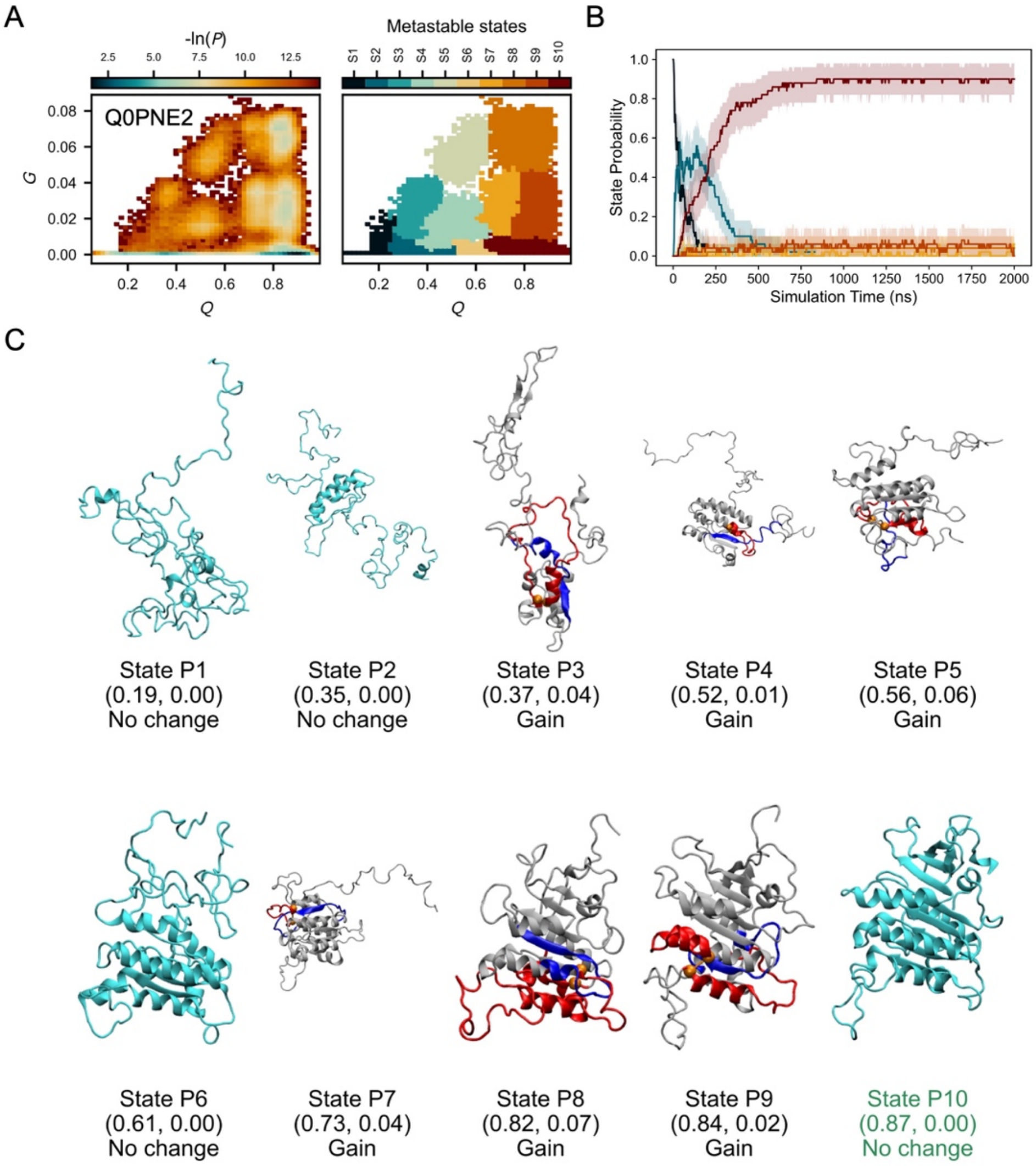

Figure S1. Simulation structural ensemble of the YU-E protein Q0PNE2. A. The probability distribution (-ln*P*) in the *Q* – *G* space (left) and the corresponding metastable states mapped onto structures from temperature-quench refolding simulations (right). B. Time evolution of metastable-state probabilities. Colors match the state assignments shown in panel A. Shaded regions denote 95% confidence intervals, estimated using bootstrap resampling (10^5 iterations). C. Representative structures of each metastable state predicted from simulations. *Q* and *G* values at the cluster center, as well as the type of entanglement change (gain, loss or no change), are shown below each structure image. Color scheme follows that used in Fig. 2B, 2D, and 2F.

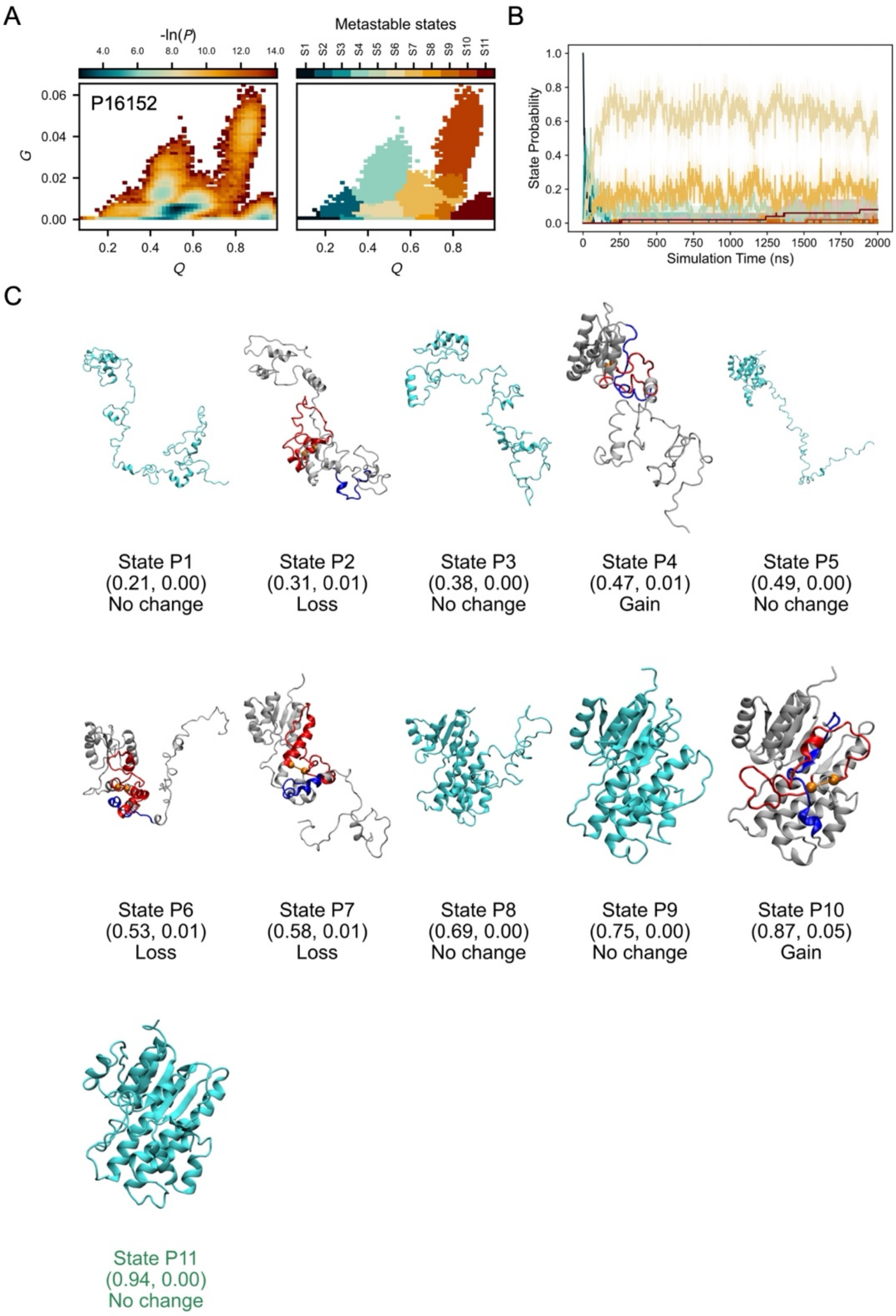

Figure S2. Simulation structural ensemble of the YU-E protein P16152. A. The probability distribution (-ln*P*) in the *Q* – *G* space (left) and the corresponding metastable states mapped onto structures from temperature-quench refolding simulations (right). B. Time evolution of metastable-state probabilities. Colors match the state assignments shown in panel A. Shaded regions denote 95% confidence intervals, estimated using bootstrap resampling (10^5 iterations). C. Representative structures of each metastable state predicted from simulations. *Q* and *G* values at the cluster center, as well as the type of entanglement change (gain, loss or no change), are shown below each structure image. Color scheme follows that used in Fig. 2B, 2D, and 2F.

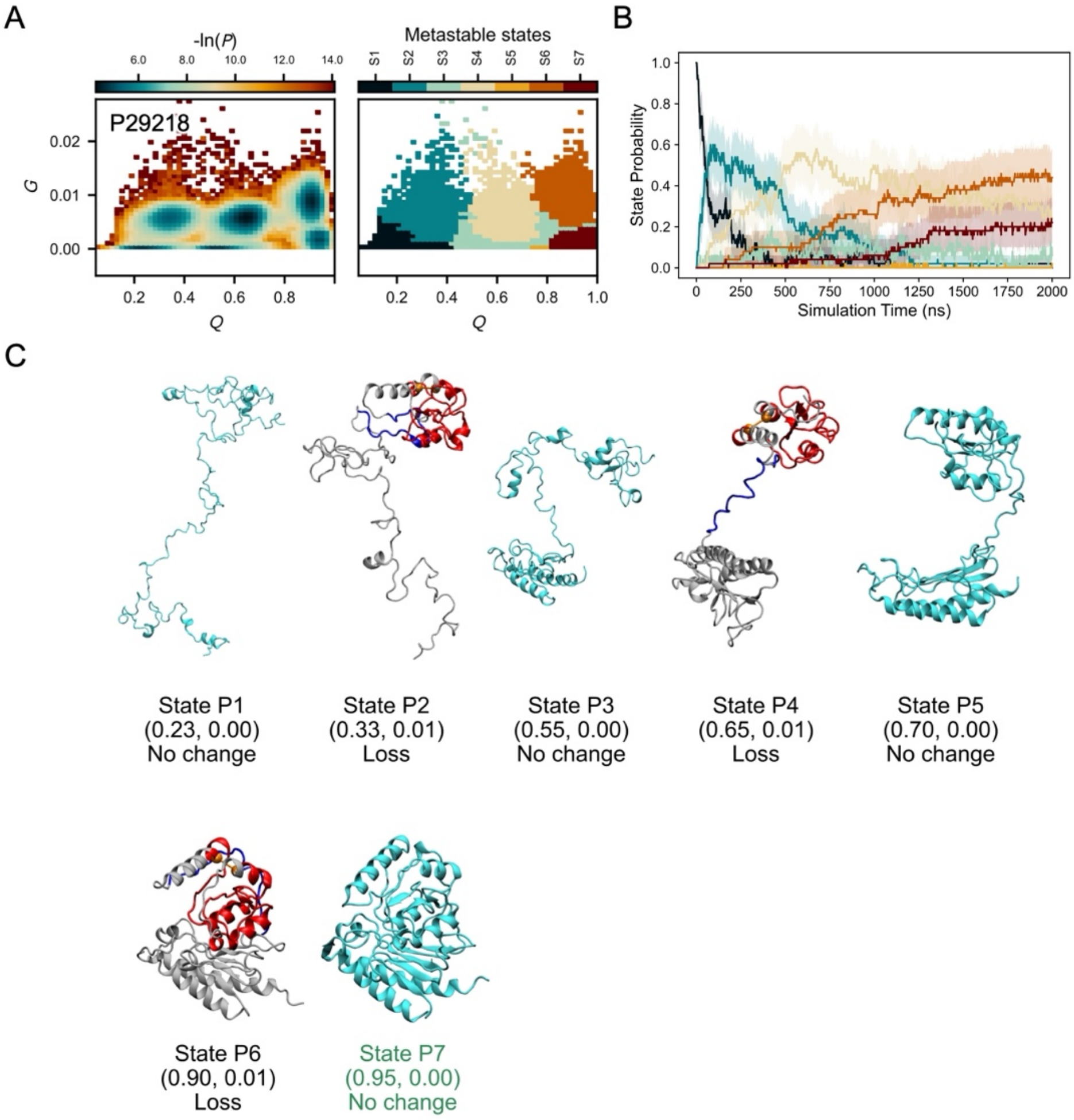

Figure S3. Simulation structural ensemble of the YU-E protein P29218. A. The probability distribution (-ln*P*) in the *Q* – *G* space (left) and the corresponding metastable states mapped onto structures from temperature-quench refolding simulations (right). B. Time evolution of metastable-state probabilities. Colors match the state assignments shown in panel A. Shaded regions denote 95% confidence intervals, estimated using bootstrap resampling (10^5 iterations). C. Representative structures of each metastable state predicted from simulations. *Q* and *G* values at the cluster center, as well as the type of entanglement change (gain, loss or no change), are shown below each structure image. Color scheme follows that used in Fig. 2B, 2D, and 2F.

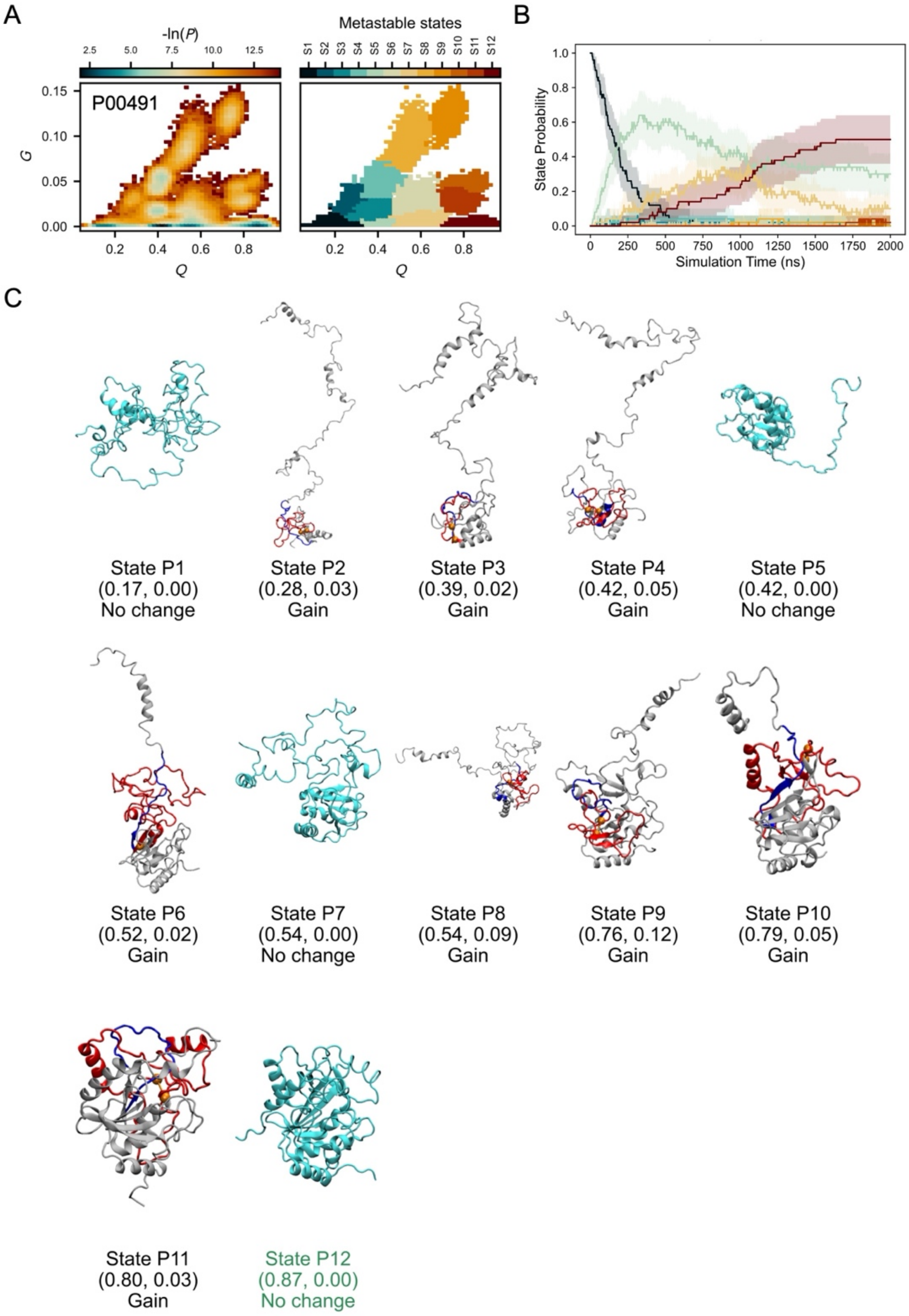

Figure S4. Simulation structural ensemble of the YU-E protein P00491. A. The probability distribution (-ln*P*) in the *Q* – *G* space (left) and the corresponding metastable states mapped onto structures from temperature-quench refolding simulations (right). B. Time evolution of metastable-state probabilities. Colors match the state assignments shown in panel A. Shaded regions denote 95% confidence intervals, estimated using bootstrap resampling (10^5 iterations). C. Representative structures of each metastable state predicted from simulations. *Q* and *G* values at the cluster center, as well as the type of entanglement change (gain, loss or no change), are shown below each structure image. Color scheme follows that used in Fig. 2B, 2D, and 2F.

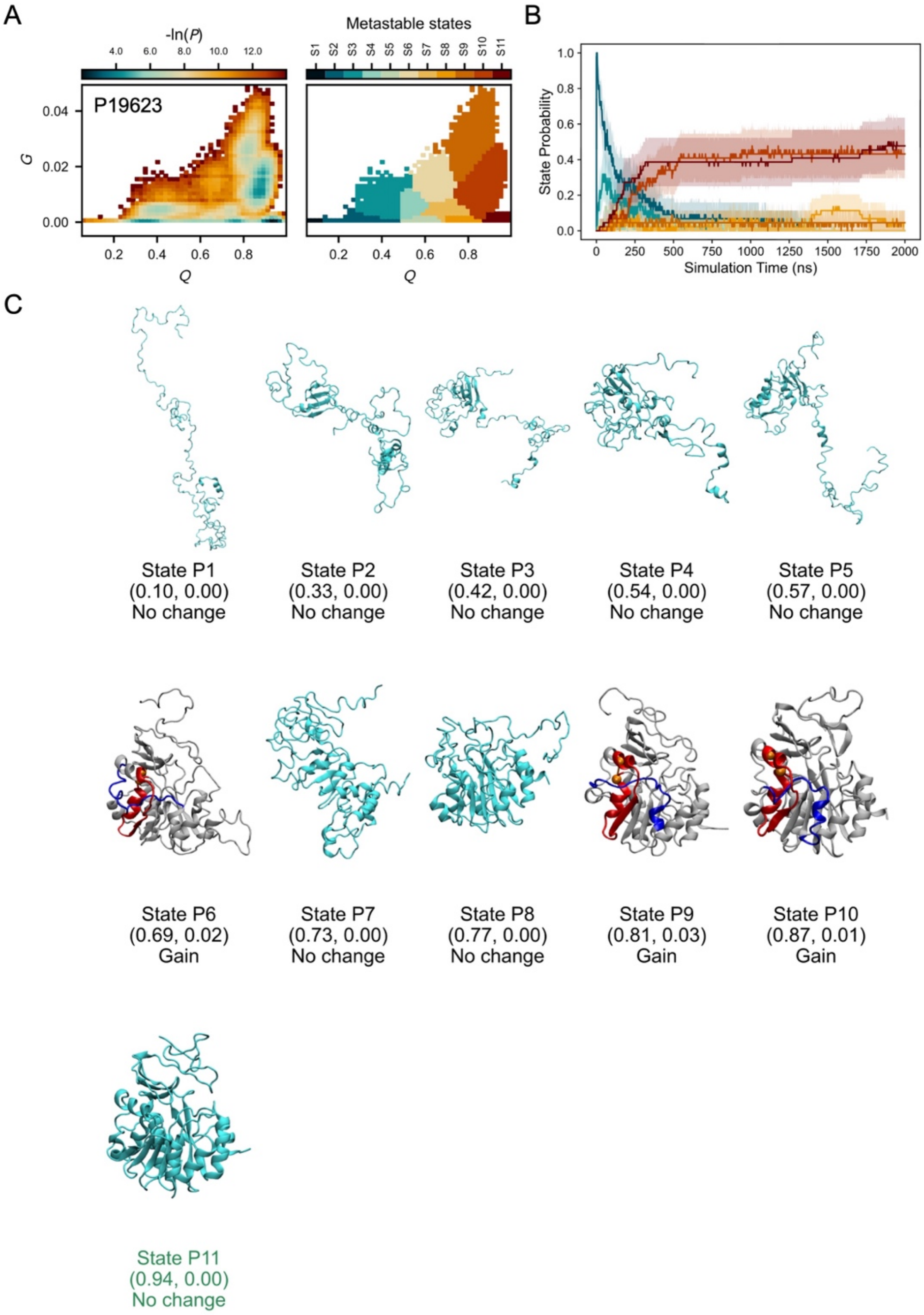

Figure S5. Simulation structural ensemble of the YU-E protein P19623. A. The probability distribution (-ln*P*) in the *Q* – *G* space (left) and the corresponding metastable states mapped onto structures from temperature-quench refolding simulations (right). B. Time evolution of metastable-state probabilities. Colors match the state assignments shown in panel A. Shaded regions denote 95% confidence intervals, estimated using bootstrap resampling (10^5 iterations). C. Representative structures of each metastable state predicted from simulations. *Q* and *G* values at the cluster center, as well as the type of entanglement change (gain, loss or no change), are shown below each structure image. Color scheme follows that used in Fig. 2B, 2D, and 2F.

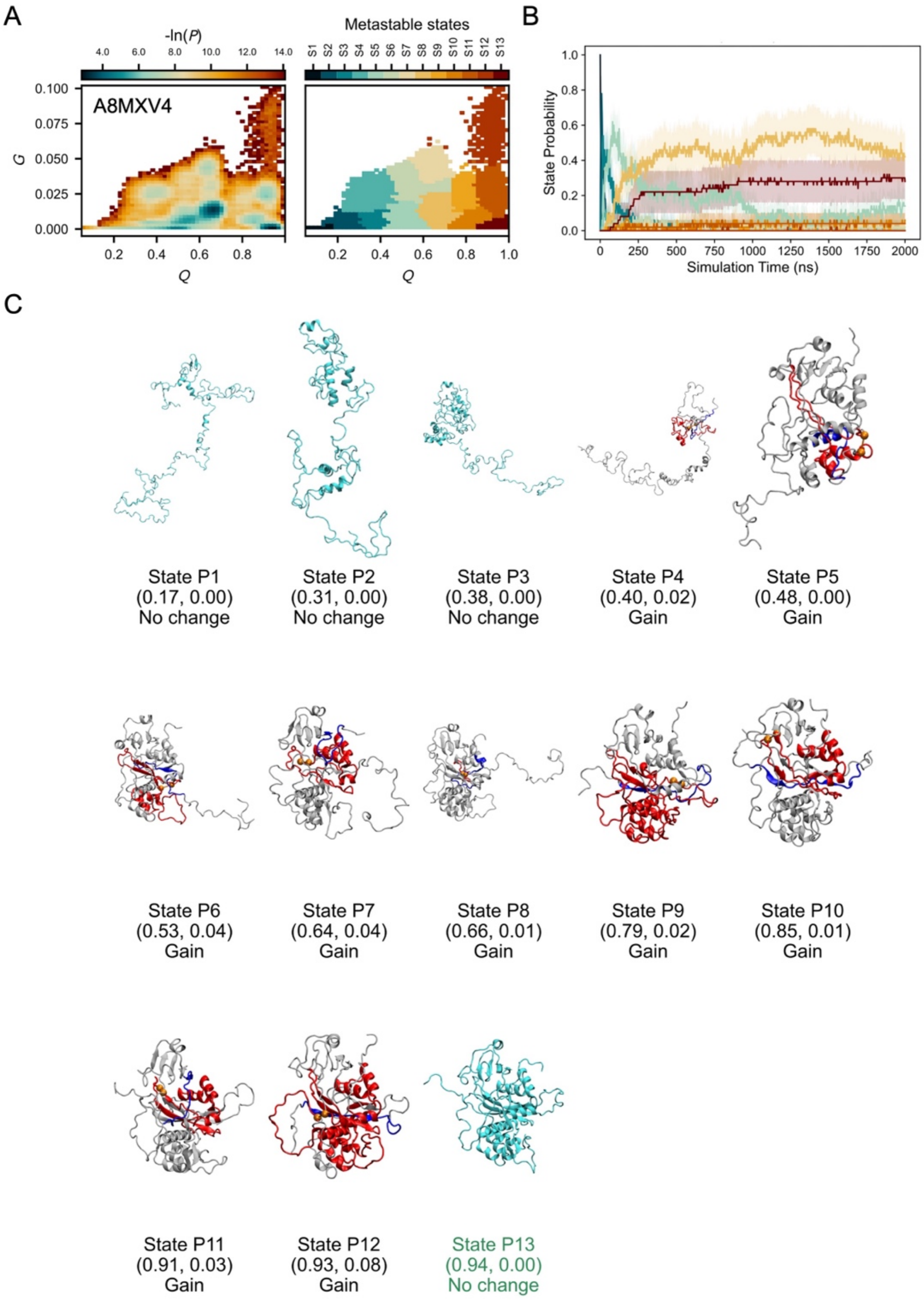

Figure S6. Simulation structural ensemble of the YU-E protein A8MXV4. A. The probability distribution (-ln*P*) in the *Q* – *G* space (left) and the corresponding metastable states mapped onto structures from temperature-quench refolding simulations (right). B. Time evolution of metastable-state probabilities. Colors match the state assignments shown in panel A. Shaded regions denote 95% confidence intervals, estimated using bootstrap resampling (10^5 iterations). C. Representative structures of each metastable state predicted from simulations. *Q* and *G* values at the cluster center, as well as the type of entanglement change (gain, loss or no change), are shown below each structure image. Color scheme follows that used in Fig. 2B, 2D, and 2F.

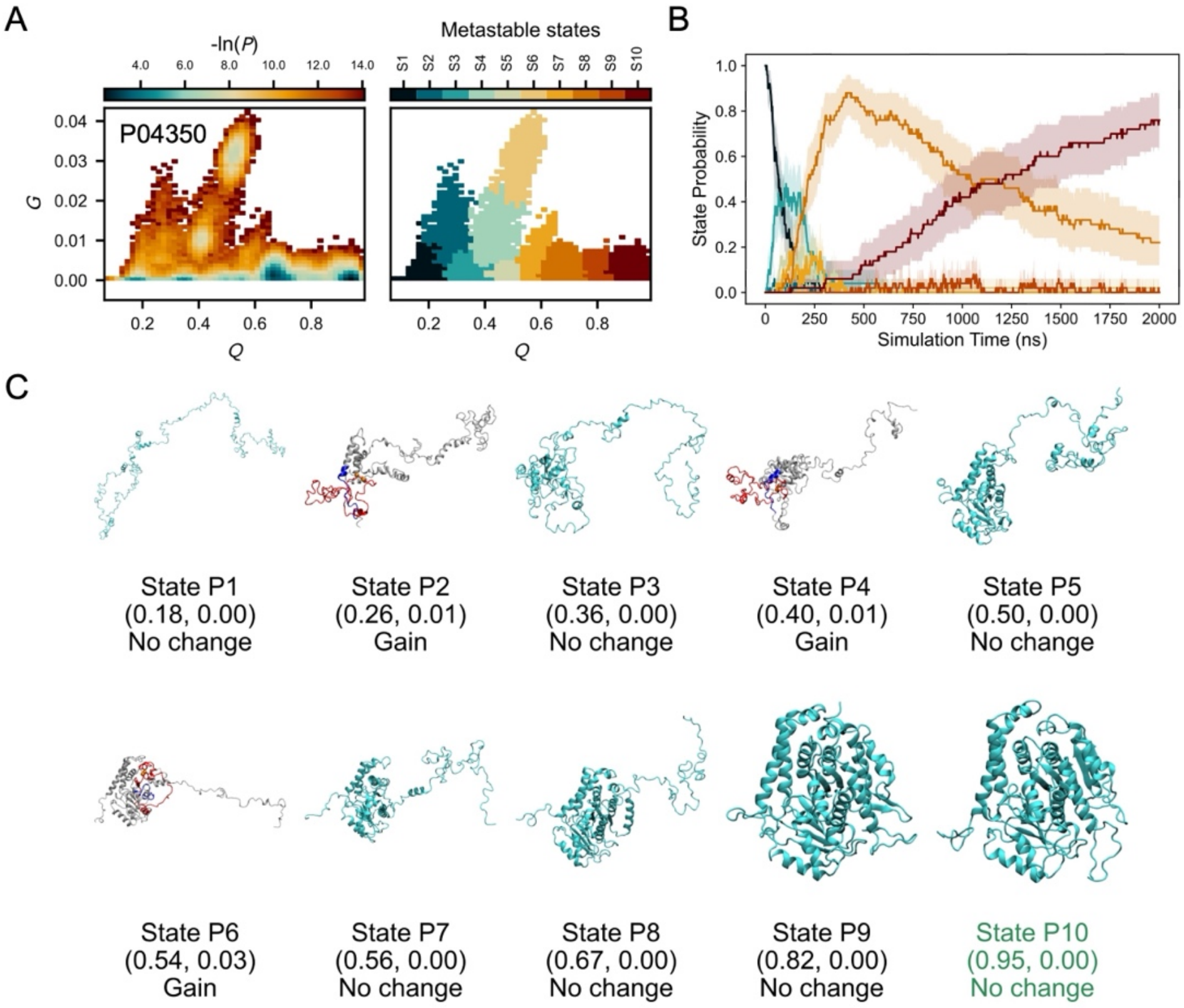

Figure S7. Simulation structural ensemble of the YU-E protein P04350. A. The probability distribution (-ln*P*) in the *Q* – *G* space (left) and the corresponding metastable states mapped onto structures from temperature-quench refolding simulations (right). B. Time evolution of metastable-state probabilities. Colors match the state assignments shown in panel A. Shaded regions denote 95% confidence intervals, estimated using bootstrap resampling (10^5 iterations). C. Representative structures of each metastable state predicted from simulations. *Q* and *G* values at the cluster center, as well as the type of entanglement change (gain, loss or no change), are shown below each structure image. Color scheme follows that used in Fig. 2B, 2D, and 2F.

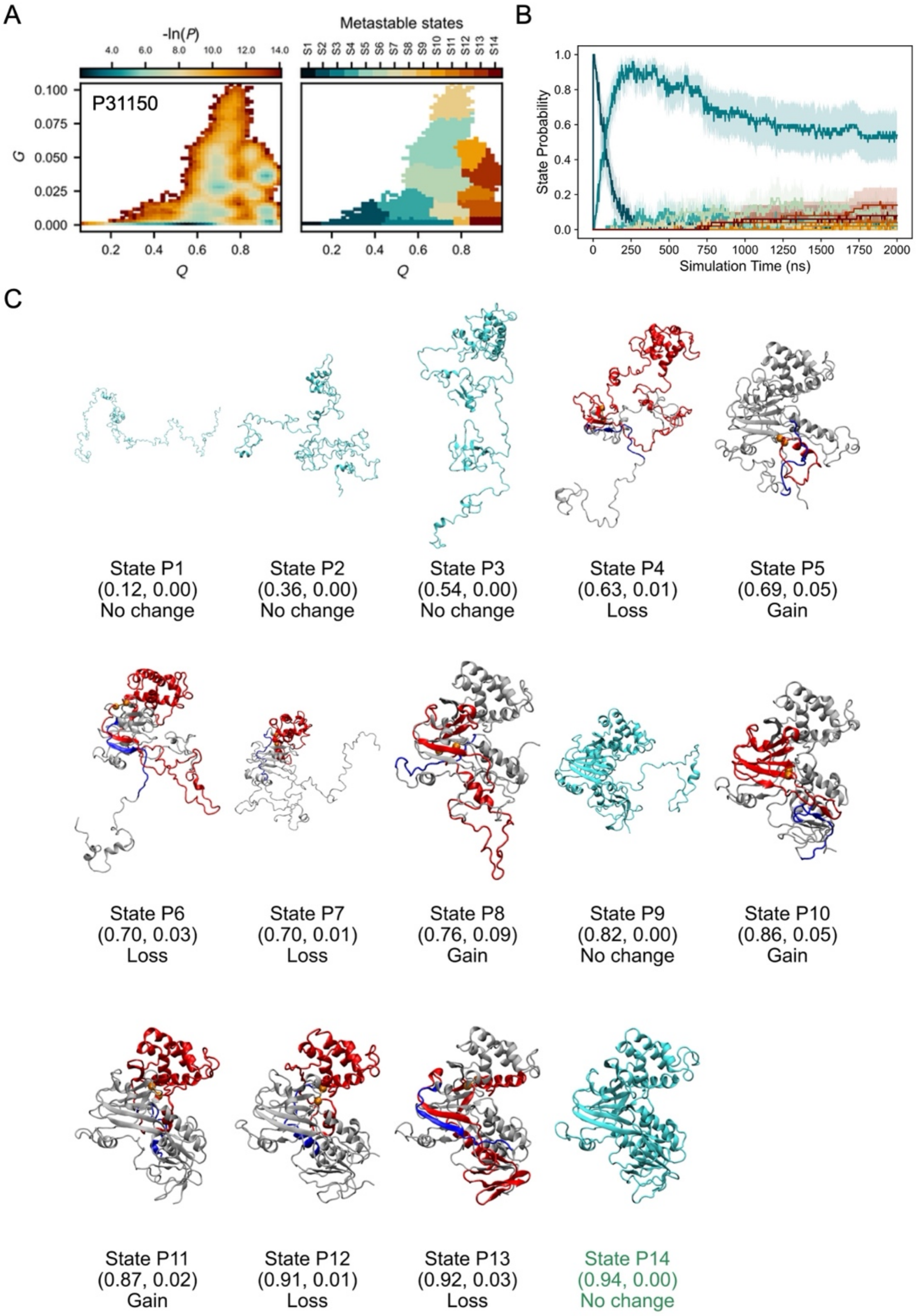

Figure S8. Simulation structural ensemble of the YU-E protein P31150. A. The probability distribution (-ln*P*) in the *Q* – *G* space (left) and the corresponding metastable states mapped onto structures from temperature-quench refolding simulations (right). B. Time evolution of metastable-state probabilities. Colors match the state assignments shown in panel A. Shaded regions denote 95% confidence intervals, estimated using bootstrap resampling (10^5 iterations). C. Representative structures of each metastable state predicted from simulations. *Q* and *G* values at the cluster center, as well as the type of entanglement change (gain, loss or no change), are shown below each structure image. Color scheme follows that used in Fig. 2B, 2D, and 2F.

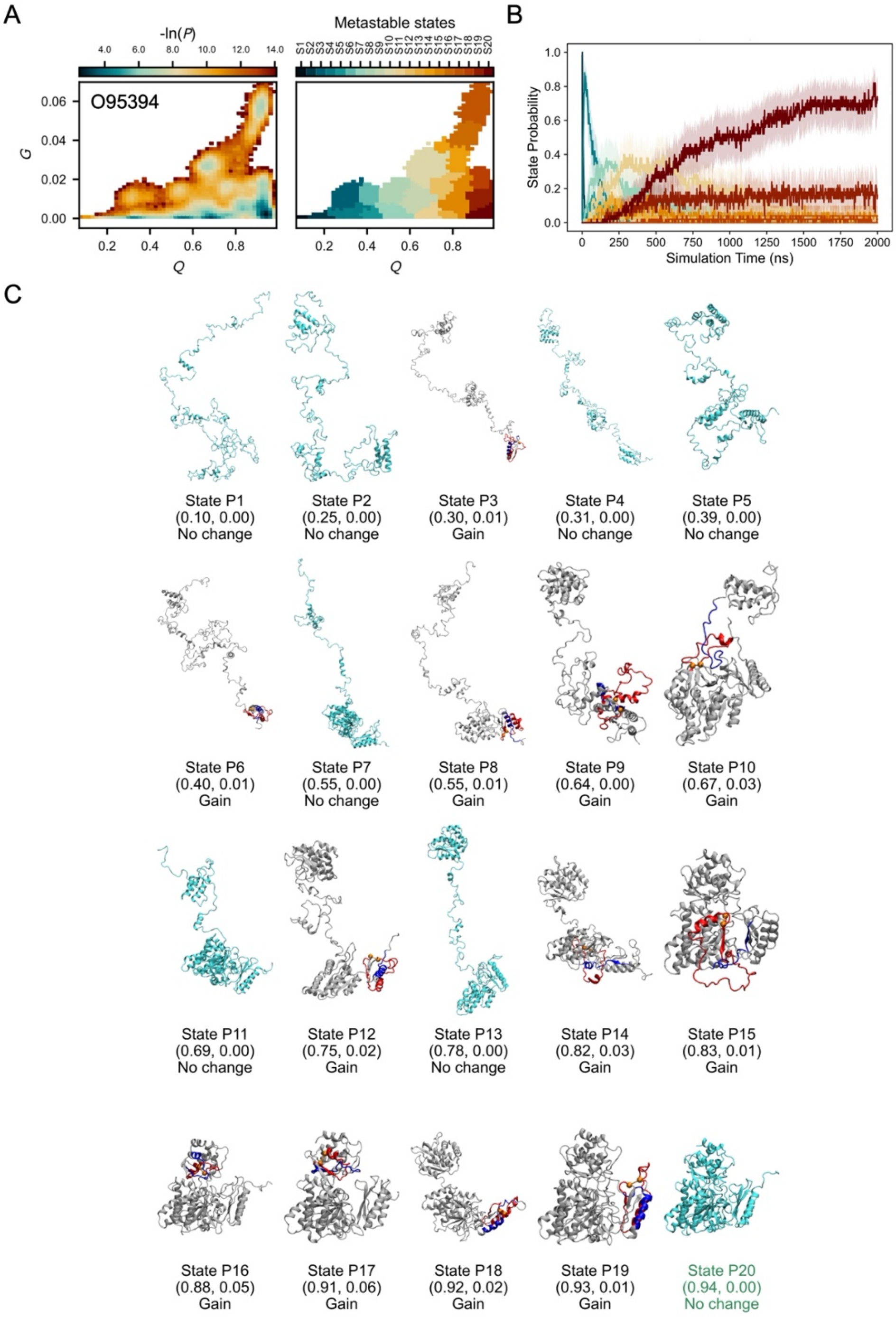

Figure S9. Simulation structural ensemble of the YU-E protein O95394. A. The probability distribution (-ln*P*) in the *Q* – *G* space (left) and the corresponding metastable states mapped onto structures from temperature-quench refolding simulations (right). B. Time evolution of metastable-state probabilities. Colors match the state assignments shown in panel A. Shaded regions denote 95% confidence intervals, estimated using bootstrap resampling (10^5 iterations). C. Representative structures of each metastable state predicted from simulations. *Q* and *G* values at the cluster center, as well as the type of entanglement change (gain, loss or no change), are shown below each structure image. Color scheme follows that used in Fig. 2B, 2D, and 2F.

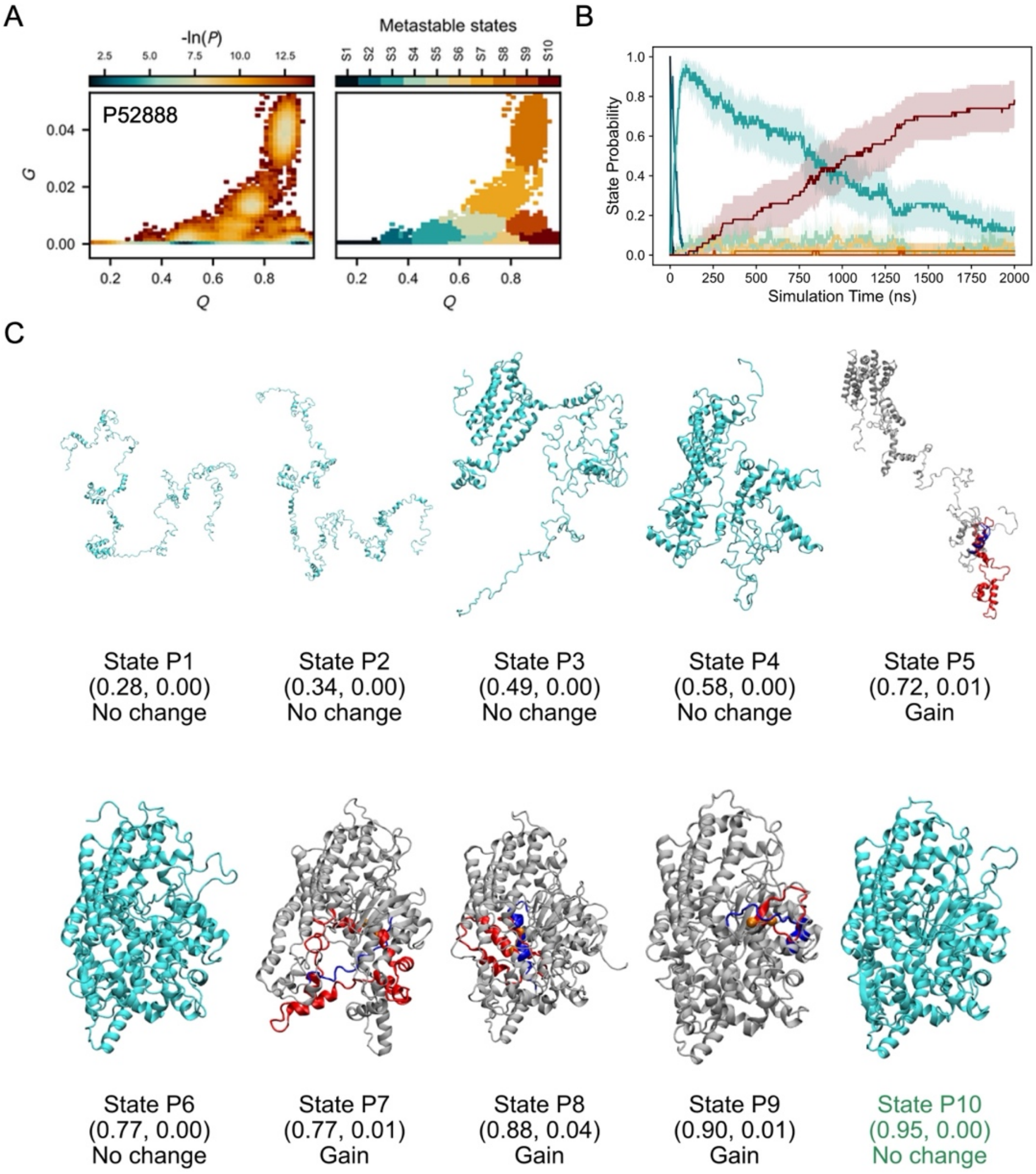

Figure S10. Simulation structural ensemble of the YU-E protein P52888. A. The probability distribution (-ln*P*) in the *Q* – *G* space (left) and the corresponding metastable states mapped onto structures from temperature-quench refolding simulations (right). B. Time evolution of metastable-state probabilities. Colors match the state assignments shown in panel A. Shaded regions denote 95% confidence intervals, estimated using bootstrap resampling (10^5 iterations). C. Representative structures of each metastable state predicted from simulations. *Q* and *G* values at the cluster center, as well as the type of entanglement change (gain, loss or no change), are shown below each structure image. Color scheme follows that used in Fig. 2B, 2D, and 2F.

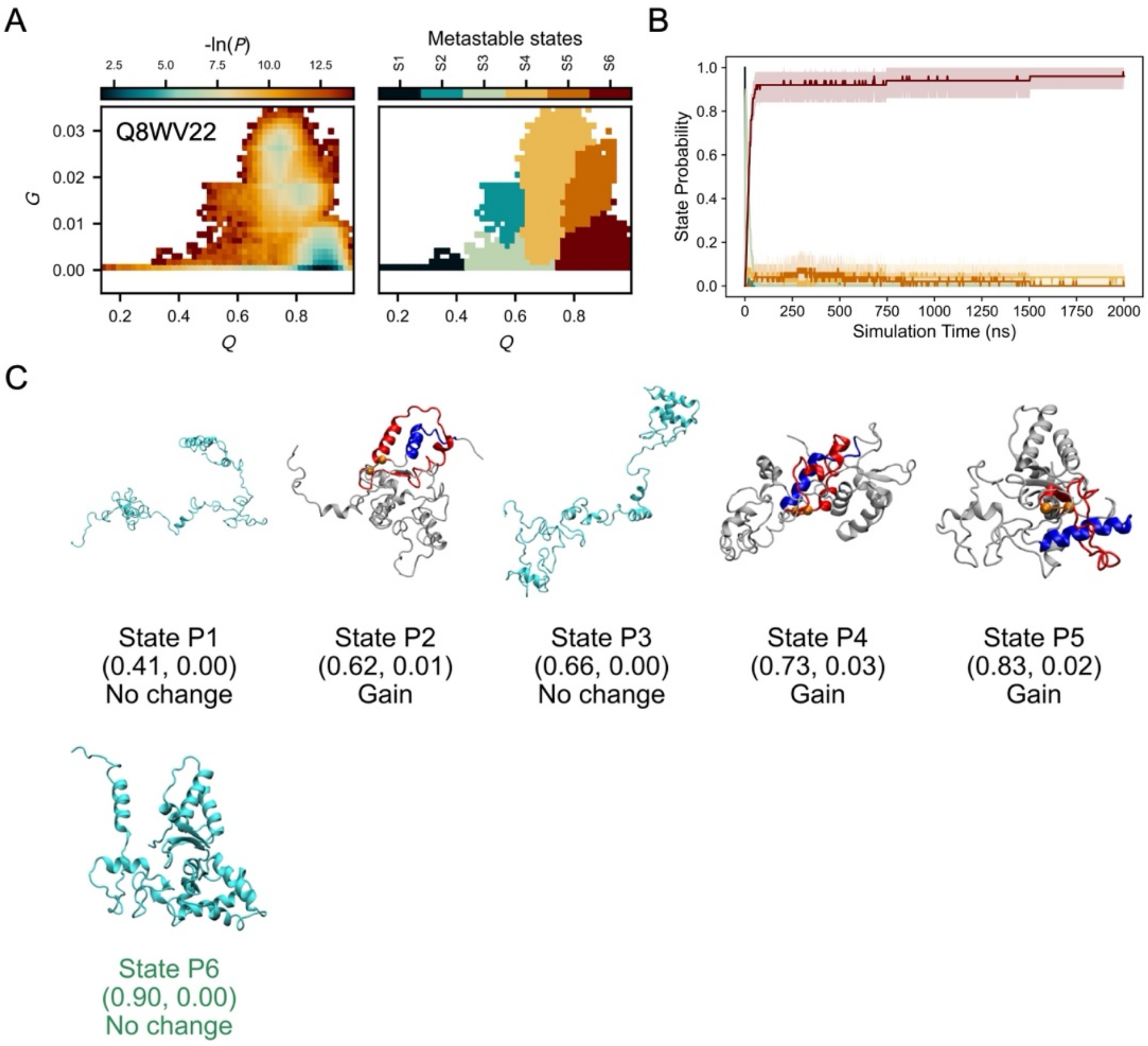

Figure S11. Simulation structural ensemble of the NU-NE protein Q8WV22. A. The probability distribution (-ln*P*) in the *Q* – *G* space (left) and the corresponding metastable states mapped onto structures from temperature-quench refolding simulations (right). B. Time evolution of metastable-state probabilities. Colors match the state assignments shown in panel A. Shaded regions denote 95% confidence intervals, estimated using bootstrap resampling (10^5 iterations). C. Representative structures of each metastable state predicted from simulations. *Q* and *G* values at the cluster center, as well as the type of entanglement change (gain, loss or no change), are shown below each structure image. Color scheme follows that used in Fig. 2B, 2D, and 2F.

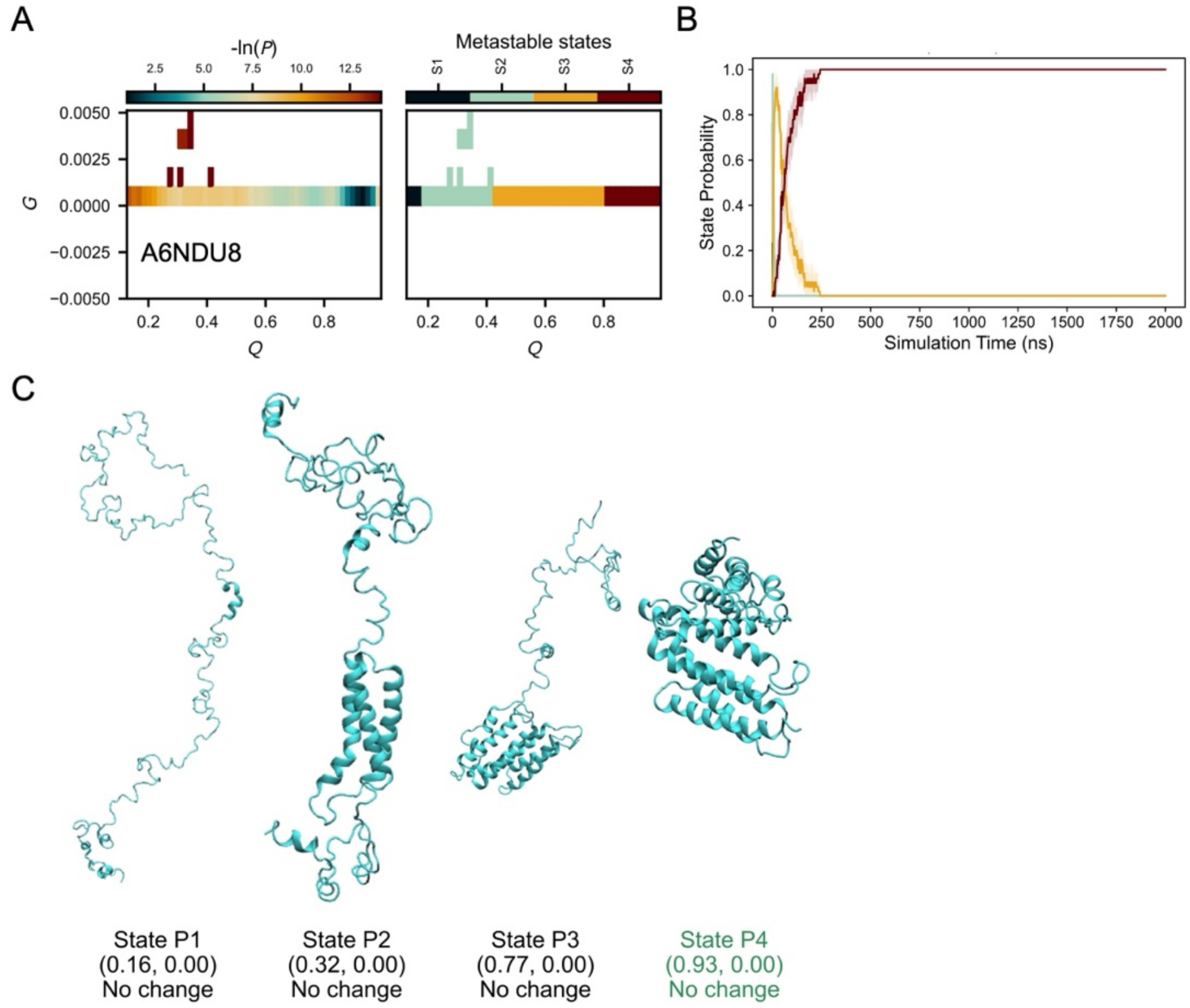

Figure S12. Simulation structural ensemble of the NU-NE protein A6NDU8. A. The probability distribution (-ln*P*) in the *Q* – *G* space (left) and the corresponding metastable states mapped onto structures from temperature-quench refolding simulations (right). B. Time evolution of metastable-state probabilities. Colors match the state assignments shown in panel A. Shaded regions denote 95% confidence intervals, estimated using bootstrap resampling (10^5 iterations). C. Representative structures of each metastable state predicted from simulations. *Q* and *G* values at the cluster center, as well as the type of entanglement change (gain, loss or no change), are shown below each structure image. Color scheme follows that used in Fig. 2B, 2D, and 2F.

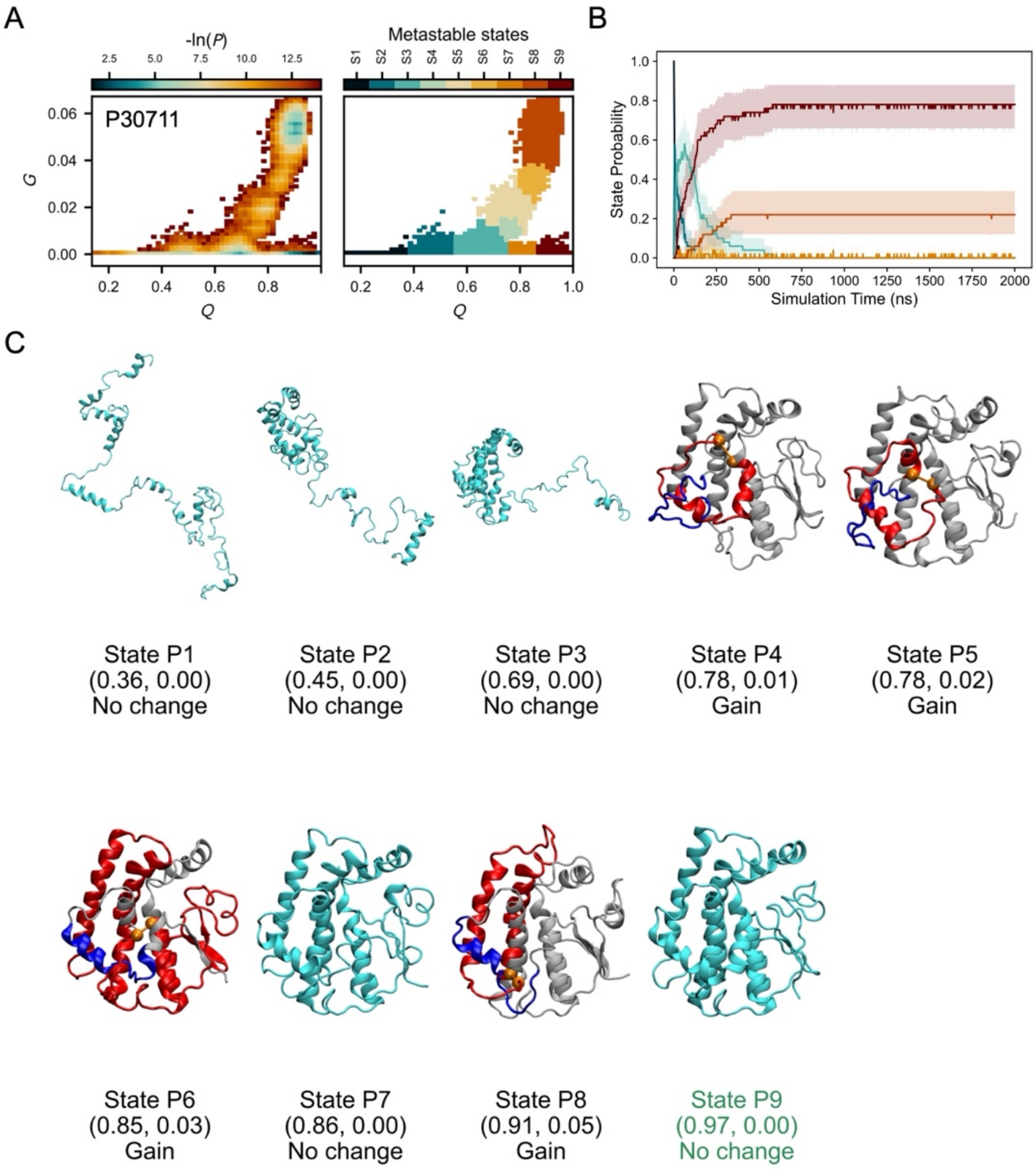

Figure S13. Simulation structural ensemble of the NU-NE protein P30711. A. The probability distribution (-ln*P*) in the *Q* – *G* space (left) and the corresponding metastable states mapped onto structures from temperature-quench refolding simulations (right). B. Time evolution of metastable-state probabilities. Colors match the state assignments shown in panel A. Shaded regions denote 95% confidence intervals, estimated using bootstrap resampling (10^5 iterations). C. Representative structures of each metastable state predicted from simulations. *Q* and *G* values at the cluster center, as well as the type of entanglement change (gain, loss or no change), are shown below each structure image. Color scheme follows that used in Fig. 2B, 2D, and 2F.

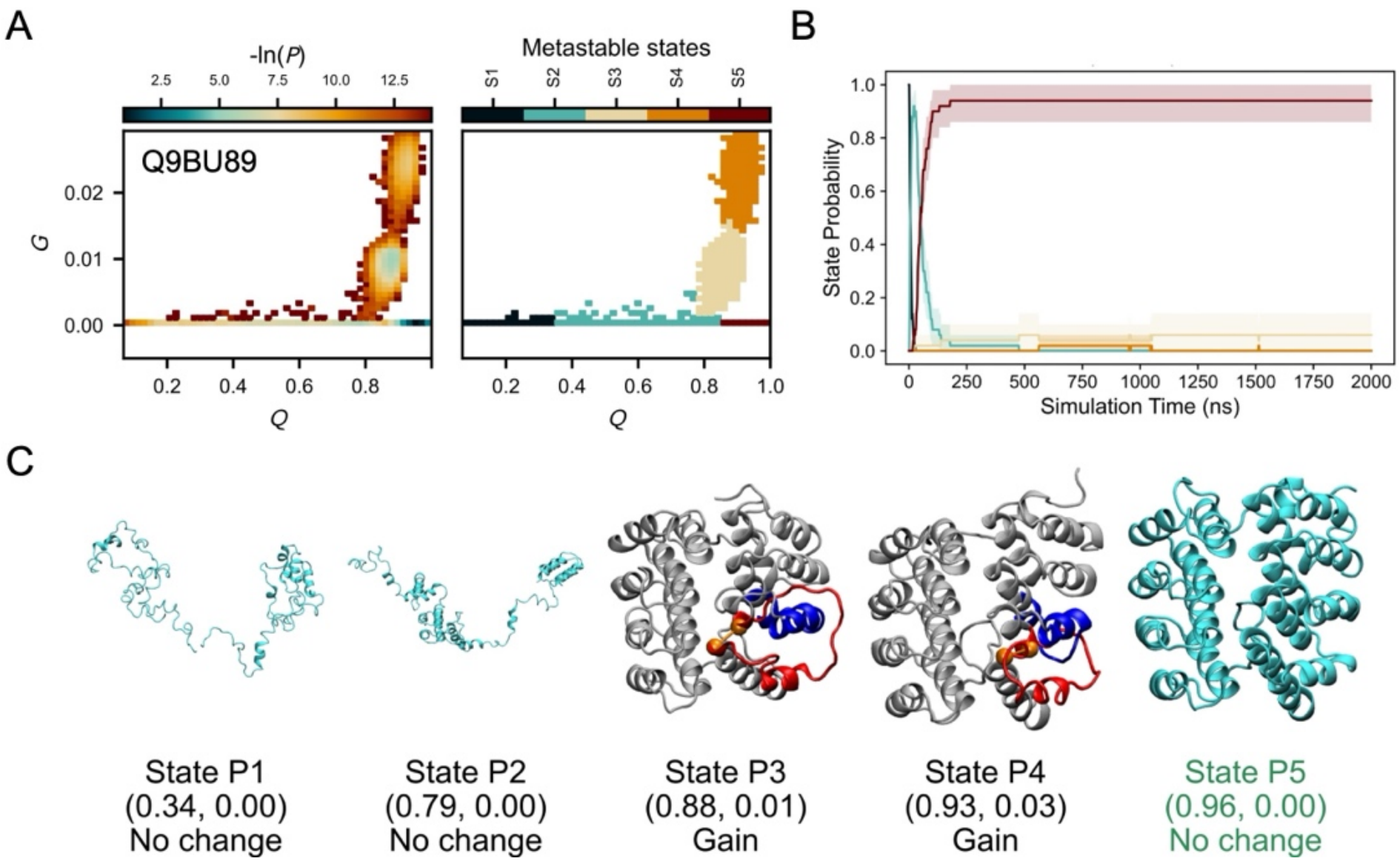

Figure S14. Simulation structural ensemble of the NU-NE protein Q9BU89. A. The probability distribution (-ln*P*) in the *Q* – *G* space (left) and the corresponding metastable states mapped onto structures from temperature-quench refolding simulations (right). B. Time evolution of metastable-state probabilities. Colors match the state assignments shown in panel A. Shaded regions denote 95% confidence intervals, estimated using bootstrap resampling (10^5 iterations). C. Representative structures of each metastable state predicted from simulations. *Q* and *G* values at the cluster center, as well as the type of entanglement change (gain, loss or no change), are shown below each structure image. Color scheme follows that used in Fig. 2B, 2D, and 2F.

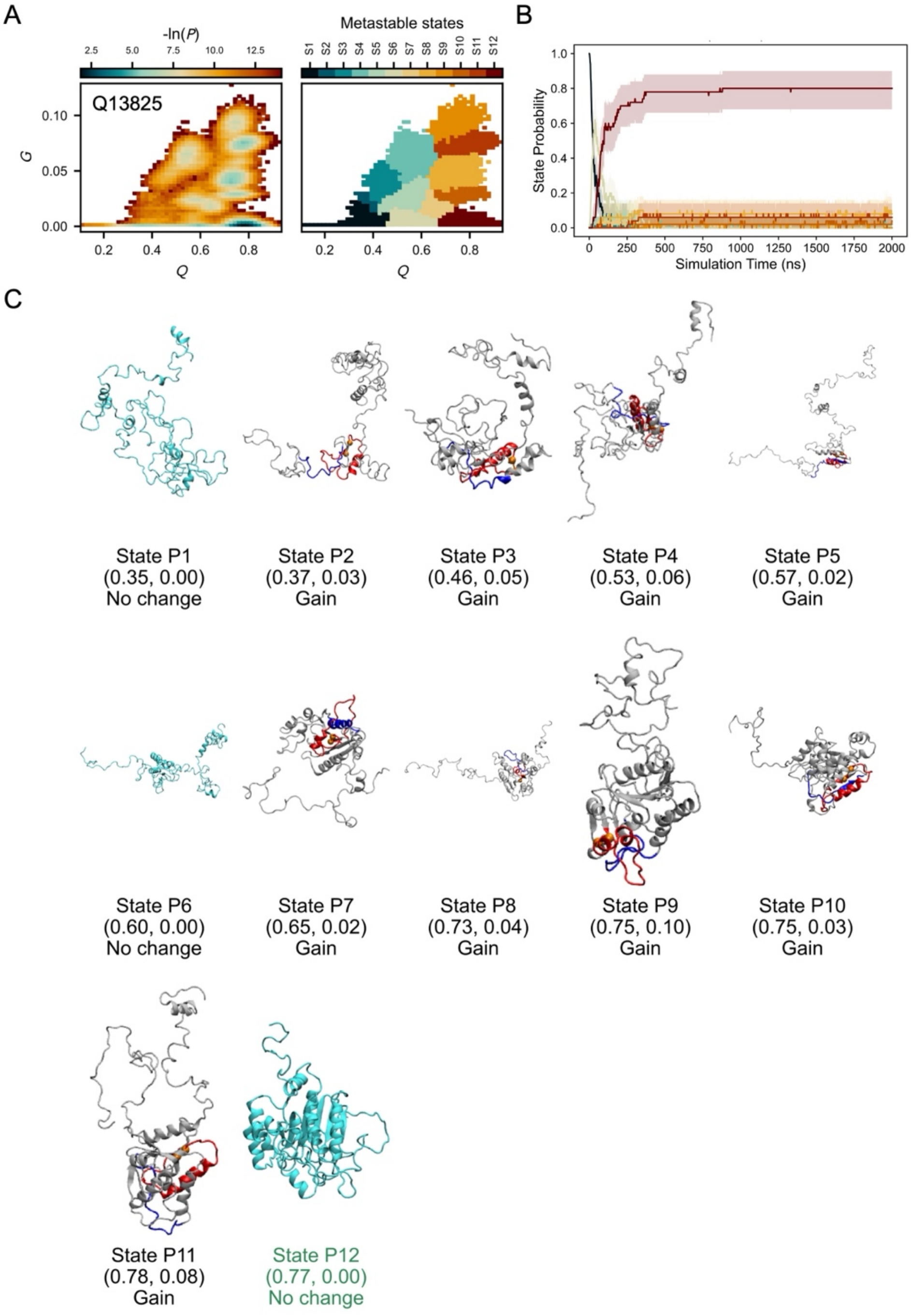

Figure S15. Simulation structural ensemble of the NU-NE protein Q13825. A. The probability distribution (-ln*P*) in the *Q* – *G* space (left) and the corresponding metastable states mapped onto structures from temperature-quench refolding simulations (right). B. Time evolution of metastable-state probabilities. Colors match the state assignments shown in panel A. Shaded regions denote 95% confidence intervals, estimated using bootstrap resampling (10^5 iterations). C. Representative structures of each metastable state predicted from simulations. *Q* and *G* values at the cluster center, as well as the type of entanglement change (gain, loss or no change), are shown below each structure image. Color scheme follows that used in Fig. 2B, 2D, and 2F.

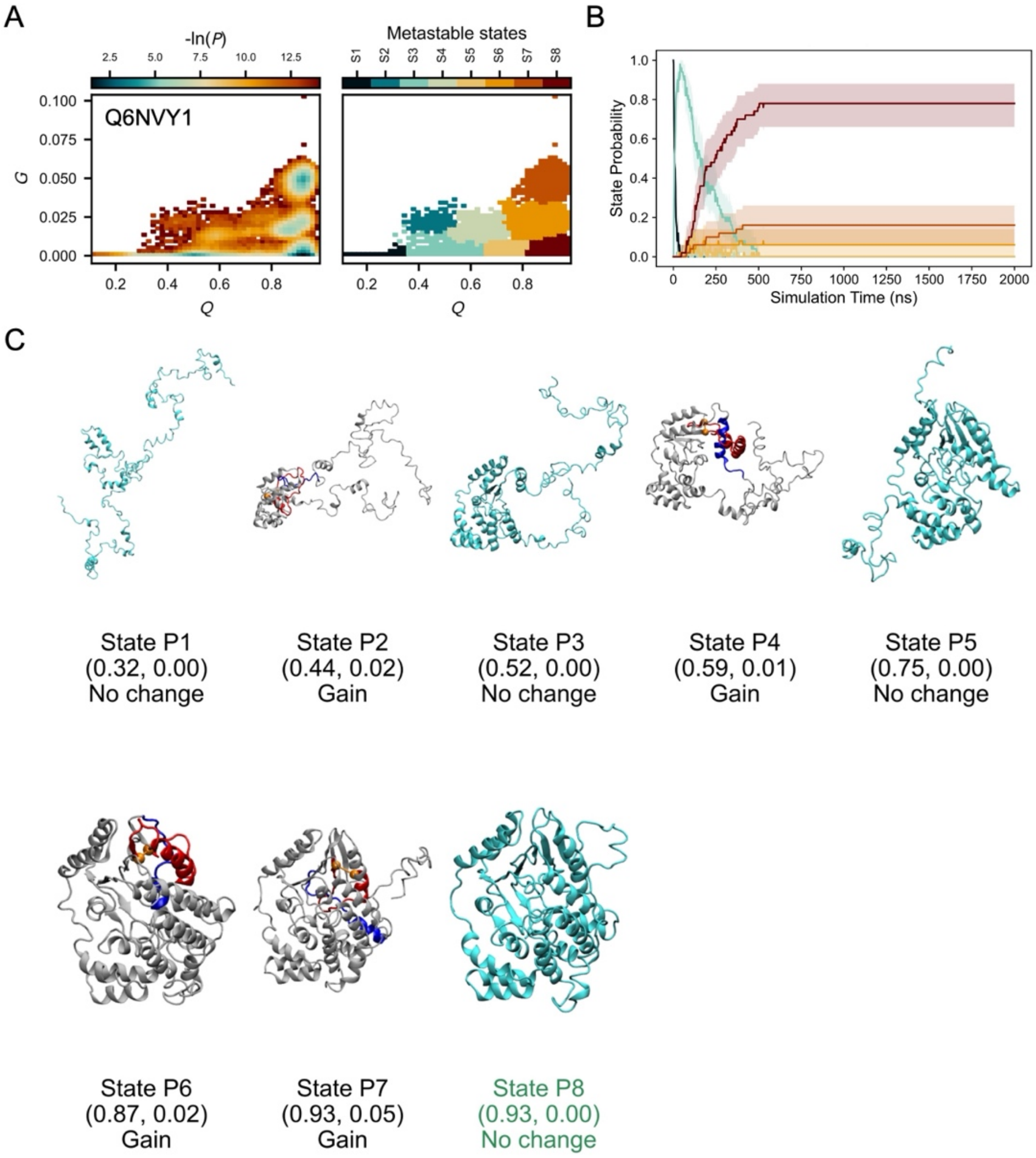

Figure S16. Simulation structural ensemble of the NU-NE protein Q6NVY1. A. The probability distribution (-ln*P*) in the *Q* – *G* space (left) and the corresponding metastable states mapped onto structures from temperature-quench refolding simulations (right). B. Time evolution of metastable-state probabilities. Colors match the state assignments shown in panel A. Shaded regions denote 95% confidence intervals, estimated using bootstrap resampling (10^5 iterations). C. Representative structures of each metastable state predicted from simulations. *Q* and *G* values at the cluster center, as well as the type of entanglement change (gain, loss or no change), are shown below each structure image. Color scheme follows that used in Fig. 2B, 2D, and 2F.

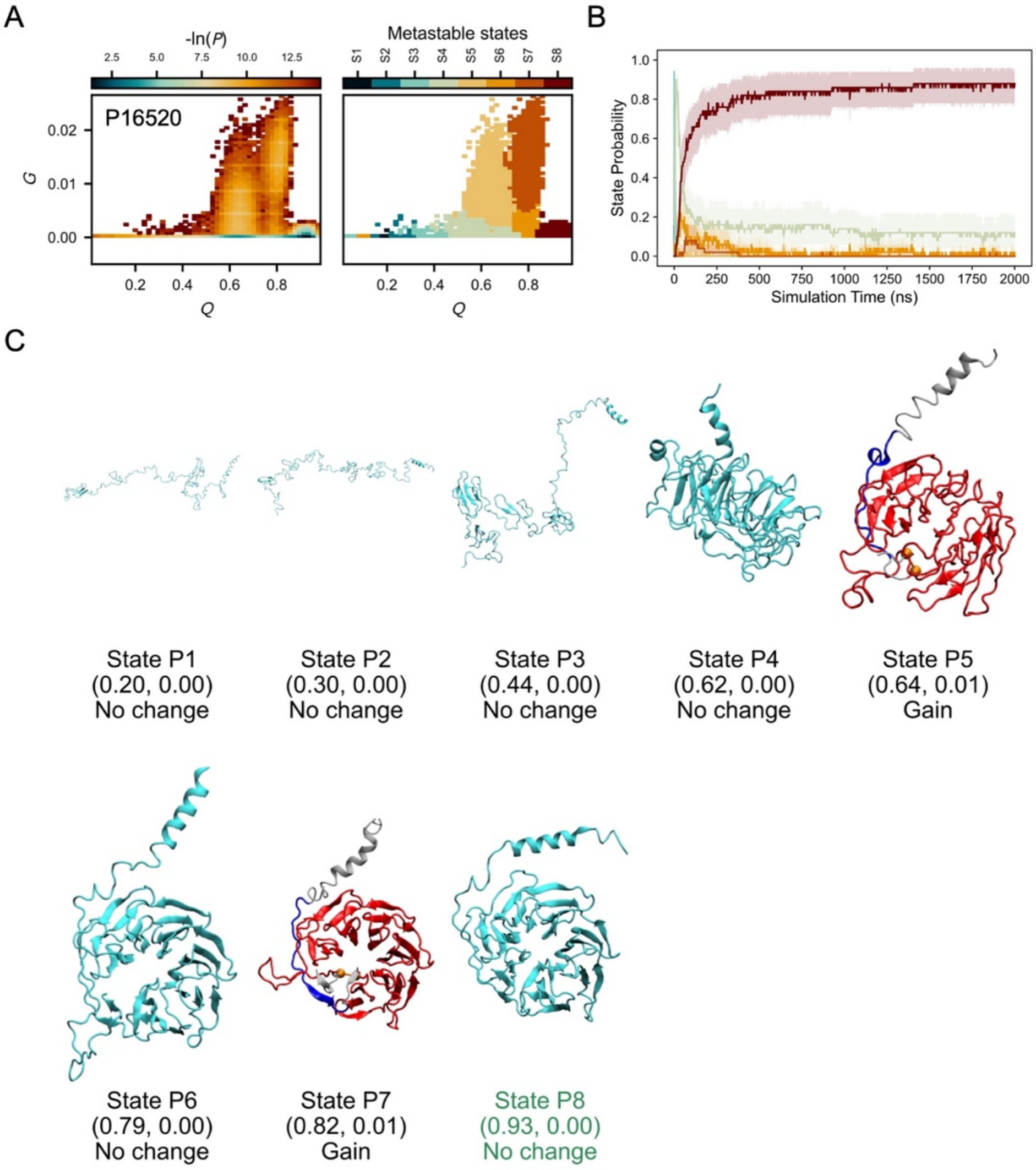

Figure S17. Simulation structural ensemble of the NU-NE protein P16520. A. The probability distribution (-ln*P*) in the *Q* – *G* space (left) and the corresponding metastable states mapped onto structures from temperature-quench refolding simulations (right). B. Time evolution of metastable-state probabilities. Colors match the state assignments shown in panel A. Shaded regions denote 95% confidence intervals, estimated using bootstrap resampling (10^5 iterations). C. Representative structures of each metastable state predicted from simulations. *Q* and *G* values at the cluster center, as well as the type of entanglement change (gain, loss or no change), are shown below each structure image. Color scheme follows that used in Fig. 2B, 2D, and 2F.

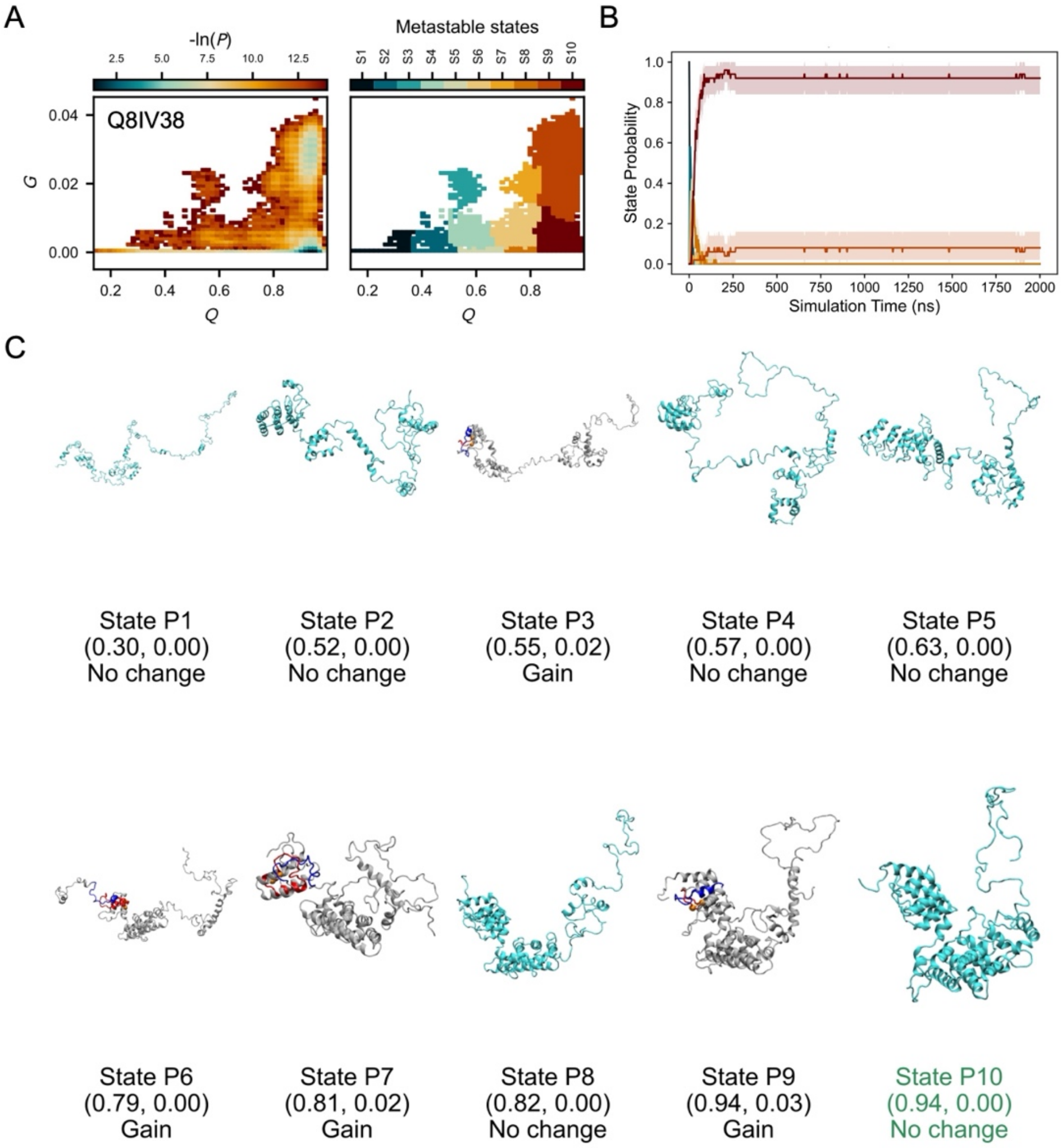

Figure S18. Simulation structural ensemble of the NU-NE protein Q8IV38. A. The probability distribution (-ln$P$) in the Q – G space (left) and the corresponding metastable states mapped onto structures from temperature-quench refolding simulations (right). B. Time evolution of metastable-state probabilities. Colors match the state assignments shown in panel A. Shaded regions denote 95% confidence intervals, estimated using bootstrap resampling (10^5 iterations). C. Representative structures of each metastable state predicted from simulations. Q and G values at the cluster center, as well as the type of entanglement change (gain, loss or no change), are shown below each structure image. Color scheme follows that used in Fig. 2B, 2D, and 2F.

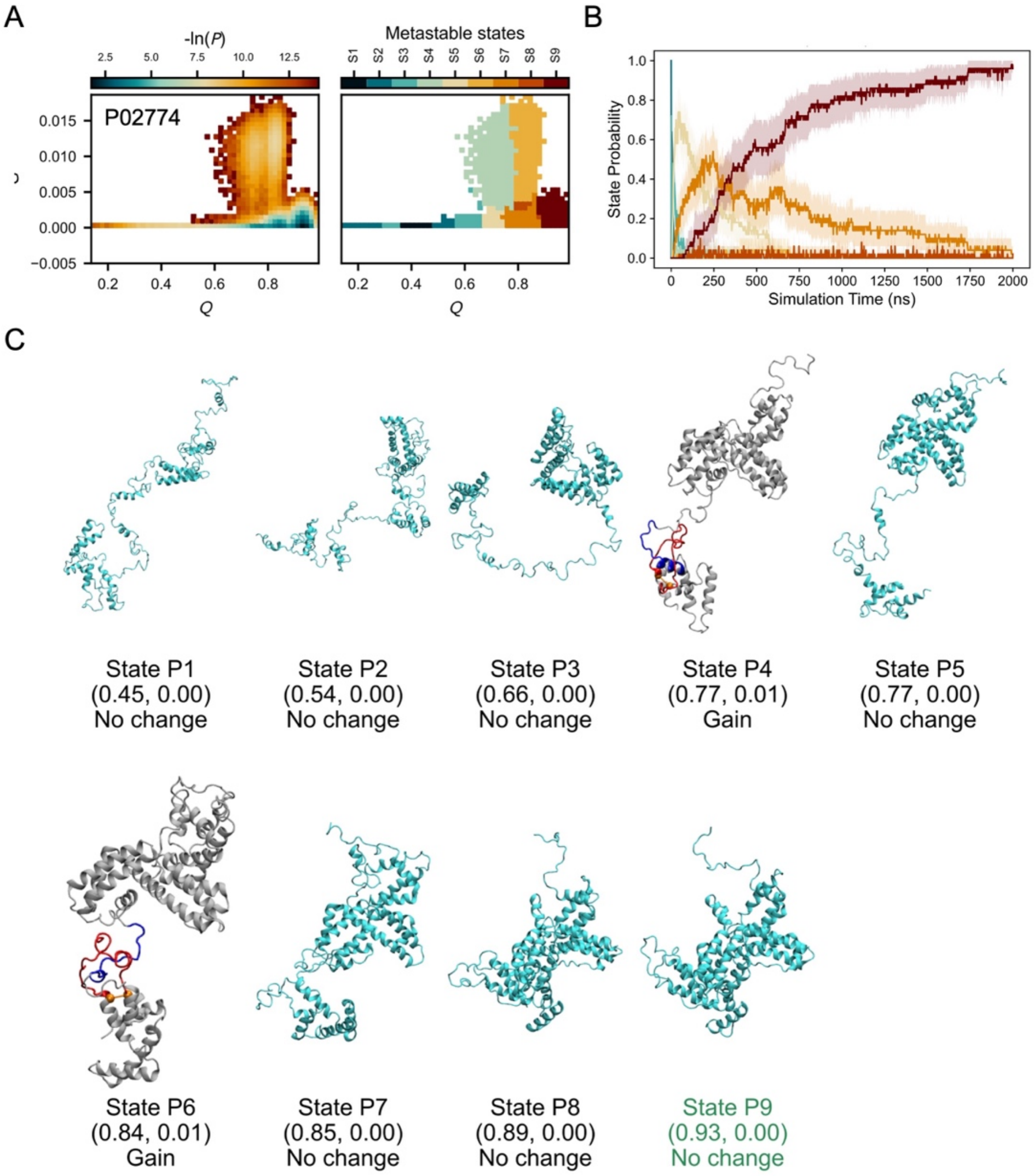

Figure S19. Simulation structural ensemble of the NU-NE protein P02774. A. The probability distribution (-ln*P*) in the *Q* – *G* space (left) and the corresponding metastable states mapped onto structures from temperature-quench refolding simulations (right). B. Time evolution of metastable-state probabilities. Colors match the state assignments shown in panel A. Shaded regions denote 95% confidence intervals, estimated using bootstrap resampling (10^5 iterations). C. Representative structures of each metastable state predicted from simulations. *Q* and *G* values at the cluster center, as well as the type of entanglement change (gain, loss or no change), are shown below each structure image. Color scheme follows that used in Fig. 2B, 2D, and 2F.

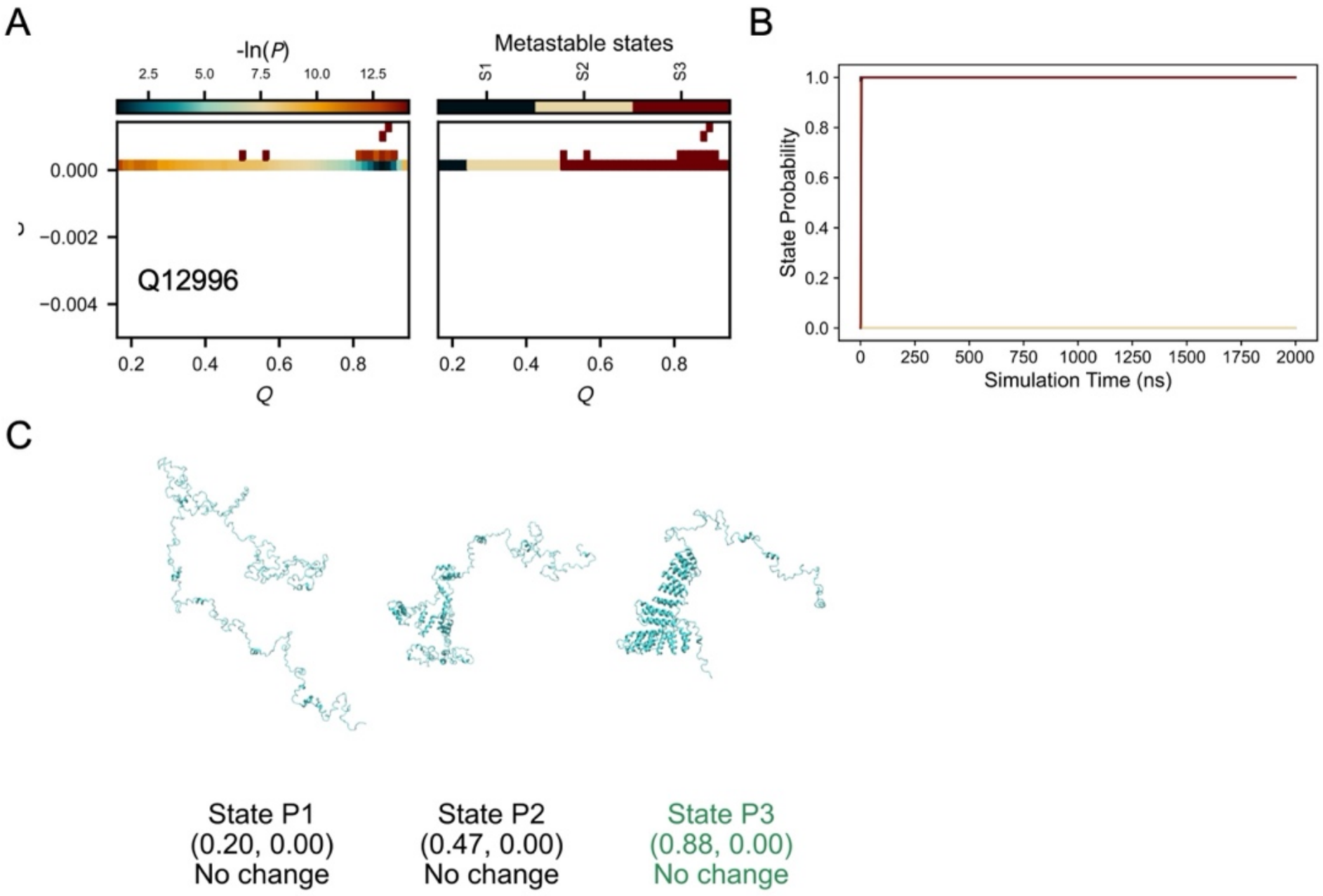

Figure S20. Simulation structural ensemble of the NU-NE protein Q12996. A. The probability distribution (-ln*P*) in the *Q* – *G* space (left) and the corresponding metastable states mapped onto structures from temperature-quench refolding simulations (right). B. Time evolution of metastable-state probabilities. Colors match the state assignments shown in panel A. Shaded regions denote 95% confidence intervals, estimated using bootstrap resampling (10^5 iterations). C. Representative structures of each metastable state predicted from simulations. *Q* and *G* values at the cluster center, as well as the type of entanglement change (gain, loss or no change), are shown below each structure image. Color scheme follows that used in Fig. 2B, 2D, and 2F.

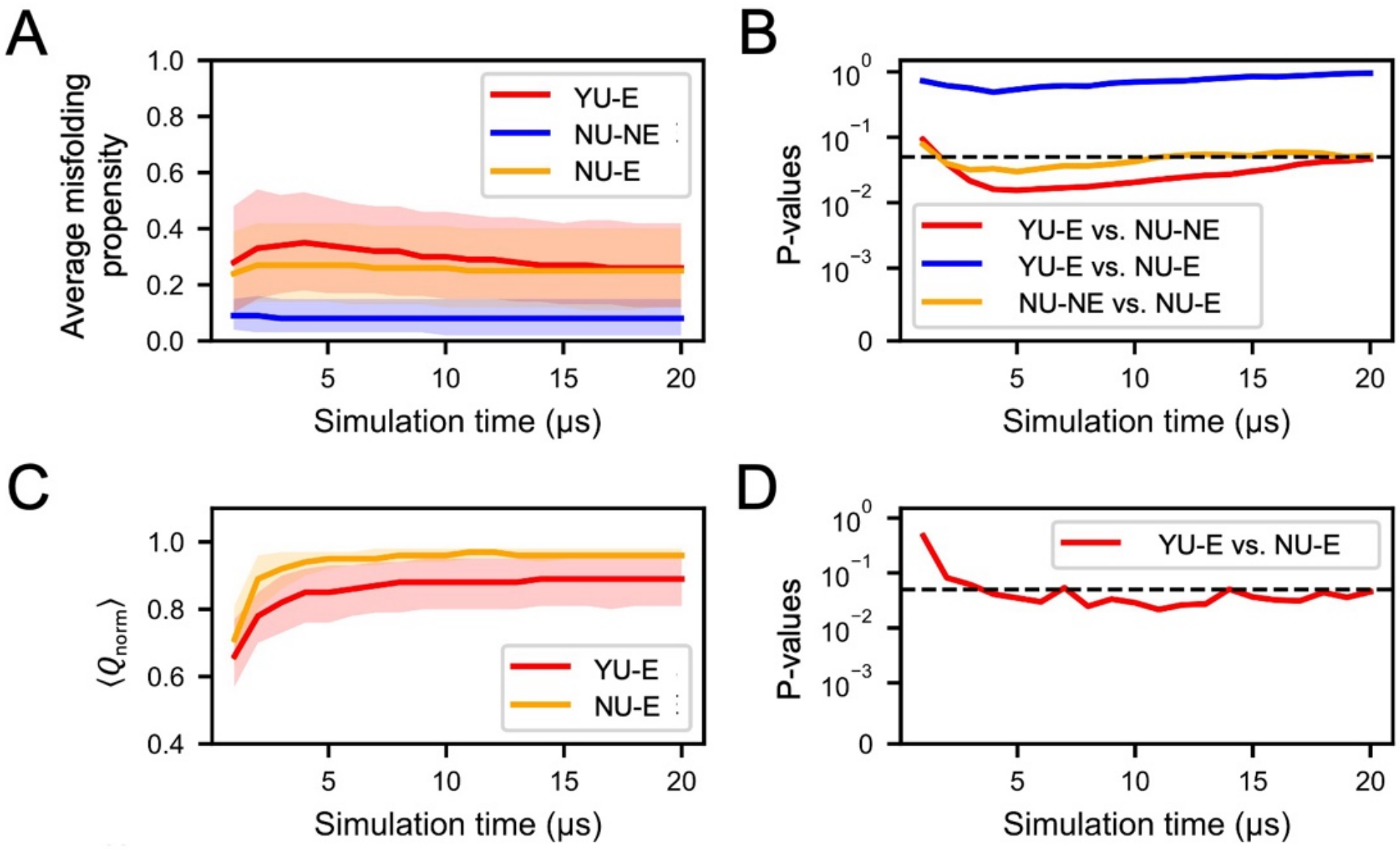

Figure S21. Average misfolding propensity and $\langle Q_{\mathrm{norm}} \rangle$ along the extended simulation trajectories. A. Average misfolding propensity of YU-E (red), NU-NE (blue) and NU-E (orange) proteins. Shaded regions denote 95% confidence intervals, estimated using hierarchical bootstrap resampling (10^5 iterations). B. P-values for the differences in misfolding propensities between YU-E and NU-NE (red), YU-E and NU-E (blue) and NU-NE and NU-E (orange) proteins, estimated using two-sided hierarchical permutation test for 10^6 iterations. The horizontal dashed line marks the significance level at 0.05. C. $\langle Q_{\mathrm{norm}} \rangle$ of YU-E (red) and NU-E (orange) proteins. Shaded regions denote 95% confidence intervals, estimated using hierarchical bootstrap resampling (10^5 iterations). D. P-values for the differences in $\langle Q_{\mathrm{norm}} \rangle$ between YU-E and NU-E (red) proteins, estimated using two-sided hierarchical permutation test for 10^6 iterations. The horizontal dashed line marks the significance level at 0.05.

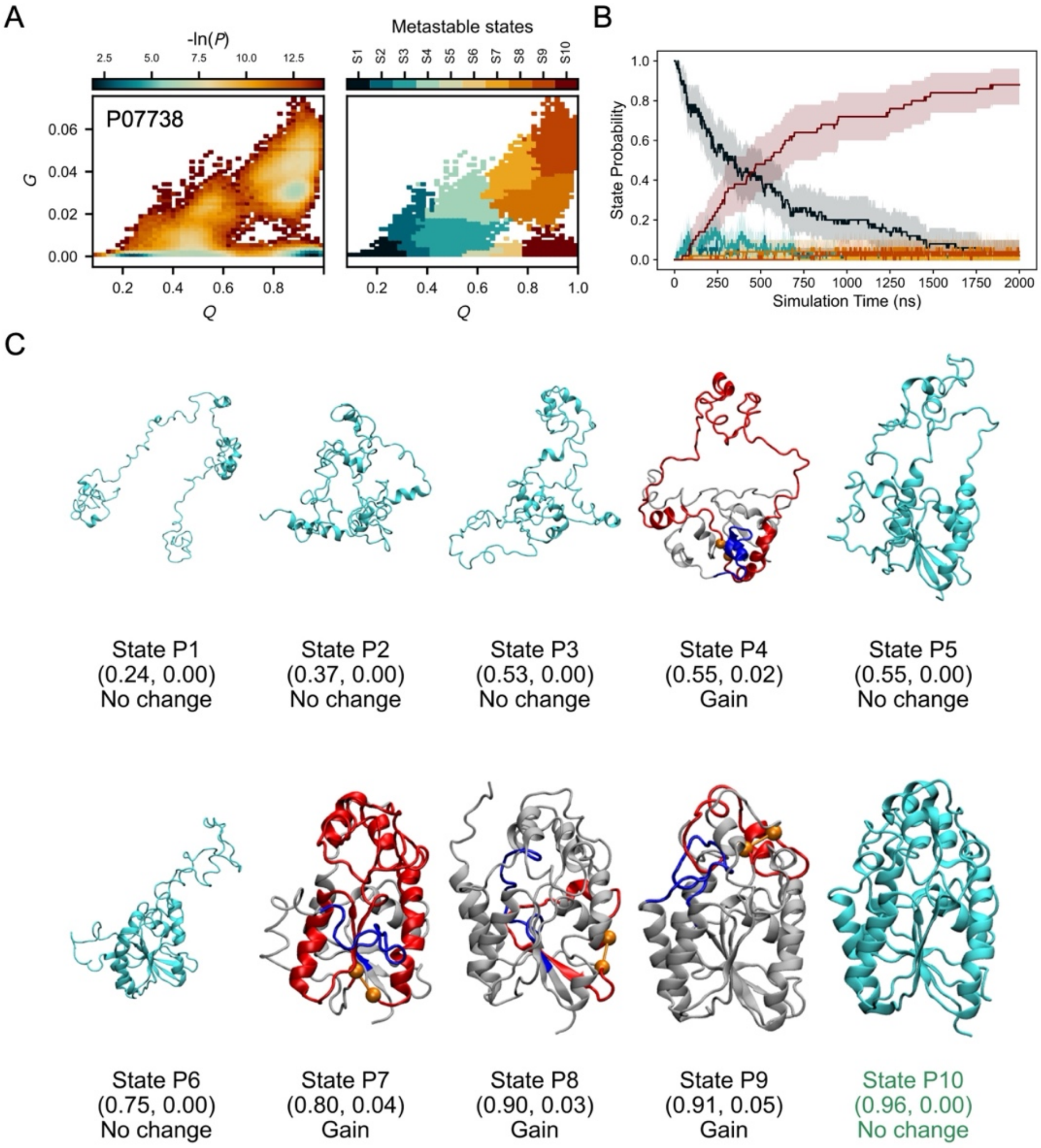

Figure S22. Simulation structural ensemble of the NU-E protein P07738. A. The probability distribution (-ln*P*) in the *Q* – *G* space (left) and the corresponding metastable states mapped onto structures from temperature-quench refolding simulations (right). B. Time evolution of metastable-state probabilities. Colors match the state assignments shown in panel A. Shaded regions denote 95% confidence intervals, estimated using bootstrap resampling (10^5 iterations). C. Representative structures of each metastable state predicted from simulations. *Q* and *G* values at the cluster center, as well as the type of entanglement change (gain, loss or no change), are shown below each structure image. Color scheme follows that used in Fig. 2B, 2D, and 2F.

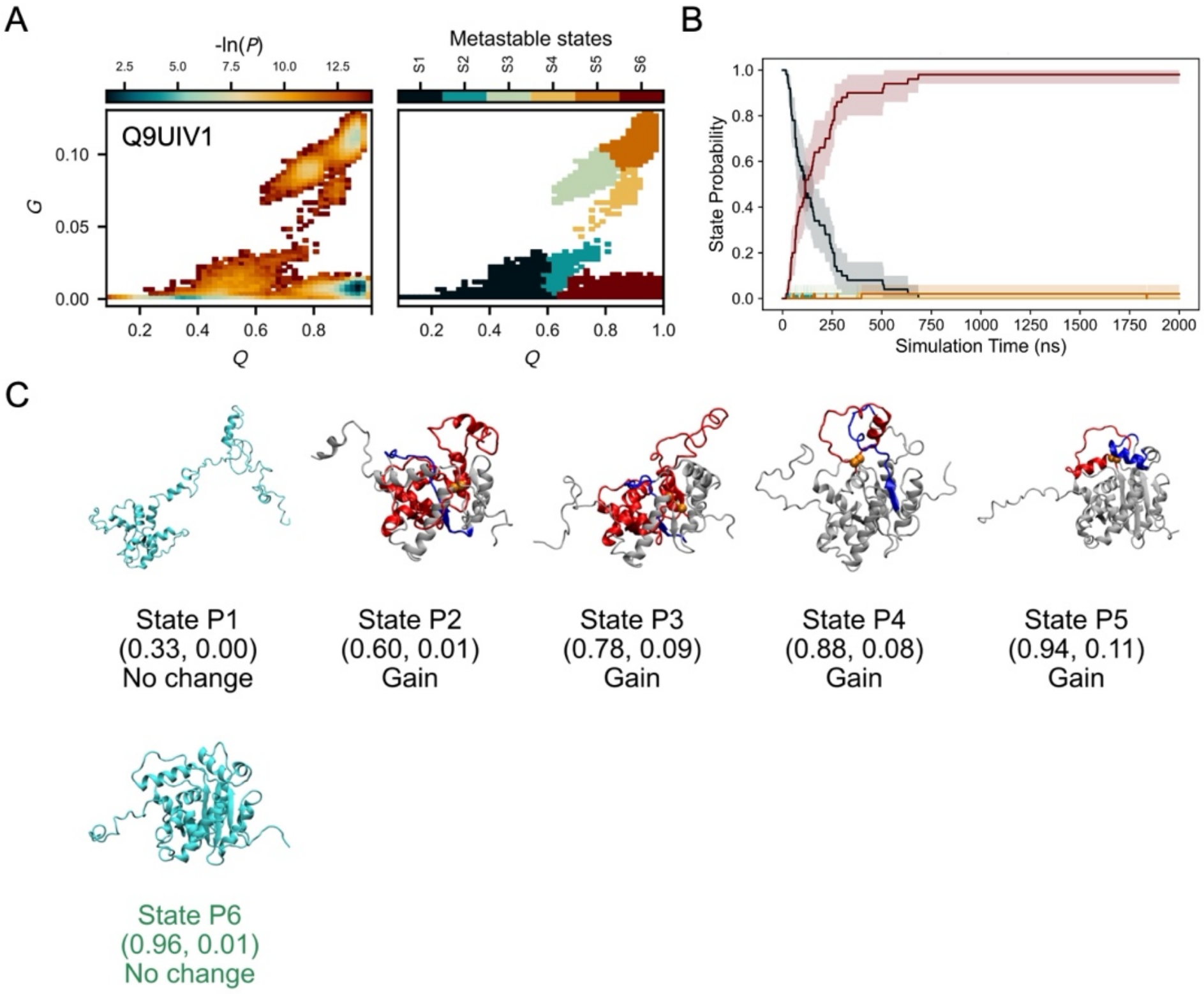

Figure S23. Simulation structural ensemble of the NU-E protein Q9UIV1. A. The probability distribution (-ln*P*) in the *Q* – *G* space (left) and the corresponding metastable states mapped onto structures from temperature-quench refolding simulations (right). B. Time evolution of metastable-state probabilities. Colors match the state assignments shown in panel A. Shaded regions denote 95% confidence intervals, estimated using bootstrap resampling (10^5 iterations). C. Representative structures of each metastable state predicted from simulations. *Q* and *G* values at the cluster center, as well as the type of entanglement change (gain, loss or no change), are shown below each structure image. Color scheme follows that used in Fig. 2B, 2D, and 2F.

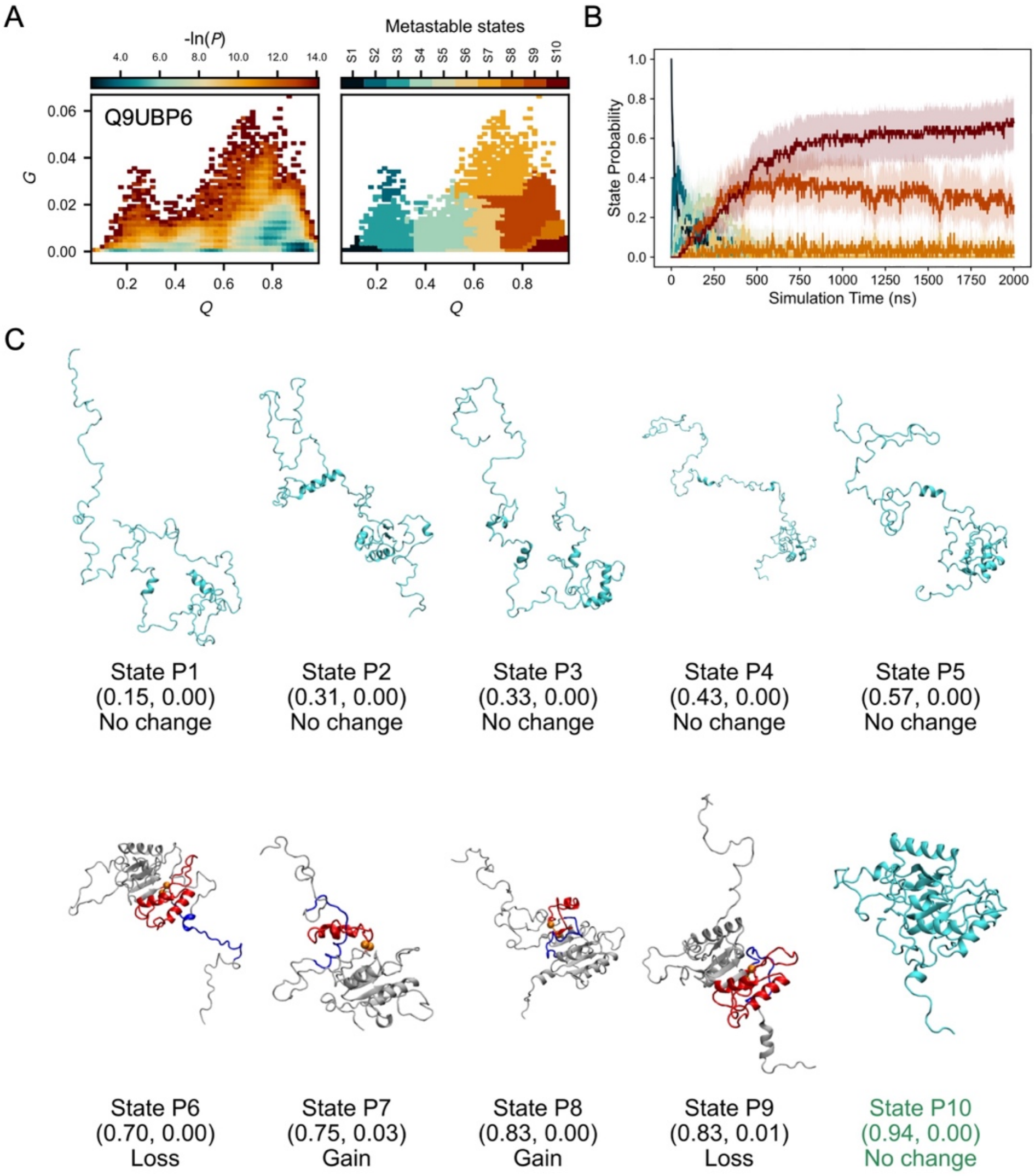

Figure S24. Simulation structural ensemble of the NU-E protein Q9UBP6. A. The probability distribution (-ln*P*) in the *Q* – *G* space (left) and the corresponding metastable states mapped onto structures from temperature-quench refolding simulations (right). B. Time evolution of metastable-state probabilities. Colors match the state assignments shown in panel A. Shaded regions denote 95% confidence intervals, estimated using bootstrap resampling (10^5 iterations). C. Representative structures of each metastable state predicted from simulations. *Q* and *G* values at the cluster center, as well as the type of entanglement change (gain, loss or no change), are shown below each structure image. Color scheme follows that used in Fig. 2B, 2D, and 2F.

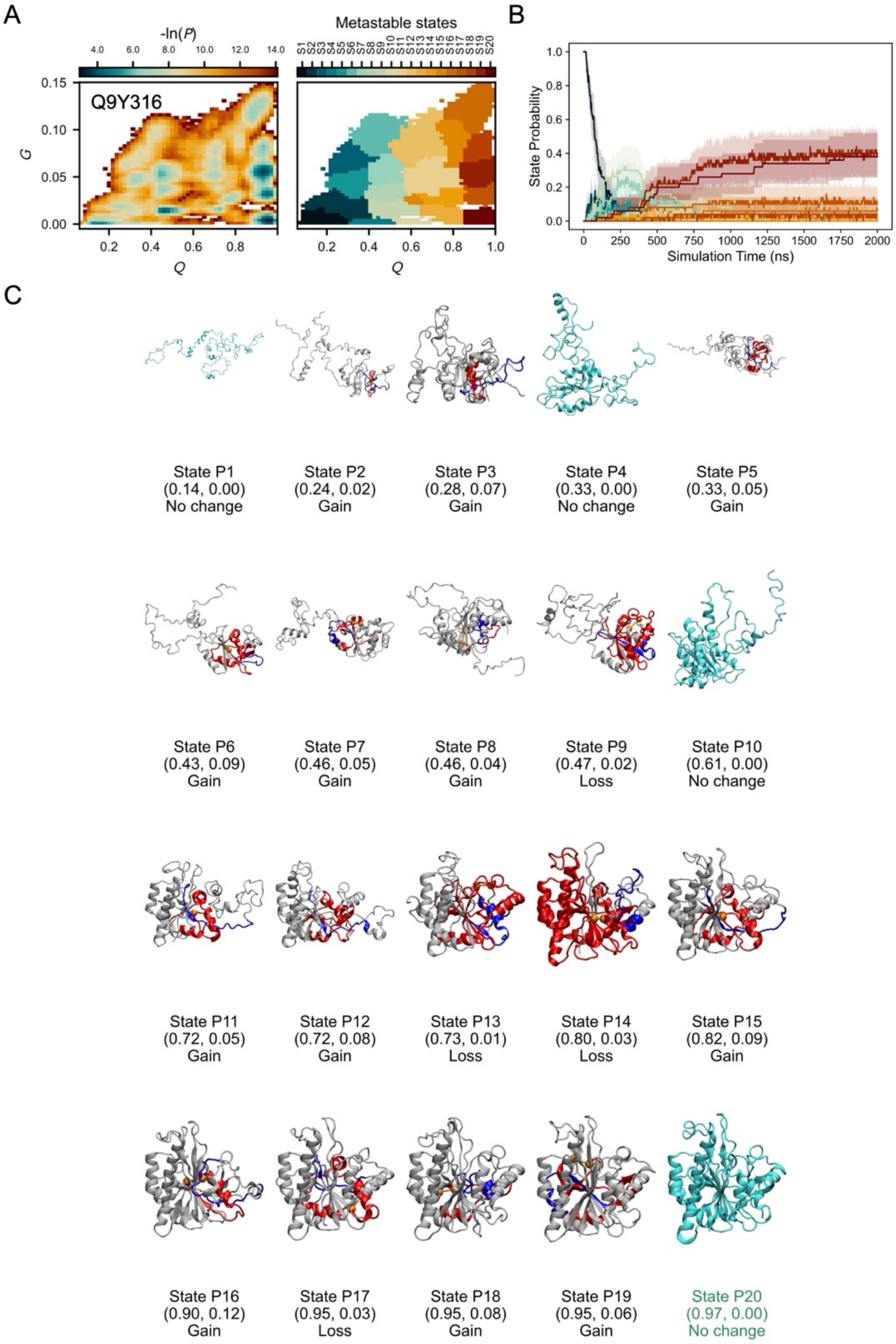

Figure S25. Simulation structural ensemble of the NU-E protein Q9Y316. A. The probability distribution (-ln*P*) in the *Q* – *G* space (left) and the corresponding metastable states mapped onto structures from temperature-quench refolding simulations (right). B. Time evolution of metastable-state probabilities. Colors match the state assignments shown in panel A. Shaded regions denote 95% confidence intervals, estimated using bootstrap resampling (10^5 iterations). C. Representative structures of each metastable state predicted from simulations. *Q* and *G* values at the cluster center, as well as the type of entanglement change (gain, loss or no change), are shown below each structure image. Color scheme follows that used in Fig. 2B, 2D, and 2F.

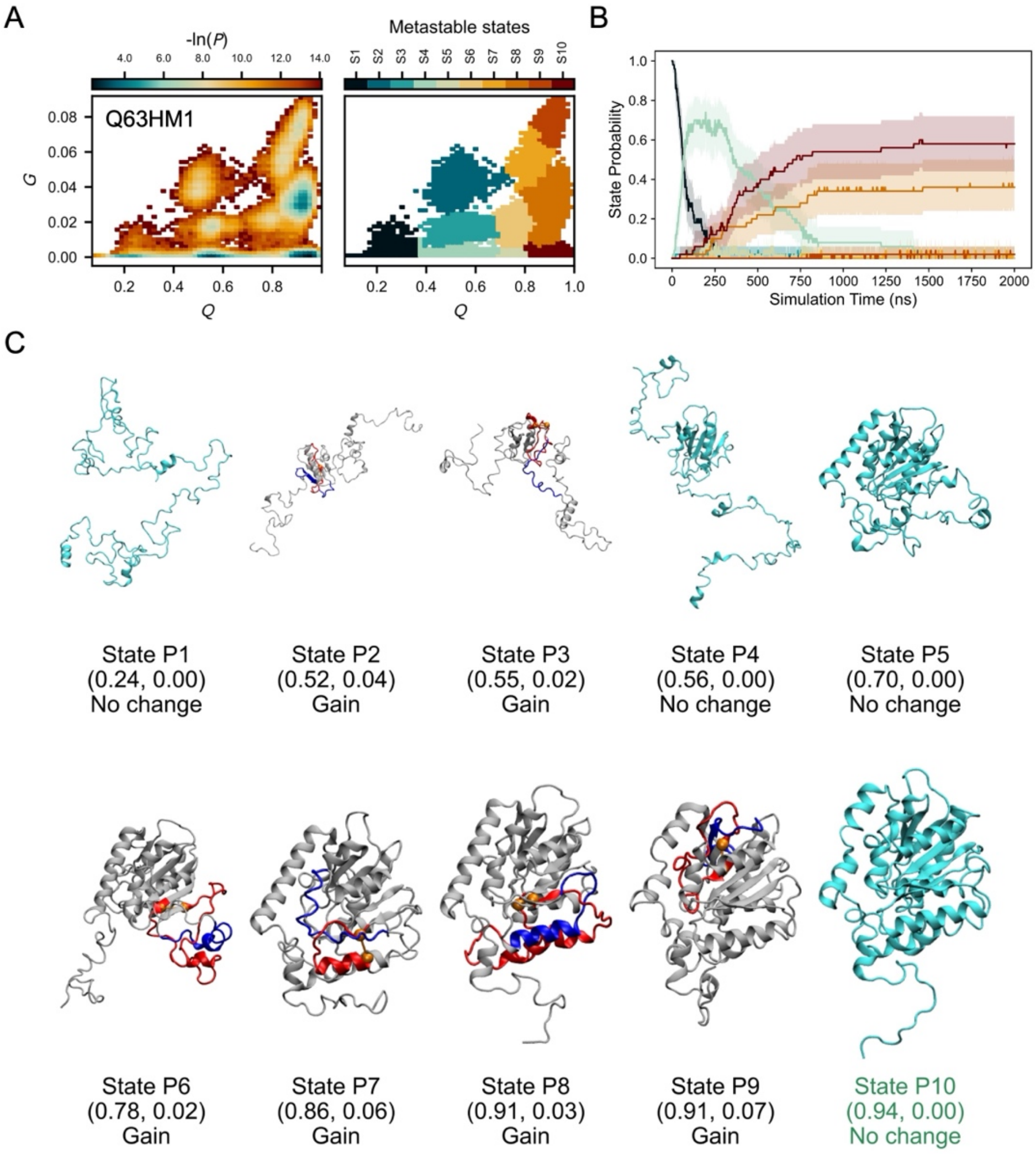

Figure S26. Simulation structural ensemble of the NU-E protein Q63HM1. A. The probability distribution (-ln*P*) in the *Q* – *G* space (left) and the corresponding metastable states mapped onto structures from temperature-quench refolding simulations (right). B. Time evolution of metastable-state probabilities. Colors match the state assignments shown in panel A. Shaded regions denote 95% confidence intervals, estimated using bootstrap resampling (10^5 iterations). C. Representative structures of each metastable state predicted from simulations. *Q* and *G* values at the cluster center, as well as the type of entanglement change (gain, loss or no change), are shown below each structure image. Color scheme follows that used in Fig. 2B, 2D, and 2F.

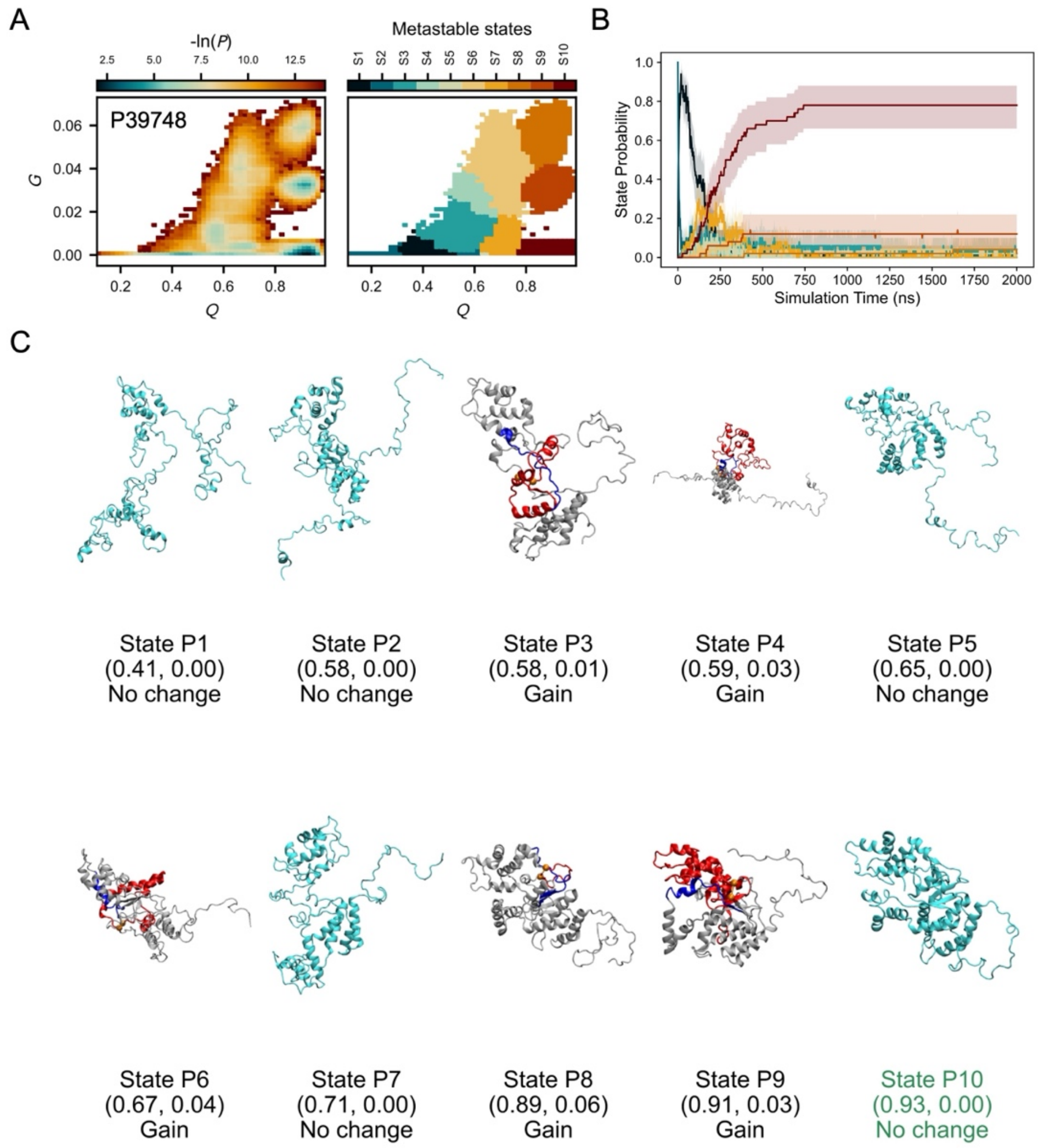

Figure S27. Simulation structural ensemble of the NU-E protein P39748. A. The probability distribution (-ln*P*) in the *Q* – *G* space (left) and the corresponding metastable states mapped onto structures from temperature-quench refolding simulations (right). B. Time evolution of metastable-state probabilities. Colors match the state assignments shown in panel A. Shaded regions denote 95% confidence intervals, estimated using bootstrap resampling (10^5 iterations). C. Representative structures of each metastable state predicted from simulations. *Q* and *G* values at the cluster center, as well as the type of entanglement change (gain, loss or no change), are shown below each structure image. Color scheme follows that used in Fig. 2B, 2D, and 2F.

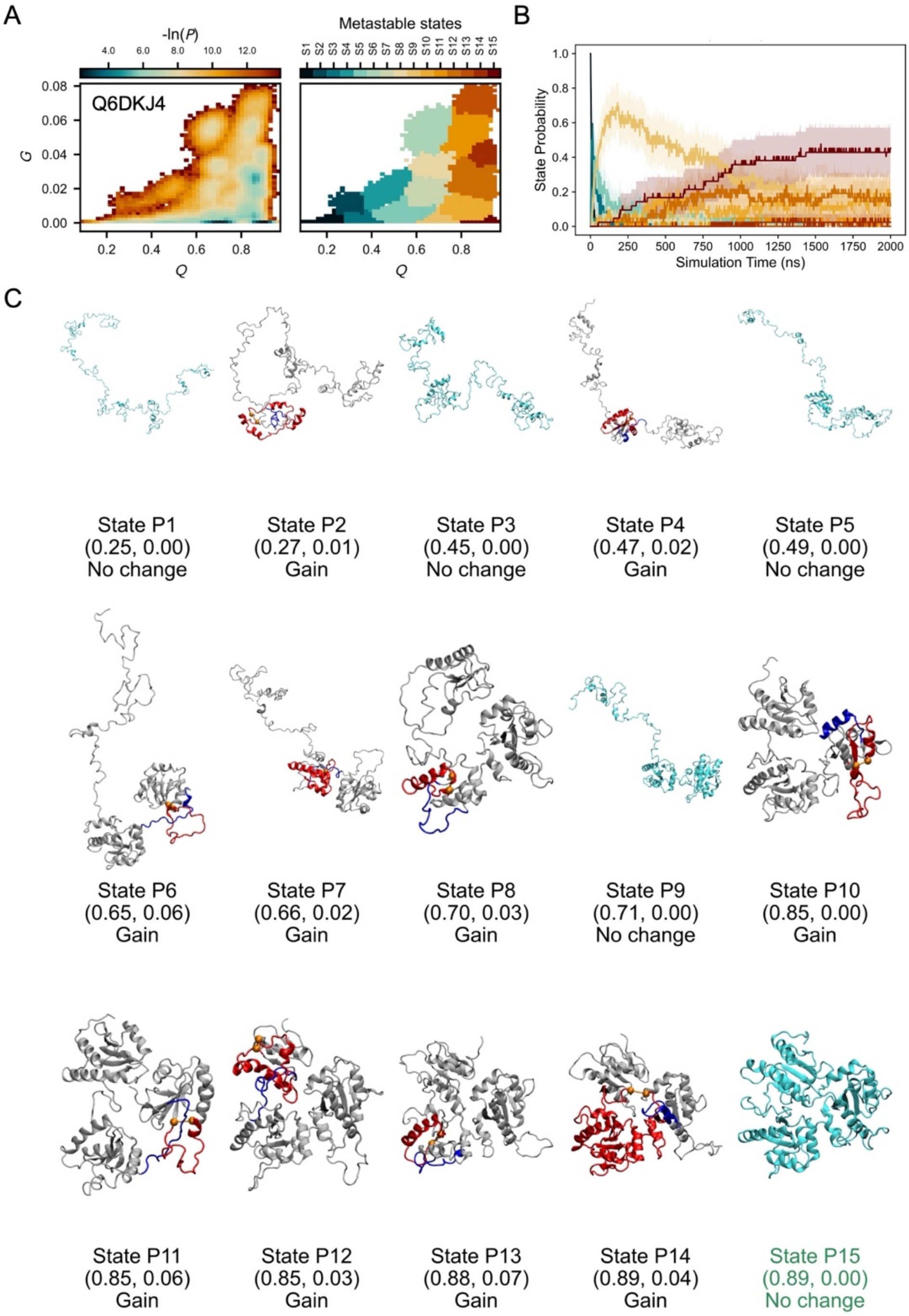

Figure S28. Simulation structural ensemble of the NU-E protein Q6DKJ4. A. The probability distribution (-ln*P*) in the *Q* – *G* space (left) and the corresponding metastable states mapped onto structures from temperature-quench refolding simulations (right). B. Time evolution of metastable-state probabilities. Colors match the state assignments shown in panel A. Shaded regions denote 95% confidence intervals, estimated using bootstrap resampling (10^5 iterations). C. Representative structures of each metastable state predicted from simulations. *Q* and *G* values at the cluster center, as well as the type of entanglement change (gain, loss or no change), are shown below each structure image. Color scheme follows that used in Fig. 2B, 2D, and 2F.

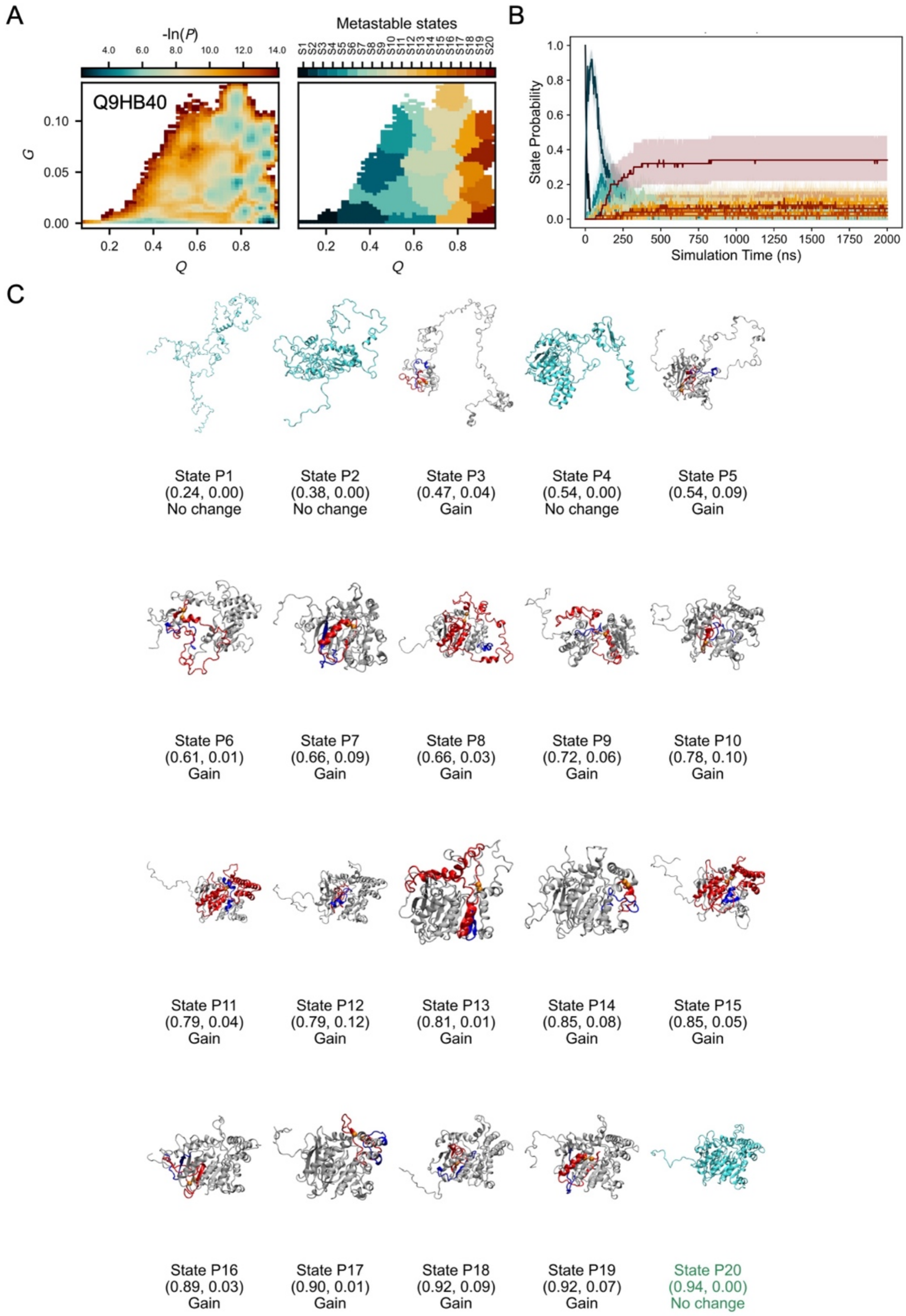

Figure S29. Simulation structural ensemble of the NU-E protein Q9HB40. A. The probability distribution (-ln*P*) in the *Q* – *G* space (left) and the corresponding metastable states mapped onto structures from temperature-quench refolding simulations (right). B. Time evolution of metastable-state probabilities. Colors match the state assignments shown in panel A. Shaded regions denote 95% confidence intervals, estimated using bootstrap resampling (10^5 iterations). C. Representative structures of each metastable state predicted from simulations. *Q* and *G* values at the cluster center, as well as the type of entanglement change (gain, loss or no change), are shown below each structure image. Color scheme follows that used in Fig. 2B, 2D, and 2F.

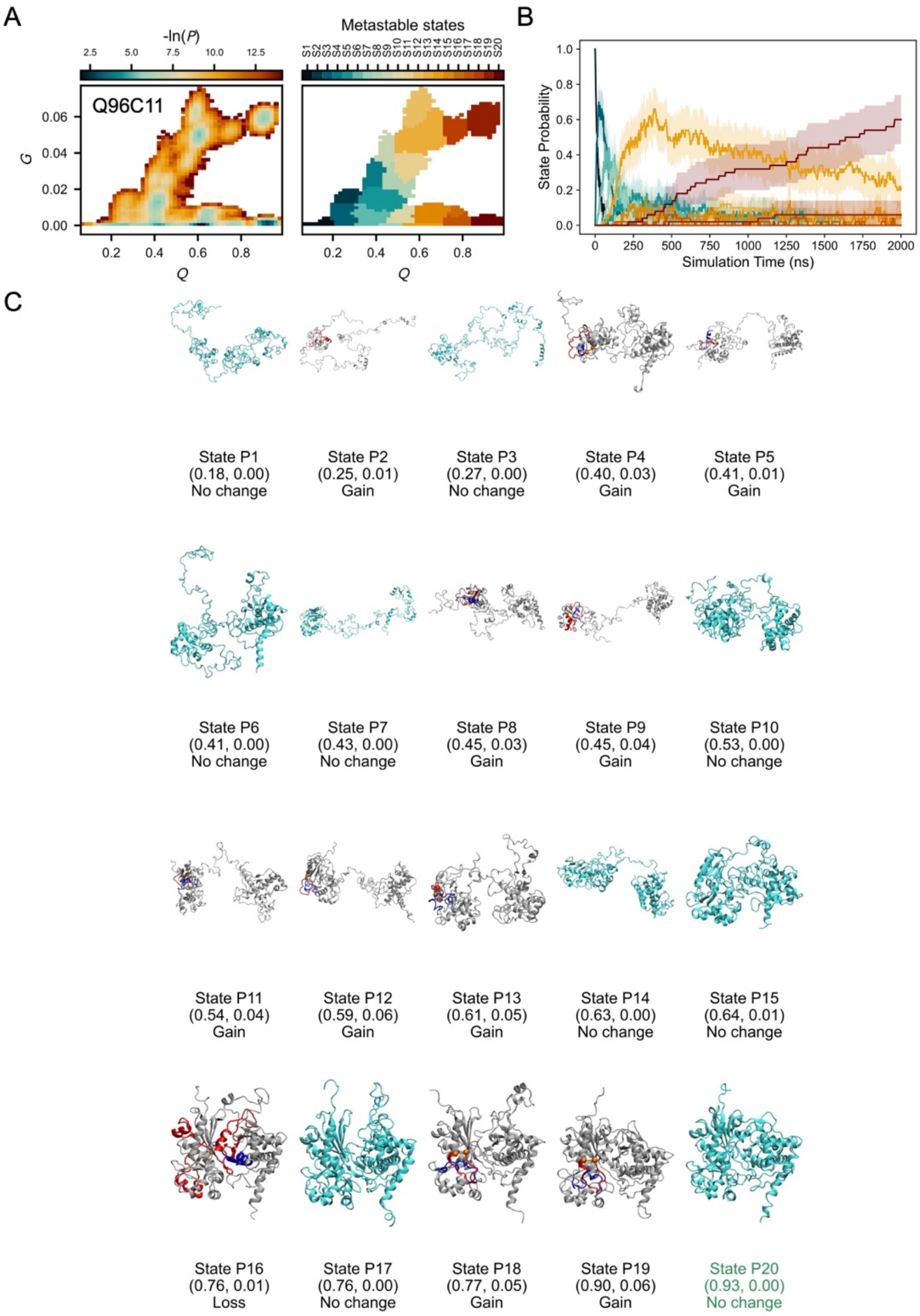

Figure S30. Simulation structural ensemble of the NU-E protein Q96C11. A. The probability distribution (-ln*P*) in the *Q* – *G* space (left) and the corresponding metastable states mapped onto structures from temperature-quench refolding simulations (right). B. Time evolution of metastable-state probabilities. Colors match the state assignments shown in panel A. Shaded regions denote 95% confidence intervals, estimated using bootstrap resampling (10^5 iterations). C. Representative structures of each metastable state predicted from simulations. *Q* and *G* values at the cluster center, as well as the type of entanglement change (gain, loss or no change), are shown below each structure image. Color scheme follows that used in Fig. 2B, 2D, and 2F.

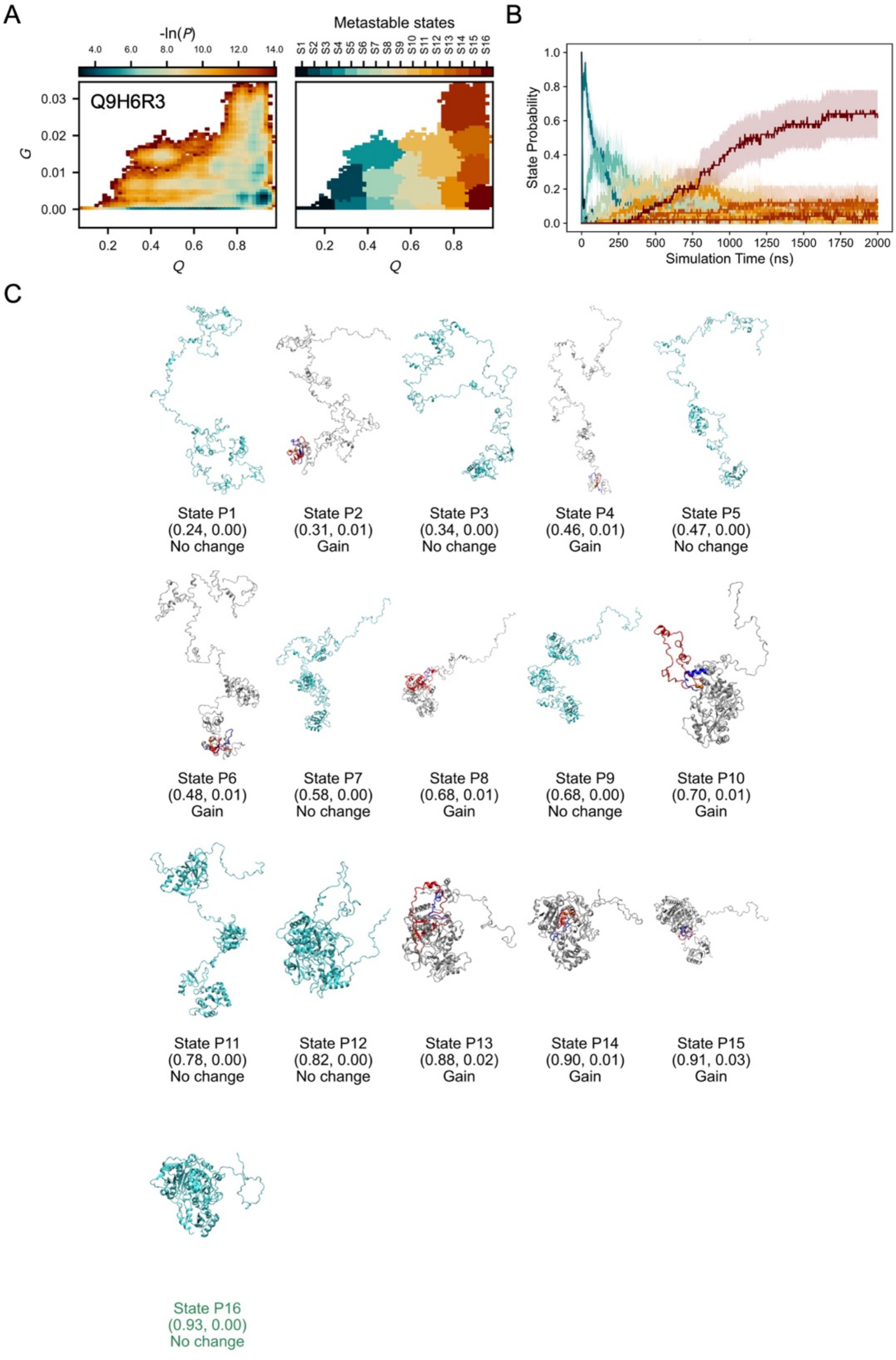

Figure S31. Simulation structural ensemble of the NU-E protein Q9H6R3. A. The probability distribution (-ln*P*) in the *Q* – *G* space (left) and the corresponding metastable states mapped onto structures from temperature-quench refolding simulations (right). B. Time evolution of metastable-state probabilities. Colors match the state assignments shown in panel A. Shaded regions denote 95% confidence intervals, estimated using bootstrap resampling (10^5 iterations). C. Representative structures of each metastable state predicted from simulations. *Q* and *G* values at the cluster center, as well as the type of entanglement change (gain, loss or no change), are shown below each structure image. Color scheme follows that used in Fig. 2B, 2D, and 2F.

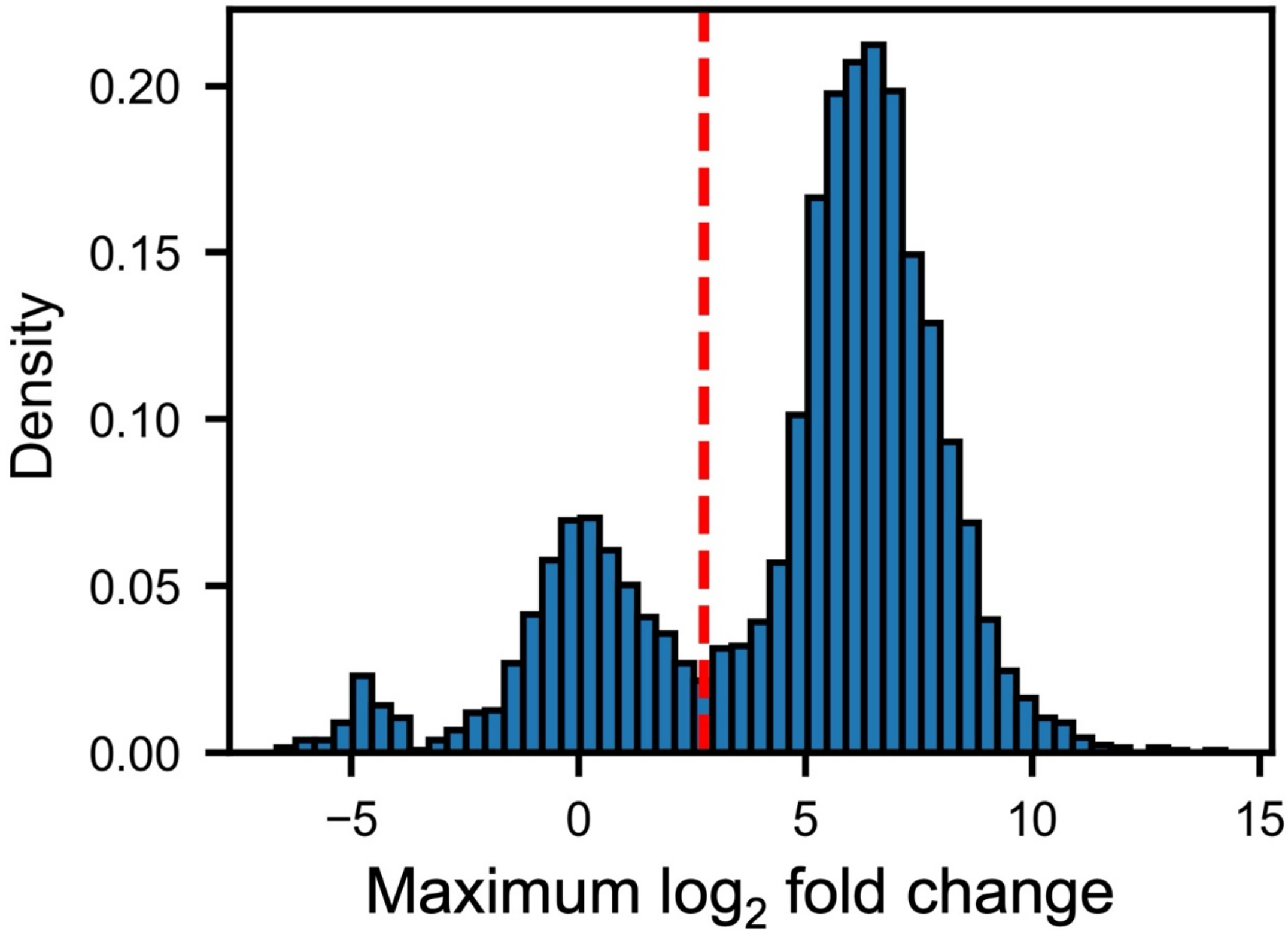

Figure S32. Distribution of the maximum $\log_2$ fold change in Kε-GG peptide abundance (per protein) upon MG132-mediated proteasome inhibition in the birthdating experiments. The red dashed line marks the threshold of 6.75, which separates the two peaks in the distribution.

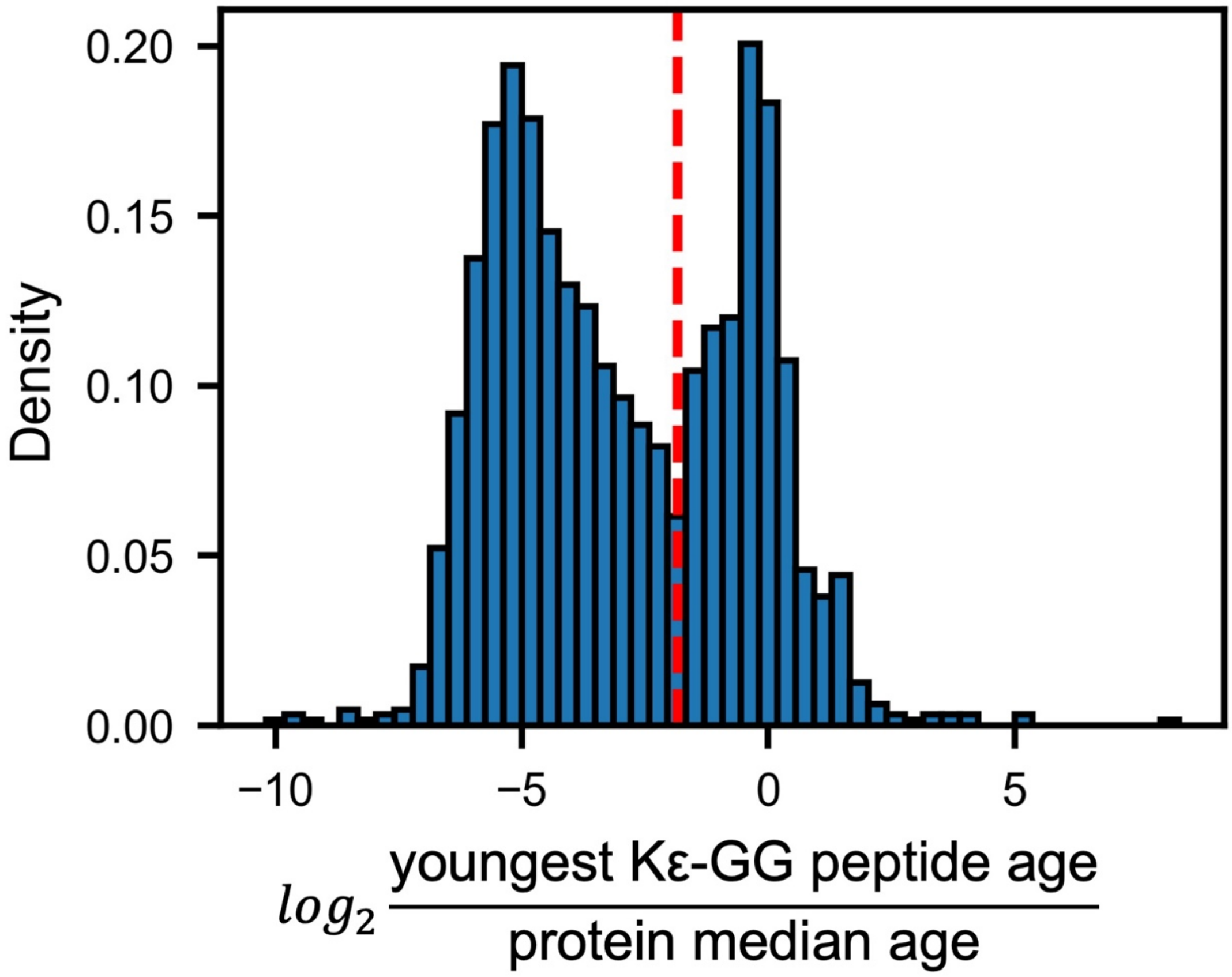

Figure S33. Distribution of the $\log_2$ ratio between the age of the youngest Kε-GG peptide and the protein's median age in the birthdating experiments. The red dashed line marks the threshold of 0.2772, which separates the two peaks in the distribution.

Table S1. Contingency table for young-age ubiquitination within 6 hours (number of proteins).

	Young-age ubiquitination (< 6 hours)	Non-ubiquitination
Entangled protein	431	693
Non-entangled protein	72	294

Table S2. Contingency table for young-age ubiquitination within 1 day (number of proteins).

	Young-age ubiquitination (< 1 day)	Non-ubiquitination
Entangled protein	459	693
Non-entangled protein	82	294

Table S3. Logistic regression results for the Kim datasets. P-values were obtained using two-sided Wald test without adjustment for multiple comparison.

Dataset	IPI to Uniprot protein mapping	Sample size (number of proteins)	Predictor	O.R.	95% CI	p-value
Kim_btz	Legacy official mapping	684	NCLE	1.49	[1.02, 2.17]	0.0392
			Protein Length	1.39	[1.11, 1.75]	0.0042
	Gene name & motif search	839	NCLE	1.85	[1.33, 2.59]	0.0003
			Protein Length	1.47	[1.19, 1.81]	0.0003
Kim_epox	Legacy official mapping	499	NCLE	1.17	[0.75, 1.82]	0.4967
			Protein Length	1.42	[1.08, 1.87]	0.0121
	Gene name & motif search	633	NCLE	1.36	[0.92, 2.00]	0.1192
			Protein Length	1.44	[1.12, 1.84]	0.0039

Table S4. Q_{norm} of the misfolded entanglement states of YU-E and NU-E proteins.

YU-E proteins			NU-E proteins		
Uniprot ID	Number of trajectories w/o mirror images	Q_{norm} [95% CI]	Uniprot ID	Number of trajectories w/o mirror images	Q_{norm} [95% CI]
P31150	50	0.80 [0.74, 0.85]	Q9HB40	50	0.91 [0.89, 0.94]
Q0PNE2	50	0.93 [0.91, 0.96]	P07738	50	0.93 [0.90, 0.96]
A8MXV4	50	0.79 [0.75, 0.82]	P39748	50	0.90 [0.81, 0.98]
P16152	50	0.57 [0.56, 0.59]	Q9UIV1	50	0.99 [0.99, 0.99]
P00491	50	0.69 [0.59, 0.81]	Q9Y316	50	0.97 [0.96, 0.98]
O95394	50	0.98 [0.96, 0.99]	Q96C11	50	0.73 [0.69, 0.78]
P04350	50	0.57 [0.57, 0.57]	Q6DKJ4	42	0.98 [0.96, 1.00]
P19623	44	0.94 [0.93, 0.95]	Q63HM1	50	0.98 [0.97, 0.99]
P29218	50	0.88 [0.83, 0.92]	Q9UBP6	50	0.91 [0.90, 0.92]
P52888	50	0.82 [0.72, 0.93]	Q9H6R3	50	0.96 [0.95, 0.98]
Average	494	0.80 [0.71, 0.88]	Average	492	0.93 [0.87, 0.97]

Table S5. rSASA of the misfolded entanglement states of YU-E and NU-E proteins.

YU-E proteins			NU-E proteins		
Uniprot ID	Number of trajectories w/o mirror images	rSASA [95% CI]	Uniprot ID	Number of trajectories w/o mirror images	rSASA [95% CI]
P31150	50	1.67 [1.48, 1.86]	Q9HB40	50	1.11 [1.07, 1.15]
Q0PNE2	50	1.06 [1.02, 1.10]	P07738	50	1.23 [1.17, 1.31]
A8MXV4	50	1.23 [1.18, 1.29]	P39748	50	1.21 [1.02, 1.41]
P16152	50	2.07 [2.02, 2.11]	Q9UIV1	50	1.03 [1.03, 1.03]
P00491	50	1.63 [1.37, 1.87]	Q9Y316	50	1.09 [1.07, 1.11]
O95394	50	1.04 [1.01, 1.08]	Q96C11	50	1.57 [1.45, 1.66]
P04350	50	2.08 [2.07, 2.09]	Q6DKJ4	42	0.99 [0.95, 1.03]
P19623	44	1.03 [1.00, 1.05]	Q63HM1	50	1.04 [1.03, 1.04]
P29218	50	1.23 [1.14, 1.34]	Q9UBP6	50	1.02 [1.00, 1.04]
P52888	50	1.41 [1.10, 1.62]	Q9H6R3	50	1.05 [1.01, 1.08]
Average	494	1.45 [1.21, 1.70]	Average	492	1.13 [1.04, 1.25]

Table S6. The structural domains and the force field parameters (n_{scal}) of the 10 YU-E proteins (* denotes the instable domain or interface that cannot be stabilized using the highest level of n_{scal} and the median value was used.)

Uniprot ID	Name	Length	Domain and Interfaces	Structural Class	n_{scal}
Q0PNE2	Elongator complex protein 6	266	Domain 1: 1 – 266	α/β	1.1556
P16152	Carbonyl reductase [NADPH] 1	277	Domain 1: 1 – 277	α/β	1.1556
P29218	Inositol monophosphatase 1	277	Domain 1: 1 – 148	α/β	1.1556
			Domain 2: 149 – 277	α/β	1.4213
			1 | 2 Interface	–	1.2747
P00491	Purine nucleoside phosphorylase	289	Domain 1: 1 – 289	α/β	1.1556
P19623	Spermidine synthase	302	Domain 1: 1 – 73	β	1.4732
			Domain 2: 74 – 302	α/β	1.1556
			1 | 2 Interface	–	2.1670
A8MXV4	Acyl-coenzyme A diphosphatase NUDT19	375	Domain 1: 1 – 292	α/β	1.1556
			Domain 2: 293 – 375	α/β	1.1556
			1 | 2 Interface	–	1.8611
P04350	Tubulin beta-4A chain	444	Domain 1: 1 – 260	α/β	1.1556
			Domain 2: 261 – 371	α/β	1.1556
			Domain 3: 372 – 444	α	1.1954
			1 | 2 Interface	–	1.2747
			1 | 3 Interface	–	1.2747
			2 | 3 Interface	–	2.5044
P31150	Rab GDP dissociation inhibitor alpha	447	Domain 1: 1 – 41, 231 – 288, 392 – 447	α/β	1.1556
			Domain 2: 42 – 118, 221 – 230, 289 – 391	α/β	1.1556
			Domain 3: 119 – 220	α	1.1954
			1 | 2 Interface	–	1.2747
			1 | 3 Interface	–	1.2747
			2 | 3 Interface	–	1.2747
O95394	Phosphoacetylglucosamine mutase	542	Domain 1: 1 – 175	α/β	1.1556
			Domain 2: 176 – 297	α/β	1.1556
			Domain 3: 298 – 443	α/β	1.1556
			Domain 4: 444 – 542	α/β	1.4213
			1 | 2 Interface	–	1.2747
			1 | 3 Interface	–	1.2747
			1 | 4 Interface	–	1.2747
			2 | 3 Interface	–	1.2747
			2 | 4 Interface	–	1.2747
			3 | 4 Interface	–	1.8611*
P52888	Thimet oligopeptidase	689	Domain 1: 1 – 154	α	1.1954

	Domain 2: 187 – 246	α/β	1.1556
	Domain 3: 361 – 545, 608 – 689	α/β	1.1556
	Domain 4: 155 – 186, 247 – 360, 546 – 607	α	1.1954
	1 | 2 Interface	–	1.2747
	1 | 3 Interface	–	1.2747
	1 | 4 Interface	–	1.2747
	2 | 3 Interface	–	1.2747
	2 | 4 Interface	–	1.2747
	3 | 4 Interface	–	1.2747

Table S7. The structural domains and the force field parameters (n_{scal}) of the 10 NU-NE proteins (* denotes the instable domain or interface that cannot be stabilized using the highest level of n_{scal} and the median value was used.)

Uniprot ID	Name	Length	Domain and Interfaces	Structural Class	n_{scal}
Q8WV22	Non-structural maintenance of chromosomes element 1 homolog	266	Domain 1: 1 – 90	α/β	1.4213
			Domain 2: 91 – 185	α	1.1954
			Domain 3: 186 – 266	α/β	1.6871*
			1 | 2 Interface	–	1.8611*
			1 | 3 Interface	–	1.2747
			2 | 3 Interface	–	1.2747
A6NDU8	RAB7A-interacting MON1-CCZ1 complex subunit 1	294	Domain 1: 1 – 122	α	1.1954
			Domain 2: 123 – 294	α	1.1954
			1 | 2 Interface	–	1.2747
P30711	Glutathione S-transferase theta-1	240	Domain 1: 1 – 81	α/β	1.1556
			Domain 2: 82 – 240	α	1.1954
			1 | 2 Interface	–	1.2747
Q9BU89	Deoxyhypusine hydroxylase	302	Domain 1: 1 – 139	α	1.1954
			Domain 2: 140 – 302	α	1.1954
			1 | 2 Interface	–	1.5679
Q13825	Methylglutaconyl-CoA hydratase, mitochondrial	339	Domain 1: 1 – 67	α/β	1.1556
			Domain 2: 68 – 279	α/β	1.4213
			Domain 3: 280 – 339	α	1.7453*
			1 | 2 Interface	–	1.8611*
			1 | 3 Interface	–	1.2747
			2 | 3 Interface	–	1.2747
Q6NVY1	3-hydroxyisobutyryl-CoA hydrolase, mitochondrial	386	Domain 1: 1 – 30	α	1.7453*
			Domain 2: 31 – 386	α/β	1.1556
			1 | 2 Interface	–	1.2747
P16520	Guanine nucleotide-binding protein G(I)/G(S)/G(T) subunit beta-3	340	Domain 1: 1 – 340	β	1.4732
Q8IV38	Ankyrin repeat and MYND domain-containing protein 2	441	Domain 1: 1 – 135	α	1.4704
			Domain 2: 136 – 311	α	1.1954
			Domain 3: 312 – 441	α/β	1.6871*
			1 | 2 Interface	–	1.8611*
			1 | 3 Interface	–	1.2747
			2 | 3 Interface	–	2.5044
P02774	Vitamin D-binding protein	474	Domain 1: 1 – 126	α	1.4704
			Domain 2: 127 – 226	α	1.1954
			Domain 3: 227 – 409	α	1.1954
			Domain 4: 410 – 474	α	1.1954

			1 | 2 Interface	–	1.2747
			1 | 3 Interface	–	1.2747
			1 | 4 Interface	–	1.2747
			2 | 3 Interface	–	1.2747
			2 | 4 Interface	–	1.5679
			3 | 4 Interface	–	1.2747
Q12996	Cleavage stimulation factor subunit 3	717	Domain 1: 1 – 233	α	1.1954
			Domain 2: 234 – 337	α	1.1954
			Domain 3: 338 – 558	α	1.1954
			Domain 4: 559 – 717	α/β	1.6871*
			1 | 2 Interface	–	2.1670
			1 | 3 Interface	–	1.2747
			1 | 4 Interface	–	1.2747
			2 | 3 Interface	–	1.2747
			2 | 4 Interface	–	1.5679
			3 | 4 Interface	–	1.2747

Table S8. The structural domains and the force field parameters (n_{scal}) of the 10 NU-NE proteins (* denotes the instable domain or interface that cannot be stabilized using the highest level of n_{scal} and the median value was used.)

Uniprot ID	Name	Length	Domain and Interfaces	Structural Class	n_{scal}
P07738	Bisphosphoglycerate mutase	259	Domain 1: 1 – 259	α/β	1.1556
Q9UIV1	CCR4-NOT transcription complex subunit 7	285	Domain 1: 1 – 285	α/β	1.1556
Q9UBP6	tRNA (guanine-N(7)-)-methyltransferase	276	Domain 1: 1 – 276	α/β	1.1556
Q9Y316	Protein MEMO1	297	Domain 1: 1 – 297	α/β	1.1556
Q63HM1	Kynurenine formamidase	303	Domain 1: 1 – 303	α/β	1.1556
P39748	Flap endonuclease 1	380	Domain 1: 1 – 216	α/β	1.1556
			Domain 2: 217 – 285	α	1.1954
			Domain 3: 286 – 380	α	1.1954
			1 | 2 Interface	–	1.2747
			1 | 3 Interface	–	1.2747
			2 | 3 Interface	–	1.2747
Q6DKJ4	Nucleoredoxin	435	Domain 1: 1 – 169	α/β	1.1556
			Domain 2: 170 – 311	α/β	1.1556
			Domain 3: 312 – 435	α/β	1.1556
			1 | 2 Interface	–	1.8611*
			1 | 3 Interface	–	1.2747
			2 | 3 Interface	–	1.2747
Q9HB40	Retinoid-inducible serine carboxypeptidase	452	Domain 1: 1 – 204, 347 – 452	α/β	1.1556
			Domain 2: 205 – 346	α	1.1954
			1 | 2 Interface	–	1.2747
Q96C11	FGGY carbohydrate kinase domain-containing protein	551	Domain 1: 1 – 257	α/β	1.1556
			Domain 2: 258 – 333, 438 – 551	α/β	1.1556
			Domain 3: 334 – 437	α/β	1.1556
			1 | 2 Interface	–	1.2747
			1 | 3 Interface	–	1.2747
			2 | 3 Interface	–	1.2747
Q9H6R3	Acyl-CoA synthetase short-chain family member 3, mitochondrial	686	Domain 1: 1 – 556	α/β	1.1556
			Domain 2: 557 – 686	α/β	1.4213
			1 | 2 Interface	–	1.8611*

Summary table for MD simulations

**Reliability and reproducibility checklist for molecular dynamics simulations**<br>***All boxes must be marked YES by acceptance unless "Response not needed if No".**	**Yes**	**No**	**Response**<br>**(Please state where this information can be found in the text)**
**1. Convergence of simulations and analysis**			
1a. Is an evaluation presented in the text to show that the property being measured has equilibrated in the simulations (*e.g.* time-course analysis)?	☒	☐	State probabilities as a function of simulation time for the temperature-quench simulations of each protein are shown in panel B of Figs. S1–S20 and S22–S31. Misfolding propensity, Q_norm, and the corresponding p-values for comparisons over simulation time are presented in Fig. S21.
1b. Then, is it described in the text how simulations are split into equilibration and production runs and how much data were analyzed from production runs?	☒	☐	Methods section
1c. Are there at least 3 simulations per simulation condition with statistical analysis?	☒	☐	Methods section
1d. Is evidence provided in the text that the simulation results presented are independent of initial configuration?	☐	☒	Not needed as the initial configuration for each protein's simulation is its native structure, which is unique to each other.
**2. Connection to experiments**			
2a. Are calculations provided that can connect to experiments (*e.g.* loss or gain in function from mutagenesis, binding assays, NMR chemical shifts, J-couplings, SAXS curves, interaction distances or FRET distances, structure factors, diffusion coefficients, bulk modulus and other mechanical properties, *etc.*)?	☒	☐	Misfolding propensities predicted from the simulations are consistent with the trend of ubiquitination and degradation observed in the birth-dating Ubq-MS data. See Fig.2.
**3. Method choice**			
3a. Do simulations contain membranes, membrane proteins, intrinsically disordered proteins, glycans, nucleic acids, polymers, or cryptic ligand binding?	☐	☒	Not needed
3b. Is it described in the text whether the accuracy of the chosen model(s) is sufficient to address the question(s) under investigation (e.g. all-atom vs. coarse-grained models, fixed charge vs. polarizable force fields, implicit vs. explicit solvent or membrane, force field and water model, etc.)?	☒	☐	In the method section we cited our previous studies which demonstrated the realistic of the model.
3c. Is the timescale of the event(s) under investigation beyond the brute-force MD simulation timescale in this study that enhanced sampling methods are needed?	☐	☒	
If **YES**, are the parameters and convergence criteria for the enhanced sampling method clearly stated?	☐	☐	
If **NO,** is the evidence provided in the text?	☐	☒	We are studying the non-equilibrium folding events instead of the equilibrated protein conformations. Therefore enhanced sampling methods are not needed.
**4. Code and reproducibility**			
4a. Is a table provided describing the system setup that includes simulation box dimensions, total number of atoms, total number of water molecules, salt concentration, lipid composition (number of molecules and type)?	☐	☒	The CG simulations do not have PBC and other solvent molecules.
4b. Is it described in the text what simulation and analysis software and which versions are used?	☒	☐	Provided in the Github repository

4c. Are other parameters for the system setup described in the text, such as protonation state, type of structural restraints if applied, nonbonded cutoff, thermostat and barostat, etc.?	☒	☐	Method section
4d. Are initial coordinate and simulation input files and a coordinate file of the final output provided as supplementary files or in a public repository?	☒	☐	Input files are available in the Github repository and trajectories are available at CyVerse.
4e. Is there custom code or custom force field parameters?	☒	☐	
If **YES**, are they provided as supplementary files or in a public repository?	☒	☐	Provided in the Github repository
