# Supplementary material for: Protein entanglement misfolding influences whether proteins undergo proteasomal degradation or persist in near-native misfolded states
Source: Nat Commun. 2026 Aug 25;17:10135. doi: 10.1038/s41467-026-76875-9 (PMC13601551; doi:10.1038/s41467-026-76875-9)
Supplement: Supplementary file 2 [file 41467_2026_76875_MOESM4_ESM.pdf]

nature portfolio

Peer Review File

Protein entanglement misfolding influences whether proteins undergo proteasomal degradation or persist in near-native misfolded states

Corresponding Author: Professor Edward O'Brien

This file contains all reviewer reports in order by version, followed by all author rebuttals in order by version.

Attachments originally included by the reviewers as part of their assessment can be found at the end of this file.

Version 0:

Reviewer comments:

Reviewer #1

(Remarks to the Author)
In this manuscript, Jiang et al. perform bioinformatic and structural analyses of previously reported ubiquitinated proteins identified by mass spectrometry. The authors propose that nascent proteins containing native entanglements are more prone to misfolding and are therefore more frequently ubiquitinated and degraded by proteasomes. They further suggest that a substantial fraction of entangled proteins may misfold into native-like conformations that evade recognition by the cellular quality-control machinery, such as chaperones and E3 ligases. Overall, the study presents interesting observations derived from structural modeling and simulations. However, it remains unclear whether the predicted mechanisms occur in living cells. As this reviewer is not an expert in bioinformatics or large-scale computational modeling, I will focus primarily on conceptual concerns and the lack of experimental validation supporting the central conclusions.

1. The bioinformatic and structural analyses are primarily based on a single global ubiquitin mass spectrometry dataset (Meadow et al., 2024). However, relying on a single study raises concerns about potential bias or dataset-specific artifacts. Independent validation using additional Ubq-MS datasets or orthogonal approaches will strengthen the generality of the conclusions.
2. Previous quantitative mass spectrometry work (e.g., McShane et al., 2016) demonstrated that many newly synthesized proteins are degraded because they fail to assemble with their binding partners. These orphan proteins often expose hydrophobic surfaces or degrons that are recognized by molecular chaperones and E3 ligases, leading to ubiquitination and degradation. This concept is well supported by the discovery of many quality control pathways that recognize them.
3. In contrast, the present manuscript largely attributes early ubiquitination to entanglement-related misfolding, but gives limited consideration to these established mechanisms. It remains unclear whether the observed ubiquitination events can be separated from partner-dependent instability, hydrophobic exposure, or other canonical quality control signals. Without considering these alternative explanations, the interpretation that entanglement misfolding is a primary driver of ubiquitination may be overstated.
4. The central conclusions are based on correlations between structural entanglement features, computational folding simulations, and ubiquitination status. However, no direct evidence is provided that misfolded entanglement states are actually recognized by quality control machinery in cells. The manuscript would be significantly strengthened by experimental validation demonstrating that altering entanglement status affects ubiquitination or degradation in vivo. As currently presented, the mechanistic link between entanglement misfolding and proteasomal targeting remains speculative.
5. The authors define proteins degraded within 6 hours as “young” or nascent. However, many proteins fold within minutes of synthesis and become functional rapidly. Their subsequent degradation may reflect intrinsic half-life and regulated turnover rather than folding failure. An alternative explanation is that proteins with native entanglements may harbor structural features that increase conformational dynamics or long-term instability, thereby transitioning from native to misfolded states over time.

(Remarks on code availability)

Reviewer #2

(Remarks to the Author)

This is an interesting and potentially important paper that follows on from a publication from the O'Brien group in December 2025. In that paper, they establish that proteins which have more complex topologies which involve non-covalent lasso entanglements (NCLEs), have a higher propensity to form a variety of misfolded states, than those without these types of entanglement. This paper used experimental data collected from bacterial proteomes. Here, they extend the work by assessing the degree to which proteins with NCLEs and which misfold are targeted for proteasomal degradation by the ubiquitin system in a human cell line. They conclude that proteins that misfold in this way are approximately twice as likely to be ubiquitinated in a pattern consistent with targeting to the proteasome compared with proteins without NCLEs. Consistently, they find that ubiquitinate proteins are four-times more likely to misfold than non-ubiquitinated proteins without entanglements. Remarkably they provide statistical evidence that about 1/3 of the globular proteome likely misfolds this way but bypasses ubiquitin/degradation as they adopt misfolded states that are structurally very similar to their native states. This result has potential implications for cellular proteostasis.

Comments (sentences from the manuscript are shown in red, reviewers comments are in black).

1. Line 63. These misfolded states exhibit extremely slow folding kinetics, with folding times ranging from seconds to days or longer.
A reference is needed. Also, it is difficult to see how the very long folding times shown in the Figure are estimated. Some description on how this is done and how reliable it is, should be given.

2. Lines 98-99. A recent study combined Ubq-MS with time-resolved metabolic labeling to investigate the prevalence 98 of ubiquitination of nascent proteins in human fibroblasts (19).

Given that the entire study is wholly reliant on the experimental Ubq-MS database, there should be more discussion of its accuracy, reproducibility, limitations etc.

3. Lines 115-116. In the mass spectrum data (19) from human fibroblast cells there are 6,450 observable proteins common across all conditions (see Dataset S1), 2,280 have high quality AlphaFold structures (average pLDDT ≥ 85).

The result that only 35% of the proteins identified by MS in the experimental studies have reliable AF-predicted structures is surprising. This means, 65% of the MS data is not used. How might this bias the results obtained? Are there, for example, large classes/families of proteins that are not included in the analysis?

4. Lines 120-121. ..906 were identified as young-age ubiquitination – of which 507 have high quality AlphaFold structures. Amongst these high-quality structures, 432 have native NCLEs, 3 have covalent lassos and 1 has a knot.

The consequence of these numbers is that only 35/507 have no type of entanglement but these are a essential reference group. How does this affect the overall statistical significance of the results?

5. Lines 160-163. Young ubiquitinated, entangled proteins are more prone to misfolding. To test our hypothesis that natively entangled proteins misfold more often – ultimately enhancing their susceptibility to UPS-mediated degradation - we performed coarse-grained (CG) molecular dynamics (MD) simulations (2) on a representative set of 20 human proteins.

No information is given in the main text on how the 20 proteins subjected to MD simulation were selected. This should be described so readers can assess whether any bias may have been introduced in this step.

6. Line 163. ….calculated their propensity to misfold at the end of 2 μs of simulation time.

Two microseconds may be considered a long time with respect to computational simulations, given the computational burden involved. However, assessing whether all 20 proteins have misfolded at this time needs to be justified. Many cellular proteins fold slowly especially in comparison with many of the "model" proteins whose folding rates were studied in the 1990s. Overall these are small and have simple topologies relative to most cellular proteins. Can the authors be certain that all the 20 proteins simulated have reached a long-lived misfolded state? There is quite a lot of experimental evidence that a number of proteins can populate intermediates states on their folding pathways which can to different degrees look misfolded, but these proteins do fold productively to the native state given a little more time. There are concerns that with longer simulations some of these 20 proteins would indeed successfully fold.

7. Lines 259-263. A criticism of our simulations is that temperature-quench refolding is not the same process as co-translational folding, which can occur in fibroblast cells. To probe whether the misfolded states are similar on- and off- the

ribosome, and whether YU-E proteins misfold more frequently than NU-NE proteins during co-translational folding we performed protein synthesis simulations on the ribosome, followed by post-translational folding.

Whilst it is good to see that the authors have tried to address whether their folding simulations (using temperature-quench methods) are realistic and the same as simulations of folding either co- or post-translationally after ribosomal synthesis, they do not fully address the issue of the real in vivo environment in which the proteins detected in the MS assays are folding. Both trigger factor and a number of other chaperones will be present. I am not expecting the authors to try to simulate the folding of these proteins in the presence of chaperones, but some discussion on what the effects may be would be useful. Are any of the 20 proteins known substrates of the chaperone machinery? Also, it is not clear why only post-translational folding appears to be considered and not co-translational folding.

8. Lines 337-338. …..contain one or more native NCLEs. Of these, 49% (557 out of 1,135) get ubiquitinated for proteasome degradation (proteins with accumulated Kε-GG peptides upon proteasome inhibition) and 44% (497 out of 1,135) do not get ubiquitinated.

Perhaps, I have missed this somewhere in the text, but is there are clear demarcation that the 44% of misfolded states that do not get ubiquitinated all have a native-like structure? Are there characteristic properties of the misfolded structures which get ubiquitinated versus those that do not, e.g., exposed hydrophobic surface area?

9. Lines 339-340. The remaining 7% are tagged with ubiquitin but are not targeted for proteasome degradation, instead the ubiquitination pattern signals a different cellular fate.

This is an interesting observation which is not analysed in any depth. More detail should be given about this 7%, for example, how does their ubiquitination pattern differ, what is their cellular fate? Are there any characteristics in this set of proteins that can distinguish them from the other UPS-targeted misfolded proteins?

10. Lines 356-357. ….to include an additional state that many proteins appear to populate: widespread entanglement-misfolded states that are soluble and non-functional, and persist inside cells for long time periods as they are not successfully acted upon by degradation and chaperone pathways.

It is assumed that all of these misfolded but near-native like states are non-functional. What is the evidence for this? Although in general I can see that misfolded states are likely to be non-functional, I can think of examples (multi-domain proteins) where some activity might be retained.
This assumption should be better justified and/or discussed.

General.

The paper is very thought provoking and the question that instantly comes to mind having read the manuscript, is this: if there are a large number of NCLEs in proteins, and proteins with NCLEs are much more likely to misfold and be targeted for ubiquitination, why has Nature adopted so many structures with NCLEs? It seems very inefficient (Nature isn't always as efficient as we think it should be), but still it is an interesting question.

For this type of increasingly common computational analysis of a large experimental database combined with MD simulations, it would be great if the authors could suggest an experiments/experimental approaches which could be used to test the conclusions.

One summary of the results could be "Many cellular proteins misfold and are degraded, a very large fraction of cellular proteins contain NCLEs and these are even more likely to be misfolded than non-NCLE proteins" thus "Many cellular proteins misfold and are targetted for degradation", which we already know. For me, the crux and the more interesting result is that proteins with NCLEs particularly those that misfold to structures that are near-native and which bypass the chaperone/UPS, have an effect on cellular health.

(Remarks on code availability)

Reviewer #3

(Remarks to the Author)
The manuscript presents and explores how changes in protein entanglement influence the fate of newly synthesized proteins. By combining Ubq-MS structural analysis, and folding simulations, the authors propose that proteins containing native NCLEs are more likely to misfold than proteins that do not contain them. The idea that topological features of protein structures can shape degradation outcomes is very interesting, and the integration of large-scale proteomics with simulation work makes contributes to our understanding of proteostasis.

(Remarks on code availability)

Version 1:

Reviewer comments:

Reviewer #1

(Remarks to the Author)
The authors have addressed my concerns using further bioinformatic approaches. However, without experimental validation demonstrating that altering the entanglement status affects ubiquitination or degradation in cells, the findings remain speculative and are more appropriate for a bioinformatics-focused journal.

(Remarks on code availability)

Reviewer #2

(Remarks to the Author)
The authors have gone to some lengths to address all the comments of the three reviewers. In doing so, they have produced a much more robust manuscript. It is particularly good to see that the results can largely be reproduced with different experimental datasets, and in the case where the dataset does not produce statistically significant results, that there is a reasonable explanation of why not.

Despite the fact that the study remains correlative not causal, it is an important contribution to the field, and provides an interesting hypothesis that can be researched further and validated experimentally. I would recommend the paper be published.

(Remarks on code availability)

Reviewer #3

(Remarks to the Author)
1) The ten-fold extension of simulations (from 2 μs to 20 μs) and the confirmation that key conclusions remain unchanged across the extended trajectories adequately addresses my concern. The clarification regarding the use of non-ubiquitinated proteins as a negative control throughout the analyses is also satisfactory.

2) I appreciate the authors' decision to make the entanglement assignments publicly available through the GitHub repository, along with the addition of a clear statement in the manuscript directing readers to that resource.

3) The comparison of misfolded ensembles between temperature-quench refolding and explicit co- and post-translational folding simulations for representative proteins is a reasonable justification for the chosen approach. The added discussion acknowledging the limitations of the modeling framework, including the absence of chaperones and the scope of the analysis to globular monomeric proteins, is a welcome addition.

4) The authors' response regarding generalizability to other cell types and organisms is well-reasoned, and I appreciate the transparent discussion of factors that could modulate the observed effects. The clarification that knotted proteins, including those with shallow knots, were excluded from the analyses is satisfactory, and I agree that exploring shallow knots in relation to protein quality control represents a worthwhile direction for future work.

5) I am satisfied with the authors' candid acknowledgement that the simulations do not directly model E3 ligase recognition, and that the conclusions rest on indirect evidence. The added text clarifying this limitation, and the mechanistic reasoning for why near-native misfolded states may evade quality-control detection, appropriately clarify my concerns.

(Remarks on code availability)

Open Access This Peer Review File is licensed under a Creative Commons Attribution 4.0 International License, which permits use, sharing, adaptation, distribution and reproduction in any medium or format, as long as you give appropriate credit to the original author(s) and the source, provide a link to the Creative Commons license, and indicate if changes were made.
In cases where reviewers are anonymous, credit should be given to 'Anonymous Referee' and the source.
The images or other third party material in this Peer Review File are included in the article's Creative Commons license, unless indicated otherwise in a credit line to the material. If material is not included in the article's Creative Commons license and your intended use is not permitted by statutory regulation or exceeds the permitted use, you will need to obtain permission directly from the copyright holder.
To view a copy of this license, visit https://creativecommons.org/licenses/by/4.0/

Reviewer comments:

Reviewer #1:

We thank the reviewer for their time and thoughtful comments. We have addressed their points and believe it has strengthened the paper.

In this manuscript, Jiang et al. perform bioinformatic and structural analyses of previously reported ubiquitinated proteins identified by mass spectrometry. The authors propose that nascent proteins containing native entanglements are more prone to misfolding and are therefore more frequently ubiquitinated and degraded by proteasomes. They further suggest that a substantial fraction of entangled proteins may misfold into native-like conformations that evade recognition by the cellular quality-control machinery, such as chaperones and E3 ligases. Overall, the study presents interesting observations derived from structural modeling and simulations. However, it remains unclear whether the predicted mechanisms occur in living cells. As this reviewer is not an expert in bioinformatics or large-scale computational modeling, I will focus primarily on conceptual concerns and the lack of experimental validation supporting the central conclusions.

1. The bioinformatic and structural analyses are primarily based on a single global ubiquitin mass spectrometry dataset (Meadow et al., 2024). However, relying on a single study raises concerns about potential bias or dataset-specific artifacts. Independent validation using additional Ubq-MS datasets or orthogonal approaches will strengthen the generality of the conclusions.

Authors' response: We agree that validation using independent datasets is essential to assess the robustness and generality of conclusions drawn from the Meadow et al. (2024) proteome birthdating dataset. We have now expanded the manuscript to include analyses of three additional independent human ubiquitination mass-spectrometry datasets from two prior studies. Specifically, we analyzed one dataset from Wagner et al. and two datasets from Kim et al., the latter generated using two distinct proteasome inhibitors. Using the same overall framework as in our primary analysis, we curated proteins with high-quality AlphaFold structures, classified proteins as ubiquitinated or non-ubiquitinated based on enrichment of Kε-GG peptides upon proteasome inhibition and repeated the logistic regression analyses while controlling for protein length.

We observee *consistent* positive associations between native NCLEs and ubiquitination in two of the three independent datasets. In the Wagner dataset, proteins containing NCLEs were significantly more likely to be ubiquitinated (OR = 3.11, 95% CI: [2.20, 4.39], $p < 0.0001$; n = 821 proteins). A similar significant association was observed in the Kim_btz dataset (OR = 1.85, 95% CI: [1.33, 2.59], p = 0.0003; n = 839 proteins). The Kim_epox dataset showed the same positive trend but did not reach statistical significance (OR = 1.36, 95% CI: [0.92, 2.00], p = 0.1192; n = 633 proteins), likely due to its smaller sample size and lower statistical power.

These new results have been added to the Results and Methods sections. Collectively, they demonstrate that the association between native NCLE and ubiquitination is reproducible across multiple independent Ubq-MS datasets and is therefore unlikely to arise from dataset-specific artifacts in Meadow et al. (2024).

Added text: "**Association is robust across independent ubiquitination datasets.** To assess whether the observed association between native NCLEs and protein ubiquitination generalizes across independent human datasets, we analyzed three additional ubiquitination datasets, which

we refer to as the Wagner dataset (27), and the Kim_btz and Kim_epox datasets (28). The latter two were generated in the same study using different proteasome inhibitors. Unlike the dataset from Ref. (19), which combines metabolic labeling with ubiquitin enrichment and therefore distinguishes young from old ubiquitinated proteins, these additional datasets quantify ubiquitinated proteins accumulated upon proteasome inhibition without age partitioning. However, the Meadow study showed that young ubiquitinated proteins are degraded substantially more rapidly by the proteasome than older ubiquitinated proteins (19). Consequently, proteasome inhibition preferentially enriches for ubiquitinated proteins from the young protein population, providing a rationale for comparing these datasets with the young-age ubiquitination analyses performed above. Using a consistent post-processing pipeline (see Methods), we identified ubiquitinated and non-ubiquitinated proteins in each data set that had corresponding high-quality AlphaFold structures and performed logistic regression analyses controlling for protein length.

We observed a significant positive association between native NCLEs and ubiquitination in both the Wagner dataset (OR = 3.11, 95% CI: [2.20, 4.39], $p < 0.0001$, Wald test; $n = 821$ unique proteins) and the Kim_btz dataset (OR = 1.85, 95% CI: [1.33, 2.59], $p = 0.0003$, Wald test; $n = 839$). The Kim_epox dataset showed a similar trend, but the association did not reach statistical significance (OR = 1.36, 95% CI: [0.92, 2.00], $p = 0.1192$, Wald test; $n = 633$). We attribute the lack of statistical significance in the Kim_epox dataset to its smaller sample size and consequently reduced statistical power. Consistent with this interpretation, further reducing the sample size using an alternative IPI-to-UniProt mapping (see Methods) resulted in weaker statistical significance in both the Kim_btz and Kim_epox datasets (see Table S3).

Overall, proteins containing native entanglements are estimated to be 85% to 211% more likely to be ubiquitinated across two of the three independent datasets, supporting the robustness of this association in the human proteome."

2. Previous quantitative mass spectrometry work (e.g., McShane et al., 2016) demonstrated that many newly synthesized proteins are degraded because they fail to assemble with their binding partners. These orphan proteins often expose hydrophobic surfaces or degrons that are recognized by molecular chaperones and E3 ligases, leading to ubiquitination and degradation. This concept is well supported by the discovery of many quality control pathways that recognize them.

Authors' response: We thank the reviewer for highlighting the important role of orphan protein degradation and assembly-coupled quality control in shaping the fate of newly synthesized proteins. We agree that such mechanisms - along with ribosome pausing, disome formation, and other translation-associated quality control pathways – can contribute significantly to young-age ubiquitination. To address this, we have revised the Discussion to explicitly acknowledge these alternative, non-mutually exclusive pathways. We emphasize that our proposed mechanism - misfolding driven by non-covalent lasso entanglements - does not exclude these processes but instead provides a complementary structural explanation for a large subset of early ubiquitination events. For example, misfolding involving NCLE's often involves a corresponding increase in hydrophobic surface area.

Added text: "Ubiquitination does not just arise from misfolding, it can also arise from several non-mutually exclusive mechanisms. Previous studies have shown that newly synthesized proteins may be degraded due to failure to assemble with binding partners (26) (known as orphan proteins), while ribosome pausing or collisions can trigger ribosome-associated quality control pathways (34), including disome-associated surveillance mechanisms (35). These processes can expose

hydrophobic regions or degrons that are subsequently recognized by molecular chaperones and E3 ligases. Our findings suggest that misfolding driven by NCLEs represents an additional, complementary mechanism that contributes to early ubiquitination, rather than being mutually exclusive with these established pathways."

3. In contrast, the present manuscript largely attributes early ubiquitination to entanglement-related misfolding, but gives limited consideration to these established mechanisms. It remains unclear whether the observed ubiquitination events can be separated from partner-dependent instability, hydrophobic exposure, or other canonical quality control signals. Without considering these alternative explanations, the interpretation that entanglement misfolding is a primary driver of ubiquitination may be overstated.

Authors' response: As noted in our response above, we have revised the Discussion to explicitly acknowledge these alternative, non-mutually exclusive mechanisms. Importantly, our goal is not to claim that entanglement-related misfolding is the sole driver of early ubiquitination, but rather to demonstrate that it represents a previously unrecognized structural determinant that can contribute to this process. In particular, our results show that proteins enriched in native NCLEs exhibit higher misfolding propensity and are more likely to undergo early ubiquitination, providing an explanation for our observation of a statistically significant association between entanglement and degradation susceptibility.

To further address the reviewer's concern regarding potential confounding by partner-dependent instability, we performed an additional analysis controlling for protein monomeric status. Specifically, we curated human proteins with known oligomer state as a monomer (n = 402), annotated our dataset accordingly (monomeric vs. oligomeric), and repeated the logistic regression with monomeric status included as a covariate. The results (Table R1) show that the association between entanglement and early ubiquitination remains essentially unchanged (odds ratio = 1.94 vs. 1.93 without this control for proteins ubiquitinated within 6 hours). In contrast, monomeric status itself is not significantly associated with early ubiquitination (adjusted p = 0.61), suggesting that oligomerization-dependent instability is unlikely to confound the observed relationship between entanglement and ubiquitination.

Table R1. Odds ratio and p-value of logistic regression with controlling protein monomeric status

	Coefficient	Odds Ratio	Lower CI	Upper CI	p-value	Adjusted p-value
Entanglement	0.6644	1.9433	1.4421	2.6187	0	0
Protein length	0.4684	1.5974	1.3872	1.8396	0	0
Monomeric status (yes/no)	-0.0968	0.9077	0.6277	1.3128	0.6071	0.6071

While we cannot fully disentangle all contributing factors at the proteome scale, these results support that entanglement-driven misfolding contributes independently and complements existing mechanisms. We have revised the text to moderate the wording accordingly and to better reflect this interpretation.

Added text in Results Section: "**Oligomeric status does not confound NCLE–ubiquitination association.** We next tested whether oligomeric status (monomeric versus oligomeric, curated

from Uniprot) influences our results (26) by including it as an additional confounder in the logistic regression model. We find this does not alter the association between entanglement and early ubiquitination (OR =1.94 versus 1.93 without this confounder), and monomeric status is not significantly associated (adjusted p = 0.61) with ubiquitination status. This indicates that the observed effect is not driven by oligomerization-dependent instability."

Added text in Discussion Section: "When we controlled for oligomeric status, the association between entanglement and early ubiquitination was unaltered, indicating the findings in this study are not driven by oligomerization-dependent instability."

4. The central conclusions are based on correlations between structural entanglement features, computational folding simulations, and ubiquitination status. However, no direct evidence is provided that misfolded entanglement states are actually recognized by quality control machinery in cells. The manuscript would be significantly strengthened by experimental validation demonstrating that altering entanglement status affects ubiquitination or degradation in vivo. As currently presented, the mechanistic link between entanglement misfolding and proteasomal targeting remains speculative.

Authors' response: We agree this is an observational study and that direct experimental validation demonstrating that altering entanglement status affects ubiquitination or degradation in vivo would provide the most definitive test of the proposed mechanism. As an observation study we identify a robust association and propose a mechanism that can explain the observed trends. Specifically, our analyses integrate proteome-wide ubiquitination data, structural analyses, and molecular simulations to show that (i) proteins containing native NCLEs are more likely to undergo early ubiquitination, (ii) these proteins are more prone to populate misfolded states involving entanglement changes, and (iii) some of these states can persist as long-lived, near-native conformations that may evade recognition by quality-control pathways. We have revised the manuscript to more clearly state that our conclusions are correlative and imply - but do not demonstrate - causality.

As an aide to experimentalists who might be interested in building on our work, we have expanded the Discussion to outline several experimental strategies that could test the proposed mechanism. These include LiP–MS experiments under proteasome inhibition to detect conformational changes associated with misfolded states, and structural characterization of misfolded ensembles using cryo-EM for carefully selected targets.

Added text: "Our study implies but does not establish a causal role of NCLEs in driving young-age ubiquitination. To test this mechanistic link several experimental approaches could be used. Limited proteolysis mass spectrometry (LiP–MS) (39) performed under proteasome inhibition conditions could reveal proteome-wide conformational changes associated with the accumulation of misfolded states, enabling direct comparison between structural perturbations and ubiquitination profiles. Structural methods such as cryo-electron microscopy (cryo-EM) (40) may enable direct characterization of misfolded ensembles involving entanglement changes for carefully selected targets, although such experiments will require proteins with sufficiently stable and less heterogeneous conformational states. "

5. The authors define proteins degraded within 6 hours as "young" or nascent. However, many proteins fold within minutes of synthesis and become functional rapidly. Their subsequent degradation may reflect intrinsic half-life and regulated turnover rather than folding failure. An alternative explanation is that proteins with native entanglements may harbor structural features

that increase conformational dynamics or long-term instability, thereby transitioning from native to misfolded states over time.

Authors' response: We agree that degradation within 6 hours of synthesis does not necessarily imply folding failure, as proteins can fold rapidly and subsequently undergo normal regulated turnover according to their intrinsic half-lives. To minimize this possibility, our classification of young-ubiquitinated proteins was not based solely on absolute age. In addition to requiring ubiquitination within the youngest time bin, we further required that the ubiquitination age be younger than the corresponding baseline protein age, which was estimated from the median age of unmodified peptides for the same protein in the proteome birthdating dataset. Thus, proteins classified as young-ubiquitinated were specifically those whose ubiquitination occurred substantially earlier than the turnover behavior of the bulk protein population. This filtering was designed to enrich for proteins undergoing unusually early degradation rather than proteins with naturally short half-lives or regulated turnover.

We also appreciate the reviewer's alternative interpretation that proteins containing native NCLEs may possess structural features that promote enhanced conformational dynamics or delayed instability, allowing transitions from native to misfolded states over time. We agree that this is plausible and, importantly, not mutually exclusive with our proposed mechanism. In our framework, misfolded states involving entanglement changes span a spectrum of structural perturbations, including relatively destabilized states with lower Q (fraction of native contacts) values and increased conformational dynamics. Such states may arise either directly during folding or through later transitions from the native ensemble, although the latter case is rarely observed in our simulations (out-of-native state transition probability is in general 10^{-6} to 10^{-5} when we calculate it for protein P16152 from our simulations). In both cases, the common key feature is that native NCLEs increase susceptibility to entering degradation-prone non-native conformations.

Reviewer #2:

We thank the reviewer for their time and thoughtful comments. We have addressed their points and believe it has strengthened the paper.

This is an interesting and potentially important paper that follows on from a publication from the O'Brien group in December 2025. In that paper, they establish that proteins which have more complex topologies which involve non-covalent lasso entanglements (NCLEs), have a higher propensity to form a variety of misfolded states, than those without these types of entanglement. This paper used experimental data collected from bacterial proteomes. Here, they extend the work by assessing the degree to which proteins with NCLEs and which misfold are targeted for proteasomal degradation by the ubiquitin system in a human cell line. They conclude that proteins that misfold in this way are approximately twice as likely to be ubiquitinated in a pattern consistent with targeting to the proteasome compared with proteins without NCLEs. Consistently, they find that ubiquitinate proteins are four-times more likely to misfold than non-ubiquitinated proteins without entanglements. Remarkably they provide statistical evidence that about 1/3 of the globular proteome likely misfolds this way but bypasses ubiquitin/degradation as they adopt misfolded states that are structurally very similar to their native states. This result has potential implications for cellular proteostasis.

Comments:

1. Line 63. These misfolded states exhibit extremely slow folding kinetics, with folding times ranging from seconds to days or longer.

A reference is needed. Also, it is difficult to see how the very long folding times shown in the Figure are estimated. Some description on how this is done and how reliable it is, should be given.

Authors' response: We have added the reference in which the folding times were originally estimated. In addition, we included a brief description in the figure caption outlining how these folding times were determined, including the Pearson R^2 cutoff used to ensure high-quality fitting and reliable extrapolation.

Updated text: "The probability distribution of simulation-estimated folding times from these misfolded states - obtained from our previous proteome-wide computational study for *E. coli* (1) - is shown at the bottom. <u>These folding times were extrapolated by fitting the survival probability of unfolded proteins over time with a double-exponential function and we only reported the proteins (n = 73) whose fitting has a Pearson $R^2 > 0.9$ (1).</u>"

2. Lines 98-99. A recent study combined Ubq-MS with time-resolved metabolic labeling to investigate the prevalence 98 of ubiquitination of nascent proteins in human fibroblasts (19).

Given that the entire study is wholly reliant on the experimental Ubq-MS database, there should be more discussion of its accuracy, reproducibility, limitations etc.

Authors' response: We have added the following discussion into the revised manuscript to clarify the accuracy, reproducibility and limitations of the Meadow dataset. Additionally, (see in response to Reviewer 1) in the revised manuscript we demonstrate that these conclusions are robust across three other Ubq-MS datasets from two other labs.

Added text: "We selected the proteins for simulation based on the ubiquitination data from Ref. (19). To the best of our knowledge, this dataset is unique in that it simultaneously profiles the

identity, proteasomal flux, and molecular age of ubiquitinated proteins, enabling identification of the subset of the proteome that is rapidly targeted for proteasomal degradation soon after synthesis. The overall accuracy of the measurements was verified by a battery of control experiments. Nonetheless, several limitations warrant consideration. Because nascent ubiquitinated proteins are inherently transient, their detection required proteasome inhibition, raising the possibility that MG132-induced proteotoxic stress may itself elevate ubiquitination of newly synthesized proteins, complicating the distinction between constitutive ubiquitination and stress-induced ubiquitination. In addition, these experiments were performed in a single quiescent human fibroblast cell line, and whether the observed properties of the ubiquitinome extend to other cell types remains to be determined."

3. Lines 115-116. In the mass spectrum data (19) from human fibroblast cells there are 6,450 observable proteins common across all conditions (see Dataset S1), 2,280 have high quality AlphaFold structures (average pLDDT $\geqslant$ 85).

The result that only 35% of the proteins identified by MS in the experimental studies have reliable AF-predicted structures is surprising. This means, 65% of the MS data is not used. How might this bias the results obtained? Are there, for example, large classes/families of proteins that are not included in the analysis?

Authors' response: We restricted the analysis to proteins with high-quality AlphaFold (AF)–predicted structures for two main reasons. First, our identification of entanglements relies solely on the 3D atomic coordinates of the protein structure. Including low-quality AF models (with low pLDDT), which often contain large disordered or poorly resolved regions, would increase the likelihood of false positives and false negatives. Second, we aimed to exclude intrinsically disordered proteins (IDPs, often have low pLDDTs) from the analysis, as they do not adopt stable native structures and therefore do not have well-defined misfolded states in the context of our framework. Consequently, only the subset of proteins with high-confidence AF structural predictions was included to ensure that the entanglement analysis is reliable and structurally meaningful.

To test if this biases our results, we performed a systematic enrichment analysis comparing proteins included versus excluded from the structural dataset using UniProt functional annotations. The excluded proteins are significantly enriched in classes such as phosphoproteins, coiled-coil proteins, zinc-finger proteins, and DNA-binding/transcription-related proteins, which are known to contain intrinsically disordered or flexible regions and therefore tend to have lower AlphaFold confidence. In contrast, the included proteins are enriched for enzymatic and structured protein classes (e.g., oxidoreductases and domain-rich proteins), which are more amenable to high-confidence structure prediction. Thus, the retained ~35% of proteins are biased toward well-folded, structurally stable proteins, while regulatory and intrinsically disordered protein classes are underrepresented. We have now added text to the manuscript to explicitly acknowledge and discuss this bias.

Added text: "The conclusions of this study are restricted to globular proteins, as our structural analysis necessitated focusing on high-confidence AlphaFold models (average pLDDT $\geqslant$ 85), which represent ~35% of the proteins detected in the Ubq-MS dataset. The excluded proteins are overrepresented in classes such as transcription factors, DNA-binding proteins, zinc-finger proteins, and coiled-coil proteins - categories that often include long intrinsically disordered segments or are conformationally flexible and therefore less amenable to high-confidence

structure prediction. IDPs and regulatory proteins, such as transcription factors, have been found to populate NCLE states (38). So it may be possible these states are also relevant to degradation in these systems."

4. Lines 120-121. ..906 were identified as young-age ubiquitination – of which 507 have high quality AlphaFold structures. Amongst these high-quality structures, 432 have native NCLEs, 3 have covalent lassos and 1 has a knot.

The consequence of these numbers is that only 35/507 have no type of entanglement but these are a essential reference group. How does this affect the overall statistical significance of the results?

Authors' response: In general, smaller sample sizes reduce statistical power and can lead to larger p-values, thereby increasing the likelihood of false negatives, where true associations may fail to reach statistical significance. In our case, although the reference group without entanglements is relatively small (~75 out of 507 proteins), we nevertheless observe statistically significant associations. This indicates that the detected relationships are robust and likely reflect a strong underlying effect despite the limited size of the reference group.

5. Lines 160-163. Young ubiquitinated, entangled proteins are more prone to misfolding. To test our hypothesis that natively entangled proteins misfold more often – ultimately enhancing their susceptibility to UPS-mediated degradation - we performed coarse-grained (CG) molecular dynamics (MD) simulations (2) on a representative set of 20 human proteins.

No information is given in the main text on how the 20 proteins subjected to MD simulation were selected. This should be described so readers can assess whether any bias may have been introduced in this step.

Authors' response: These 20 proteins were randomly selected from the corresponding dataset, with protein lengths approximately matched between the two groups. This information is stated in the sentence following the one quoted by the reviewer. The detailed selection procedure is described in the Methods section titled "Select candidate proteins for temperature quench simulations."

Due to limitations of the simulation model, we restricted the simulations to globular proteins that are neither transmembrane proteins nor proteins likely to be subunits in complexes. We recognize that this requirement may introduce a selection bias, and we have therefore added a statement in the Discussion section to explicitly acknowledge this limitation.

Added text: "Like all models, our simulations have certain limitations. The simulation framework used in this study is designed to model the folding of globular monomeric proteins and therefore does not explicitly represent transmembrane proteins or proteins that are stabilized as subunits within larger complexes present in the Ubq-MS dataset. Nevertheless, it seems plausible that similar misfolded entanglement states may also occur in these protein classes, as they do contain NCLEs (e.g., one-fifth transmembrane protein domains contain NCLEs (37)) that could potentially misfold through mechanisms analogous to those observed in globular proteins (5)."

6. Line 163. ….calculated their propensity to misfold at the end of 2 μs of simulation time.

Two microseconds may be considered a long time with respect to computational simulations, given the computational burden involved. However, assessing whether all 20 proteins have

misfolded at this time needs to be justified. Many cellular proteins fold slowly especially in comparison with many of the "model" proteins whose folding rates were studied in the 1990s. Overall these are small and have simple topologies relative to most cellular proteins. Can the authors be certain that all the 20 proteins simulated have reached a long-lived misfolded state? There is quite a lot of experimental evidence that a number of proteins can populate intermediates states on their folding pathways which can to different degrees look misfolded, but these proteins do fold productively to the native state given a little more time. There are concerns that with longer simulations some of these 20 proteins would indeed successfully fold.

Authors' response: We agree that 2 μs, while substantial compared to many other simulation studies, does not guarantee complete equilibration of folding landscapes for all proteins, particularly for larger cellular proteins that may fold more slowly than the small model systems classically studied. We also agree that some conformations classified as misfolded at intermediate times could, given sufficient time, reach the native state.

Our objective, however, is not to claim that every non-native state observed at 2 μs represents a permanently trapped species, nor that all proteins have fully converged by that time. Rather, our aim is to determine whether proteins enriched in native NCLEs show a greater tendency to populate long-lived, degradation-relevant non-native states on experimentally relevant timescales. In the context of the Ubq-MS experiments, proteins ubiquitinated within the first 6 hours after synthesis need not remain misfolded indefinitely; persistence on the timescale of seconds to hours would already be sufficient to increase susceptibility to recognition by quality-control pathways. This timescale is comparable with our previous estimation of the lifetimes for misfolded entanglement states, which were obtained from both the coarse-grained structure-based model (see Fig. 2C in PMID: 35654797) and unbiased all-atom force-field simulations (see Section "Deep entanglements are usually long lived" in PMID: 36471044 and Fig. 3C in PMID: 40779622).

To directly address the reviewer's concern, we have now extended all simulations ten-fold, from 2 μs to 20 μs, and reanalyzed the trajectories at 1-μs intervals (see new Fig. S21). These extended simulations show that the principal conclusions of the manuscript remain unchanged. First, YU–E proteins consistently exhibit a higher propensity to populate misfolded states involving entanglement changes than NU–NE proteins throughout the extended simulations, with differences remaining statistically significant at most time points examined (Fig. S21A and B). Second, the conclusion that entanglement-associated misfolding is comparably common in both ubiquitinated and non-ubiquitinated entangled proteins also remains unchanged. Third, although some proteins progressively moved toward the native state over longer times, the structural distinction that misfolded states of NU–E proteins are more native-like than those of YU–E proteins persisted throughout the 20 μs simulations (Fig. S21C and D).

These additional data support the interpretation that the states identified at 2 μs are not merely transient early folding intermediates that disappear upon modestly longer sampling. Instead, they represent kinetically persistent non-native ensembles whose relative populations differ systematically between protein classes. We therefore retained the original 2 μs analyses as the primary dataset for consistency, while adding the 20 μs simulations as an explicit robustness test that strengthens the conclusions.

Added text: "To test whether these misfolded entanglement states are long-lived, we extended the simulation time to 20 μs and calculated the misfolding propensity at 1-μs intervals. The resulting time courses show that YU-E proteins consistently exhibit a higher propensity to misfold

than NU-NE proteins throughout the extended simulations, with differences remaining statistically significant at most time points examined (p-value < 0.05; Fig. S21A and B)."

"… We conclude that entanglement misfolding is equally common amongst ubiquitinated and non-ubiquitinated proteins. The same conclusion was obtained when the simulations were extended to 20 μs (Fig. S21A and B)."

"… Thus, NU-E misfolded proteins more closely resemble the native state than YU-E proteins. The difference in Q_{norm} between YU-E and NU-E misfolded proteins tended to decrease when the simulations were extended to 20 μs, as some proteins progressively folded toward the native state. Nevertheless, the same overall trend persisted throughout the extended simulations (Fig. S21C and D)."

7. Lines 259-263. A criticism of our simulations is that temperature-quench refolding is not the same process as co-translational folding, which can occur in fibroblast cells. To probe whether the misfolded states are similar on- and off- the ribosome, and whether YU-E proteins misfold more frequently than NU-NE proteins during co-translational folding we performed protein synthesis simulations on the ribosome, followed by post-translational folding.

Whilst it is good to see that the authors have tried to address whether their folding simulations (using temperature-quench methods) are realistic and the same as simulations of folding either co- or post-translationally after ribosomal synthesis, they do not fully address the issue of the real in vivo environment in which the proteins detected in the MS assays are folding. Both trigger factor and a number of other chaperones will be present. I am not expecting the authors to try to simulate the folding of these proteins in the presence of chaperones, but some discussion on what the effects may be would be useful. Are any of the 20 proteins known substrates of the chaperone machinery? Also, it is not clear why only post-translational folding appears to be considered and not co-translational folding.

Authors' response: We thank the reviewer for raising this important point regarding the cellular environment in which proteins fold. In human fibroblast cells, newly synthesized proteins fold in the presence of ribosome-associated factors and molecular chaperones such as the Hsp70 and Hsp90 systems as well as the TRiC/CCT chaperonin. Our simulations are not intended to explicitly reproduce the full cellular folding environment, but rather to examine the intrinsic propensity of proteins to populate kinetically trapped misfolded states involving noncovalent lasso entanglements.

To evaluate whether the proteins simulated here are known substrates of the TRiC/CCT chaperonin system, we compared the 30 proteins analyzed in this study with curated TRiC/CCT substrate annotations from the Reactome database. Only two of the simulated proteins appear in the currently annotated list of TRiC/CCT substrates, suggesting that most of them do not belong to the class of obligate TRiC-dependent folding clients. Nevertheless, some of these proteins may interact transiently with general chaperone systems such as Hsp70 during folding, as is common for many cytosolic proteins.

Importantly, molecular chaperones generally assist folding by preventing aggregation and promoting refolding cycles rather than guaranteeing correct folding of every substrate. In particular, entanglement misfolding may be difficult to resolve once formed because disentanglement often requires substantial unfolding of folded structural elements. Therefore, the presence of chaperones in vivo does not necessarily preclude the formation or persistence of

kinetically trapped misfolded states. In addition, we have demonstrated in our previous study (PMID: 37344452) that for the near-native misfolded conformations, chaperones may not be able to efficiently recognize and correct the misfolding.

We have clarified this model limitation in the revised manuscript.

Regarding the reviewer's question about co-translational versus post-translational folding, our study does not assume that misfolding occurs exclusively after synthesis. As discussed in the manuscript and depicted in Fig. 3C, misfolded entangled states can arise during both co-translational and post-translational folding.

Added text: "… Further, protein folding in vivo occurs in the presence of molecular chaperones such as the Hsp70/Hsp90 systems and the TRiC/CCT chaperonin, whereas these chaperones are absent from our simulations."

8. Lines 337-338. …..contain one or more native NCLEs. Of these, 49% (557 out of 1,135) get ubiquitinated for proteasome degradation (proteins with accumulated Kε-GG peptides upon proteasome inhibition) and 44% (497 out of 1,135) do not get ubiquitinated.

Perhaps, I have missed this somewhere in the text, but is there are clear demarcation that the 44% of misfolded states that do not get ubiquitinated all have a native-like structure? Are there characteristic properties of the misfolded structures which get ubiquitinated versus those that do not, e.g., exposed hydrophobic surface area?

Authors' response: We agree that an important question is whether the entangled proteins that do not undergo detectable ubiquitination preferentially populate more native-like misfolded states, and whether structural differences distinguish misfolded conformations associated with ubiquitination from those that are not.

To clarify, the statement that 44% of entangled proteins do not undergo detectable ubiquitination refers to the full proteome-scale dataset, whereas detailed structural characterization was performed on representative subsets selected for simulation. Because explicit simulations of all 497 NU–E proteins are computationally impractical, we selected ten representative NU–E proteins matched in size to the YU–E set and examined their folding landscapes in detail.

These simulations show that 9 of the 10 NU–E proteins populate misfolded states involving entanglement changes, indicating that misfolding is also common among non-ubiquitinated entangled proteins. However, these misfolded states are, on average, more native-like than those of YU–E proteins, as quantified by their higher normalized fraction of native contacts (Q_{norm}). Thus, we do not suggest that all non-ubiquitinated entangled proteins misfold to near-native like conformations, but rather that many populate near-native misfolded states that may evade quality-control recognition.

Following the reviewer's suggestion, we have now further analyzed solvent exposure of hydrophobic residues as an additional structural discriminator. Specifically, we quantified the hydrophobic solvent-accessible surface area of misfolded-state ensembles relative to their native-state ensembles. We found that YU–E misfolded proteins exhibit significantly greater hydrophobic surface exposure than NU–E misfolded proteins, consistent with the idea that YU–E proteins populate more structurally perturbed conformations that are more readily recognized by quality-control pathways. In contrast, the lower hydrophobic exposure of NU–E misfolded proteins is consistent with their more near-native character and may contribute to their reduced ubiquitination.

Added text: "In addition, we examined the ratio of the solvent-accessible surface area (rSASA, see Eq. 6; per-protein values are shown in Table S5) of hydrophobic residues in misfolded-state ensembles relative to the corresponding native-state ensembles and compared this metric between YU-E and NU-E misfolded proteins (see Methods). We found that misfolded states in YU-E proteins displayed greater exposure of hydrophobic surface than misfolded NU-E proteins ($\langle rSASA \rangle$ = 1.45, 95% CI: [1.21, 1.70] vs. 1.13, 95% CI: [1.04, 1.25], p-value = 0.0378, two-sided hierarchical permutation test for 106 times, see Methods). The more native-like hydrophobic surface area of misfolded NU-E proteins - consistent with their more near-native conformations - may contribute to their reduced recognition by E3 ubiquitin ligases."

9. Lines 339-340. The remaining 7% are tagged with ubiquitin but are not targeted for proteasome degradation, instead the ubiquitination pattern signals a different cellular fate.

This is an interesting observation which is not analysed in any depth. More detail should be given about this 7%, for example, how does their ubiquitination pattern differ, what is their cellular fate? Are there any characteristics in this set of proteins that can distinguish them from the other UPS-targeted misfolded proteins?

Authors' response: The 7% of ubiquitinated proteins that are not targeted for proteasomal degradation are likely ubiquitinated for regulatory or signaling purposes rather than as a consequence of misfolding. The Ubq-MS experimental technique does not provide information about ubiquitin linkage types or the downstream cellular outcomes of ubiquitination. As described in the manuscript, we identify ubiquitinated proteins that are targeted for degradation as those proteins, that upon proteosome inhibition, increase their abundance of KGG-ubiquitinated fragments. 7% of proteins are ubiquitinated but do not show an increase upon proteosome inhibition, leading to the inference that the ubiquitination of these proteins is likely being used for some other non-degradative process. Since our manuscript is focused on proteins that get degraded, we believe it is outside the scope of the present study to focus on this group of proteins. Community members that would like to focus on this have access to the lists of these proteins in Supplementary Dataset S2. We have added a clarification in the manuscript to note that this subset likely reflects ubiquitination events unrelated to misfolding and proteasomal degradation.

Updated text: "The remaining 7% are tagged with ubiquitin but are not targeted for proteasome degradation. (These likely reflect non-degradative functions of ubiquitin, such as regulatory or signaling roles.)"

10. Lines 356-357. ….to include an additional state that many proteins appear to populate: widespread entanglement-misfolded states that are soluble and non-functional, and persist inside cells for long time periods as they are not successfully acted upon by degradation and chaperone pathways.

It is assumed that all of these misfolded but near-native like states are non-functional. What is the evidence for this? Although in general I can see that misfolded states are likely to be non-functional, I can think of examples (multi-domain proteins) where some activity might be retained.

This assumption should be better justified and/or discussed.

Authors' response: We agree that while misfolded states are generally expected to have impaired function, it is possible that some misfolded conformations in larger proteins may retain partial activity. To better reflect this possibility, we have revised the text to refer to these states as

“less-functional” rather than strictly “non-functional.” This change clarifies that the misfolded but near-native conformations we describe are expected to have reduced functionality, without excluding the possibility that some residual activity may be retained.

Updated text: “… the protein homeostasis model should be updated to include an additional state that many proteins appear to populate: widespread entanglement-misfolded states that are soluble and <u>less-functional or even non-functional</u>, and persist inside cells for long time periods as they are not successfully acted upon by degradation and chaperone pathways (Fig. 4). The biological significance of soluble, <u>less-functional misfolded states</u>, we speculate …”

General:

The paper is very thought provoking and the question that instantly comes to mind having read the manuscript, is this: if there are a large number of NCLEs in proteins, and proteins with NCLEs are much more likely to misfold and be targeted for ubiquitination, why has Nature adopted so many structures with NCLEs? It seems very inefficient (Nature isn’t always as efficient as we think it should be), but still it is an interesting question.

Authors’ response: Based on our previous studies, we think NCLEs reflect a trade-off between folding robustness and structural/functional benefit. Specifically, we previously found that: (1) NCLEs are widespread in natural proteins, occurring in roughly half or more of globular proteins across multiple proteomes (PMID: 38296158); (2) proteins containing NCLEs are enriched in specific molecular functions and biological processes, especially catalytic, small-molecule/ion-binding, and metabolic functions, and depleted in others, indicating non-neutral evolutionary selection (PMID: 38296158); (3) NCLEs have characteristic structural and sequence features, including strong β-strand and hydrophobic-sequence biases at crossing regions, and are associated with several classes of functional sites (PMID: 40645560); and (4) NCLE-containing proteins are more prone to misfolding because these topologies introduce kinetic constraints during folding, and chaperones preferentially rescue such misfolding in essential proteins that maintain core cellular functions (PMID: 41387432).

Thus, our operating hypothesis is that NCLEs have been retained not because they are folding-efficient, but because they can provide structural and functional advantages that are sufficiently beneficial to offset their increased misfolding risk. Our recent work further suggests that, in some cases, evolution may additionally tune sequence features and proteostasis interactions to mitigate these risks rather than eliminate NCLEs altogether (PMID: 41387432). We have added discussion to the revised manuscript to make this point more explicit.

Added text: “NCLEs likely reflect an evolutionary trade-off between structural and functional advantages, on the one hand, and the ability to reliably fold on the other. Our previous studies demonstrated that NCLEs are widespread in the native structure of proteins and are enriched in proteins with catalytic and small-molecule/ion-binding functions, suggesting that these geometric features help support structural architectures required for biochemical activity (10, 36). At the same time, NCLEs introduce kinetic constraints during folding, making proteins more prone to misfolding due to premature loop closure (8). Our previous work further suggests that cellular proteostasis mechanisms can partially mitigate this cost (8). Although near-native misfolded states often evade both chaperone refolding and degradation pathways and can therefore persist in cells, the misfolding of essential proteins, which maintain core cellular functions, are more effectively avoided by chaperone systems – at least in E. coli (8). Together, these findings suggest that one reason NCLE-containing proteins may persist through evolution is because their

functional advantages can outweigh their misfolding risks, particularly when critical proteins receive greater protection from proteostasis mechanisms.”

For this type of increasingly common computational analysis of a large experimental database combined with MD simulations, it would be great if the authors could suggest an experiments/experimental approaches which could be used to test the conclusions.

Authors’ response: We now include suggestions in the Discussion Section, see below.

Added text: “Our study implies but does not establish a causal role of NCLEs in driving young-age ubiquitination. To test this mechanistic link several experimental approaches could be used. Limited proteolysis mass spectrometry (LiP–MS) (39) performed under proteasome inhibition conditions could reveal proteome-wide conformational changes associated with the accumulation of misfolded states, enabling direct comparison between structural perturbations and ubiquitination profiles. Structural methods such as cryo-electron microscopy (cryo-EM) (40) may enable direct characterization of misfolded ensembles involving entanglement changes for carefully selected targets, although such experiments will require proteins with sufficiently stable and less heterogeneous conformational states.”

One summary of the results could be "Many cellular proteins misfold and are degraded, a very large fraction of cellular proteins contain NCLEs and these are even more likely to be misfolded than non-NCLE proteins" thus "Many cellular proteins misfold and are targetted for degradation", which we already know. For me, the crux and the more interesting result is that proteins with NCLEs particularly those that misfold to structures that are near-native and which bypass the chaperone/UPS, have an effect on cellular health.

Authors’ response: We thank the reviewer for this thoughtful summary and agree that the most biologically impactful observation is that NCLE-containing proteins that misfold into near-native states may be capable of bypassing chaperone and ubiquitin–proteasome surveillance, thereby potentially impacting cellular health. Indeed, this is why we emphasize this finding in the title of the manuscript. The reviewer might be interested in several manuscripts on Biorxiv we recently posted suggesting some of those impacts of these misfolded states for aging and disease.

Reviewer #3:

We thank the reviewer for their time and thoughtful comments. We have addressed their points and believe it has strengthened the paper.

The authors bring together large-scale Ubq-MS combined with structural analysis, and folding simulations to explore questions that haven't really been addressed before: how entanglement in protein structures might influence whether newly made proteins are degraded or allowed to persist. The author's idea clearly makes a convincing case that native entanglements can bias proteins toward misfolding, and that this misfolding can have real consequences for their fate in the cell. The dataset is thoughtfully assembled, the statistical analysis is careful, and the comparison between ubiquitinated and non-ubiquitinated entangled proteins adds a layer of nuance that makes the work feel both rigorous and biologically meaningful. Overall, this is a well-executed and clearly written manuscript, I support publication after minor revisions.

Even with these strengths, there are aspects that raise some concerns:

1) In line 99, the author presents that protein ages were binned in different groups and the group which correspond less than 6 hours correspond to the proteins that are ubiquitinated after synthesis. My concern is related to whether the authors have considered extending the simulation time for this group and recalculating the analyses. Additionally, if any other protein out of this group was included as a negative control.

Authors' response: To address this concern, we have extended all simulations ten-fold, from 2 μs to 20 μs, and repeated the analyses at 1-μs intervals (new Fig. S21). The extended simulations show that the main conclusions remain unchanged: YU–E proteins consistently exhibit higher propensities to populate misfolded states involving entanglement changes than the NU–NE proteins throughout the extended trajectories. Although some proteins progressively folded toward the native state over longer times, the relative differences between protein groups persisted.

Regarding the reviewer's second question, proteins that are not ubiquitinated in the Ubq-MS dataset were used as a negative control throughout our analyses. These non-ubiquitinated (i.e., NU) proteins allow us to compare the structural features associated with proteins that are ubiquitinated shortly after synthesis with those that are not.

2) The authors have determined the entanglement of proteins, but this result is not shown. It would be helpful for the scientific community if this information were included in Table S2.

Authors' response: The entanglement assignments for the human proteins analyzed in this study have been made available in the GitHub repository associated with this work (see file name Human_AF_combined_20250614.csv). These data constitute one of the input datasets required to run the analysis script in the repository. Because the table would be very large if included in the Supplementary Information, we chose to provide it through the repository to facilitate access, reuse, and reproducibility by the community. We have added a statement in the revised manuscript indicating where this dataset can be accessed.

Updated text: "… The datasets used in the logistic regression, including the NCLE dataset of the human proteome, are available in the Github repository https://github.com/obrien-lab/Protein-entanglement-misfolding-determines-divergent-fates."

3) How the authors justify temperature-quench refolding was chosen to represent co-translational folding, since these two processes can follow quite different routes.

Authors' response: We agree that temperature-quench refolding and co-translational folding can, in principle, follow different folding pathways. To evaluate whether this difference could affect the conclusions of our study, we compared the misfolding ensembles generated by temperature-quench refolding simulations with those obtained from explicit co- and post-translational folding simulations for two representative proteins. As shown in the manuscript (see Fig. 3A and B, compared with Fig. 2A and C), these comparisons indicate that the resulting misfolded entanglement ensembles are qualitatively similar across the two folding protocols. This suggests that the temperature-quench refolding simulations capture some of the key misfolding events relevant to our analysis while allowing us to efficiently sample folding trajectories across a larger set of proteins.

To further clarify the scope and limitations of the modeling framework, we have added a discussion in the revised manuscript noting that our simulation approach is designed primarily for globular monomeric proteins and does not explicitly model the full cellular folding environment. In particular, co-translational folding on the ribosome and interactions with molecular chaperones are not explicitly represented. However, comparison with curated Reactome annotations indicates that only two of the proteins simulated here are currently listed as TRiC/CCT substrates, suggesting that most of them are unlikely to require obligate chaperonin-assisted folding. While general chaperones such as Hsp70/Hsp90 may transiently interact with nascent chains, these systems primarily prevent aggregation and promote refolding cycles and may not necessarily resolve kinetically trapped entanglement-based misfolded states.

Added text: "Like all models, our simulations have certain limitations. The simulation framework used in this study is designed to model the folding of globular monomeric proteins and therefore does not explicitly represent transmembrane proteins or proteins that are stabilized as subunits within larger complexes present in the Ubq-MS dataset. Nevertheless, it seems plausible that similar misfolded entanglement states may also occur in these protein classes, as they do contain NCLEs (e.g., one-fifth transmembrane protein domains contain NCLEs (37)) that could potentially misfold through mechanisms analogous to those observed in globular proteins (5). Further, protein folding in vivo occurs in the presence of molecular chaperones such as the Hsp70/Hsp90 systems and the TRiC/CCT chaperonin, whereas these chaperones are absent from our simulations."

4) Do the authors expect similar results in other cell types or organisms? Additionally, what factor might influence this? Have you taken into account that proteins with shallow knots might assume misfolded states?

Authors' response: Although the present study focuses on the human proteome represented in the Ubq-MS dataset, we expect the overall trends reported here to extend to other cell types and organisms. NCLEs arise from fundamental statistical properties of flexible, linear biopolymers and are widespread across the proteomes of bacteria, yeast, and humans (PMID: 38296158). Therefore, the mechanisms described here - where NCLEs increase the likelihood of forming near-native entanglement-misfolded states that influence protein quality control - are likely to be general.

That said, several factors could modulate the extent to which these effects are observed in different biological contexts. These include differences in proteostasis capacity (e.g., expression

levels of molecular chaperones and degradation machinery), translation kinetics, cellular stress conditions, and organism-specific protein sequence features that influence folding kinetics and stability. Such factors could affect both the probability of misfolding and the efficiency with which misfolded states are recognized and resolved by cellular quality-control systems – however, we expect there always to be some non-zero fraction of soluble, near-native misfolded states that persist in all organisms.

Regarding the reviewer's question about shallow knots, we did take into account proteins with shallow knots in their native state by removing them from our analyses and simulations. As mentioned in the paper, of the 2,280 mass spec observable proteins with high-quality AlphaFold predictions, 15 had knotted structures – including shallow knots – and were excluded from subsequent analysis. Non-covalent lasso entanglements are far more prevalent structural motifs and therefore represent a more common source of topological frustration during folding. We fully agree with the reviewer that shallow knots could in principle generate similar near-native misfolded states, and exploring their relationship with protein quality control represents an interesting direction for future work.

5) How confident is that the misfolded states observed in the simulations actually correspond to conformations that are not recognized by E3 ligases in vivo?

Authors' response: The simulations performed here do not explicitly model recognition by specific E3 ligases, and therefore we cannot directly determine whether the misfolded states identified in our simulations would or would not be recognized by E3 ligases in vivo. Our conclusions instead rely on indirect but experimentally grounded evidence – primarily statistical associations and trends in misfolding based on simulation models. In particular, the Ubq-MS dataset identifies proteins that become ubiquitinated shortly after synthesis versus those that do not, providing an experimental classification of proteins that are successfully targeted by the ubiquitin–proteasome system at early time points. Our simulations were designed to examine whether structural features such as NCLEs increase the propensity to populate misfolded states that correlate and could therefor influence this process. We believe that within the ensemble of misfolded structures we simulated, some are likely to be accurate and realistic while others might not be with regards to recognition/not recognition by E3 ligases in vivo. However, additional experimental data would be necessary to identify which misfolded structures are most relevant. See for example PMID: 40779622.

Importantly, the misfolded states we observe are typically near-native and compact, retaining a large fraction of native contacts. Such conformations we would expect expose fewer canonical degradation signals (e.g., extended hydrophobic surfaces or unfolded segments) compared with globally unfolded states, which may reduce their likelihood of recognition by quality-control pathways. Previous work from our group has also shown that entanglement-based misfolded states can be long-lived and structurally similar to native conformations, making them difficult for chaperones or degradation systems to distinguish (PMID: 35654797, PMID: 36471044, PMID: 37344452 and PMID: 41387432).

Therefore, while our simulations cannot directly predict E3 ligase recognition, they provide a mechanistic explanation for how certain proteins—particularly those containing NCLEs—may populate near-native misfolded conformations that could evade detection by cellular quality-control systems.

We have clarified this model limitation in the revised manuscript.

Added text: "… And our simulations do not model differences in substrate recognition by the diverse range of E3 ubiquitin ligases. Taken together, what this suggests is that while the statistical associations we observe in this study are likely arising from entanglement misfolding the effect size, or magnitude, of misfolding is likely smaller in the presence of these other cellular factors but not abolished entirely."

Reviewer comments:

Reviewer #1:

The authors have addressed my concerns using further bioinformatic approaches. However, without experimental validation demonstrating that altering the entanglement status affects ubiquitination or degradation in cells, the findings remain speculative and are more appropriate for a bioinformatics-focused journal.

Authors' response: We thank the reviewer for carefully evaluating our revised manuscript.

Reviewer #2:

The authors have gone to some lengths to address all the comments of the three reviewers. In doing so, they have produced a much more robust manuscript. It is particularly good to see that the results can largely be reproduced with different experimental datasets, and in the case where the dataset does not produce statistically significant results, that there is a reasonable explanation of why not.

Despite the fact that the study remains correlative not causal, it is an important contribution to the field, and provides an interesting hypothesis that can be researched further and validated experimentally. I would recommend the paper be published.

Authors' response: We sincerely thank the reviewer for the careful evaluation of our revised manuscript and for the positive assessment of our work. We are grateful for the reviewer's recommendation for publication.

Reviewer #3:

1) The ten-fold extension of simulations (from 2 μs to 20 μs) and the confirmation that key conclusions remain unchanged across the extended trajectories adequately addresses my concern. The clarification regarding the use of non-ubiquitinated proteins as a negative control throughout the analyses is also satisfactory.

2) I appreciate the authors' decision to make the entanglement assignments publicly available through the GitHub repository, along with the addition of a clear statement in the manuscript directing readers to that resource.

3) The comparison of misfolded ensembles between temperature-quench refolding and explicit co- and post-translational folding simulations for representative proteins is a reasonable justification for the chosen approach. The added discussion acknowledging the limitations of the modeling framework, including the absence of chaperones and the scope of the analysis to globular monomeric proteins, is a welcome addition.

4) The authors' response regarding generalizability to other cell types and organisms is well-reasoned, and I appreciate the transparent discussion of factors that could modulate the observed effects. The clarification that knotted proteins, including those with shallow knots, were excluded from the analyses is satisfactory, and I agree that exploring shallow knots in relation to protein quality control represents a worthwhile direction for future work.

5) I am satisfied with the authors' candid acknowledgement that the simulations do not directly model E3 ligase recognition, and that the conclusions rest on indirect evidence. The added text

clarifying this limitation, and the mechanistic reasoning for why near-native misfolded states may evade quality-control detection, appropriately clarify my concerns.

Authors' response: We thank the reviewer for the thorough evaluation of our revised manuscript and for the encouraging comments. We are grateful for the reviewer's thoughtful suggestions throughout the review, which have significantly improved the clarity, rigor, and presentation of the manuscript.

Thank you for the opportunity to review the manuscript titled *Protein entanglement misfolding determines divergent fates: proteasomal degradation or persistence in near-native misfolded states.* The manuscript presents and explores how changes in protein entanglement influence the fate of newly synthesized proteins. By combining Ubq-MS structural analysis, and folding simulations, the authors propose that proteins containing native NCLEs are more likely to misfold than proteins that do not contain them. The idea that topological features of protein structures can shape degradation outcomes is very interesting, and the integration of large- scale proteomics with simulation work contributes to our understanding of proteostasis.

Comments:

The authors bring together large- scale Ubq-MS combined with structural analysis, and folding simulations to explore questions that haven't really been addressed before: how entanglement in protein structures might influence whether newly made proteins are degraded or allowed to persist. The author's idea clearly makes a convincing case that native entanglements can bias proteins toward misfolding, and that this misfolding can have real consequences for their fate in the cell. The dataset is thoughtfully assembled, the statistical analysis is careful, and the comparison between ubiquitinated and non- ubiquitinated entangled proteins adds a layer of nuance that makes the work feel both rigorous and biologically meaningful. Overall, this is a well-executed and clearly written manuscript, I support publication after minor revisions.

Even with these strengths, there are aspects that raise some concerns:

1) In line 99, the author presents that protein ages were binned in different groups and the group which correspond less than 6 hours correspond to the proteins that are ubiquitinated after synthesis. My concern is related to whether the authors have considered extending the simulation time for this group and recalculating the analyses. Additionally, if any other protein out of this group was included as a negative control.

2) The authors have determined the entanglement of proteins, but this result is not shown. It would be helpful for the scientific community if this information were included in Table S2.

3) How the authors justify temperature- quench refolding was chosen to represent co- translational folding, since these two processes can follow quite different routes.

4) Do the authors expect similar results in other cell types or organisms? Additionally, what factor might influence this? Have you taken into account that proteins with shallow knots might assume misfolded states?

5) How confident is that the misfolded states observed in the simulations actually correspond to conformations that are not recognized by E3 ligases in vivo?
